# *ESR1* mutations are associated with enhanced MTORC1 signaling in a context-dependent manner

**DOI:** 10.1038/s42003-026-11096-w

**Published:** 2026-10-07

**Authors:** Tommaso De Marchi, Magdalena Kuras, Lazaro Hiram Betancourt, Sven Kjellström, Jeanette Valcich, Johan Vallon-Christersson, Christian Brueffer, Lao H. Saal, Johan Malmström, Emma Niméus

**Affiliations:** 1 Division of Surgery, Oncology, and Pathology, Department of Clinical Sciences, Lund University, Solvegatan 19, Lund, Sweden; 2 Lund University Cancer Center, Lund, Sweden; 3 Skåne University Hospital Comprehensive Cancer Center, Lund, Sweden; 4 Department of Biomedical Engineering, Lund University, Lund, Sweden; 5 Department of Translational Medicine, Lund University, Skåne University Hospital Malmö, Malmö, Sweden; 6 Swedish National Infrastructure for Biological Mass Spectrometry—BioMS, Lund, Sweden; 7 Division of Oncology, Department of Clinical Sciences Lund, Lund University, Medicon Village, Lund, Sweden; 8 Center for Interdisciplinary Research on Cancer and Equity in Women, Lund University, Lund, Sweden; 9 Division of Infection Medicine, Department of Clinical Sciences Lund, Faculty of Medicine, Lund University, Klinikgatan 32, Lund, Sweden; 10 Department of Surgery, Skåne University Hospital, Lund, Sweden

**Keywords:** Breast cancer, Proteomics, Cancer therapeutic resistance

## Abstract

*ESR1* mutations are a driver of endocrine therapy resistance in breast cancer, however their impact on protein signaling pathways and clinical outcomes remains poorly understood. We analyzed the transcriptomic, proteomic, and phosphoproteomic landscape of tumors harboring *ESR1* mutations. Here we show that *ESR1* mutated cancers exhibit a higher mutational burden associated with increased cell proliferation, and display enhanced activation of the mTOR pathway via MTORC1, suggesting an ER-driven amplification of existing oncogenic mechanisms. Analysis of an independent proteomic dataset from breast cancer cell lines further supported a convergence between MTORC1 and ER signaling programs. Integrative multi-omic data analysis incorporating features related to tumor grade, MTORC1 signaling, and *ESR1* mutations, enabled the development of a predictive signature that distinguishes patients with *ESR1* mutation and identified those at high risk of recurrence. This study provides insights into the mechanisms underlying tumor aggressiveness and poor clinical outcome modulated by *ESR1* mutations and highlights the potential of proteogenomic approaches to identify signaling pathways and molecular signatures.

## Abstract (web summary)

ESR1-mutant breast cancers show increased mutational burden, proliferation, and enhanced mTORC1 signaling, linking ER activity to tumor aggressiveness. A multi-omic signature predicts ESR1 mutations and identifies patients at higher risk of recurrence and poor outcomes.

## Introduction

Breast cancers expressing the estrogen receptor alpha (ER, *ESR1* gene) account for approximately three-quarters of all breast malignancies. Endocrine therapies, including selective ER modulators, degraders, and aromatase inhibitors, are commonly employed to treat these cancers, significantly reducing recurrence rates by up to 30%^1,2^. Despite these advances, 30–40% of ER-positive breast cancers recur due to intrinsic or acquired treatment resistance^1–3^.

*ESR1* mutations are relatively rare in primary breast tumors, occurring in approximately 1–3% of cases^4^. However, in metastatic ER-positive breast cancers treated with adjuvant endocrine therapy such as aromatase inhibitors, the prevalence of *ESR1* mutations can rise to as high as 50%, suggesting a therapy-driven selection and expansion of these mutations. These missense mutations are primarily found in the ligand-binding domain of the receptor, promoting constitutive activation leading to resistance to aromatase inhibitors and, to a lesser extent, other endocrine agents^5,6^. Many of the biological pathways enriched in *ESR1* mutants are also active in tumors with the *wt* ER, including the activation of cell proliferation pathways such as cyclin-mediated cell cycle and *MYC* signaling, which becomes activated independently of estrogen binding^7,8^. In other cases, tumors with *ESR1* mutations are associated with specific molecular features, such as the upregulation of interferon signaling or the desmosome/gap-junction genes. These features are indicative of increased invasiveness and immune evasion, both commonly observed in primary basal-like tumors and metastatic breast cancers^9–12^.

We and others have characterized transcriptomic, cistromic, and proteomic effects of *ESR1* mutations in in vitro models, revealing enrichment of cell proliferation pathways (e.g., cyclin-dependent kinases, MTORC1), DNA-binding events (e.g., CTCF, OCT1) and associated regulatory proteins (e.g., HAT1, FOXA1). These alterations are accompanied by a reduced response to therapeutic agents beyond endocrine therapies, including CDK7 inhibitors and proteasome inhibitors^8,10,11,13–16^. In addition, metabolic reprogramming and enhanced immune signaling have also been reported in *ESR1* mutant models, with interferon gamma signaling likely contributing to both immune evasion and increased proliferation through mTOR activation^7,8,16,17^.

Despite these insights, proteogenomic changes induced by *ESR1* mutations in clinical breast cancers remain underexplored. This gap is related to the limited availability of sufficiently large cohorts of tumors harboring *ESR1* mutations, particularly in early-stage cancers. Furthermore, most studies have analyzed metastatic tumors, where findings can be confounded by tumor evolution, including the accumulation of mutations in driver genes that, in turn, activate survival and proliferation pathways; clonal selection following multiple lines of therapy; and altered interactions with the microenvironment depending on the metastatic site.

In addition, previous reports have focused on transcriptomic changes without integrating protein-level information, which more directly reflects the cell's functional state and associated signaling networks. Importantly, changes in gene expression do not always translate to equivalent changes at the protein level due to post-transcriptional events such as RNA splicing and protein turnover. Proteogenomic approaches have proven effective in elucidating how genomic alterations shape both the transcriptome and proteome, as well as associated signaling pathways^18–20^. Nevertheless, a significant knowledge gap remains regarding how *ESR1* mutations translate genomic alterations into proteomic changes and downstream signaling in well-defined clinical cohorts.

To address this gap, we selected breast cancers harboring *ESR1* mutation from the SCAN-B study, a prospective, real-world and population-based multicenter early breast cancer study with available large-scale transcriptomics data^21,22^. The aim of this study was to extend the previously characterized gene expression changes^4,13^, by integrating proteome and phosphoproteome data to define how the effects of *ESR1* mutations translate from genome to proteome. Importantly, this primary tumor cohort was collected prior to adjuvant therapy, enabling investigation of ER biology without confounding effects from pharmacological treatment. The samples were analyzed using an integrated proteogenomic workflow leveraging the high accuracy and depth of protein quantitation provided by data-independent acquisition mass spectrometry (DIA-MS) and curated spectral libraries^23,24^.

Across the transcriptomics, proteomic and phosphoproteomics layers, our analysis consistently identified activation of proliferation pathways previously observed in preclinical models, as well as metabolic and survival networks driven by estrogen signaling, including mTORC1. In addition, we demonstrate that this high coverage proteogenomic dataset can be used to develop candidate predictive models in discovery cohorts, enabling the identification of patients at high risk of recurrence, in part associated with the presence of *ESR1* mutations.

## Results

### Patient selection and generation of the proteogenomic dataset

**Fig. 1:**
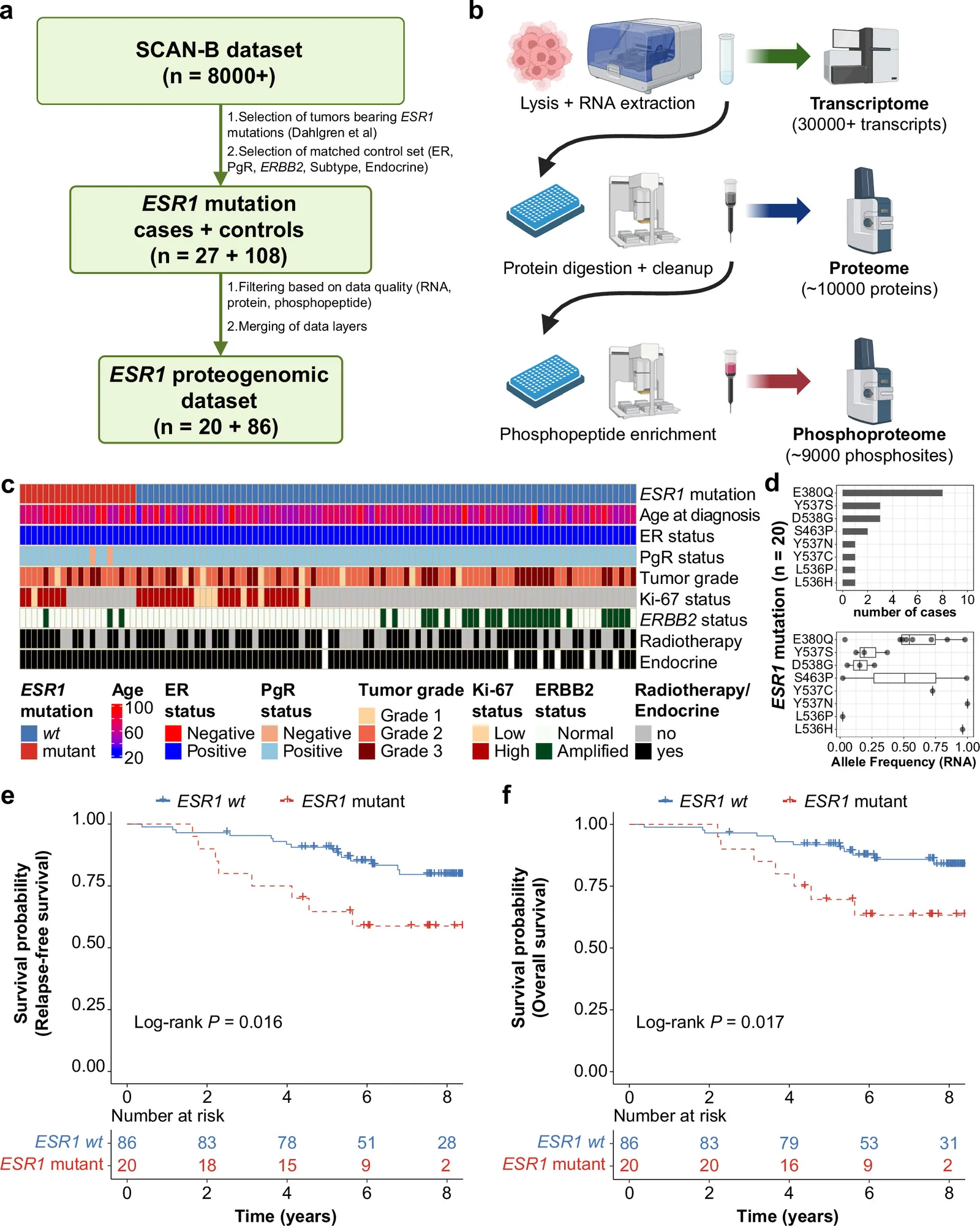
Integrated proteogenomic workflow of the SCAN-B *ESR1* dataset. **a** Schematic overview of the sample inclusion criteria. In total, 20 primary tumors harboring *ESR1* mutations and 86 control tumors were selected from the SCAN-B study, a prospective, real-world and population-based multicenter breast cancer study with associated large-scale transcriptomic data. The two groups were matched on hormonal (ER, PgR), tyrosine kinase (*ERBB2*, Her2) receptor status, adjuvant treatment and breast cancer subtype. **b** Schematic overview of the proteogenomics workflow. AllPrep RNA columns were used to extract RNA for downstream sequencing. The flow-throughs from the RNA columns were processed using an automated workflow for protein digestion, peptide mixture cleanup, and phosphopeptide enrichment. **c**, **d** Every tumor sample was annotated with clinical and histopathological characteristics derived from clinical records, including *ESR1* mutation allele frequency (*n* = 20) extracted from RNAseq data. Boxplots depict the median and interquartile range, while side bars represent the first and fourth quartiles. **e**, **f** Kaplan–Meier plots for relapse-free and overall survival and Log-Rank tests were employed to verify *ESR1* mutation association on patient prognosis. **a**, **b** were generated with BioRender.com. ER estrogen receptor, PgR progesterone receptor, SCAN-B Sweden Cancerome Analysis Network— Breast.

*ESR1* mutations have been identified in both primary and metastatic breast cancer. However, existing studies have not specifically elucidated the signaling events underlying resistance to endocrine therapy. To address this knowledge gap, we collected primary breast tumors harboring *ESR1* mutations (n = 27) from a previously described sample set^4,13^ (identified from >3200 primary breast cancers) along with a 4-times sized set of controls (n = 108) expressing *wt ESR1* (Fig. 1a). The samples were matched based on estrogen and progesterone receptor (ER, PgR) expression, ERBB2 amplification, breast cancer subtype, and adjuvant endocrine therapy^4,13^. Flowthroughs from AllPrep RNA extraction columns were employed for protein and phosphopeptide extraction followed by DIA-MS analysis, leading to the quantification of 9857 proteins and 8971 phosphosites (Fig. 1b). Due to lower data quality, 29 samples were excluded after proteomics and phosphoproteomics analysis (Fig. 1a). In the RNA dataset, quality control was performed according to standard SCAN-B consortium procedures (see *Methods* for details). The proteomic and phosphoproteomic datasets displayed comparable performance at the QC level, and ischemic time did not influence the number of identified proteins (Spearman *ρ* = -0.022, *P* = 0.831) or phosphosites (Spearman *ρ* = -0.204, *P* = 0.047). The proteome dataset had a median of 13 precursors per protein, with a minimum of 1 (Fig. S1A). Median coefficients of variation (CVs) for both proteins and phosphosites were below 10% (Fig. S1B, C), and signal intensities spanned four orders of magnitude (Fig. S1D, E). We next evaluated whether the Allprep protocol introduced biases in extracting membrane-bound and extracellular proteins over soluble ones. While intensity distributions for both groups covered the full dynamic range, membrane-bound and extracellular proteins were reduced (proteome and phosphoproteome Wilcoxon *P* < 0.001). Batch effects were subsequently assessed and corrected in both data layers (see *Methods* for details; Fig. S1F, G). The resulting datasets were then employed for all downstream analyses.

For all samples, full clinical and histo-pathological information was collected, including hormonal receptor (ER, PgR) status, *ERBB2* status, Tumor-Nodes-Metastasis, adjuvant therapy, Ki-67 status, tumor grade, relapse-free survival (RFS) and overall survival (OS; Fig. 1c and Supplementary Data 1), together with *ESR1* mutation allele frequencies derived from RNA-sequencing (RNAseq; Fig. 1d)^4^. *ESR1* mutation allele frequencies (AF) correlated with relapse-free survival (RFS; Spearman *ρ* = −0.283, *P* = 0.213) and overall survival (OS; Spearman *ρ* = −0.231, *P* = 0.312), suggesting that levels of mutated ERs impact prognosis. In contrast, Cox regression analysis showed no significant association between *ESR1* AF and RFS (Hazard Ratio (HR) = 4.61, 95% CI: 0.49 to 42.61, *P* = 0.178) or OS (HR = 2.19, 95% CI: 0.23 to 21.06, *P* = 0.497). All mutations in this cohort were located in the ligand-binding domain of the receptor and included the most common Y537S and D538G variants (Fig. 1d). Expression was largely heterozygous, as previously observed in metastatic breast cancer cases and engineered preclinical models^5,6,10^. Although key clinical and histopathological characteristics were matched between the selected ER- mutated tumors and the control cases, we observed small but significant differences in menopausal status (Fisher’s exact test *P* = 0.005) and PgR status (Fisher’s exact test *P* = 0.034; Table 1) after excluding samples due to poor MS data quality. However, neither variable was associated with RFS or OS in univariate or multivariate Cox regression analyses (Supplementary Tables 1–4), where the presence of *ESR1* mutations remained as the only factor associated with poor prognosis (Fig. 1e, f). In summary, we generated deep, ad hoc proteome and phosphoproteome datasets and integrated them with clinical records and transcriptomic profiles to investigate the multilayered signaling dynamics associated with *ESR1* mutations in both clinical and preclinical samples.

**Table 1:** Clinical and histopathological characteristics of the dataset

	*ESR1 wt*	*ESR1* mutant	*P*
*N* (%)	86 (100)	20 (100)	
Age at diagnosis^a^			
<55	28 (32.6)	3 (15)	0.173
≥55	58 (67.4)	17 (85)	
Menopausal status^a,c^			
Premenopausal	21 (24.4)	0 (0)	**0.005**
Postmenopausal	55 (64)	20 (100)	
ER status^a^			
Positive	86 (100)	20 (100)	1
Negative	0 (0)	0 (0)	
PgR status^a^			
Positive	86 (100)	18 (90)	**0.034**
Negative	0 (0)	2 (10)	
ERBB2 status^a^			
Normal	59 (68.6)	17 (85)	0.176
Amplified	27 (31.4)	3 (15)	
Ki-67 status^a,c^			
Low	7 (8.1)	1 (5)	0.66
High	23 (26.7)	7 (35)	
Tumor grade^b^			
Low	7 (8.1)	1 (5)	0.686
Moderate	47 (54.7)	13 (65)	
High	32 (37.2)	6 (30)	
Tumor size^b^			
T0+Tis	14 (16.3)	1 (5)	0.369
T1	40 (46.5)	10 (50)	
T2+Tx	28 (32.6)	9 (45)	
T3 + T4	4 (4.7)	0 (0)	
Positive lymph nodes^a^			
No	70 (81.4)	17 (85)	1
N1	16 (18.6)	3 (15)	
Adjuvant endocrine^a,c^			
No	1 (1.2)	0 (0)	1
Yes	79 (91.9)	20 (100)	
Adjuvant radiotherapy^a,c^			
No	28 (32.6)	7 (35)	1
Yes	57 (66.3)	13 (65)	

*ER* estrogen receptor, *Ki-67* tumor marker Ki67, *PgR* progesterone receptor.

^a^Fisher exact test, two-sided.

^b^ *χ*^2^ (Chi-square) test, two-sided.

^c^Missing data.

### Tumor grade, size, and proliferation are major drivers of proteogenomic variation

A key objective in multi-omics studies is to determine the relationships between the molecular data layers. To this end, we first evaluated the associations across the transcriptomic, proteomic, and phosphoproteomic datasets to assess the level of complementarity and the extent of cis/trans pathway effects. Overall positive correlations were observed between transcriptome and proteome (median Spearman *ρ* = 0.394; Fig. S2A) as well as between the proteome and phosphoproteome (median Spearman *ρ* = 0.174; Fig. S2B). These findings indicate that most changes at the transcriptome levels propagated to the proteome, and that variations in protein abundance are often accompanied by corresponding phosphorylation patterns. However, a small subset of transcript-protein and protein-phosphosites displayed negative correlations, suggesting the presence of non-genomic regulatory mechanisms. These included pathways such as the complement and coagulation cascade (Fig. S2C) and cellular responses to external stimuli, including stress signaling (Fig. S2D). These observations are consistent with previous proteogenomic studies, where distinct regulatory processes may be captured by individual data layers, underscoring the value of multi-omics approaches in providing a more complete understanding of biological mechanisms.

Next, we applied multi-omics factor analysis (MOFA)^25^ to disentangle the main axes of heterogeneity that were specific to individual data modalities or shared across multiple layers. Robustness analysis (see *Methods*) showed that the model converged in terms of variance explained. In addition, subsampling analysis showed that latent factor scores were highly consistent between full and reduced datasets, with near-perfect correlations for Factor 1 (Spearman *ρ* > 0.98 across all iterations). These results indicate that the MOFA decomposition was highly robust, with factor estimation not driven by specific sample subsets.

**Fig. 2:**
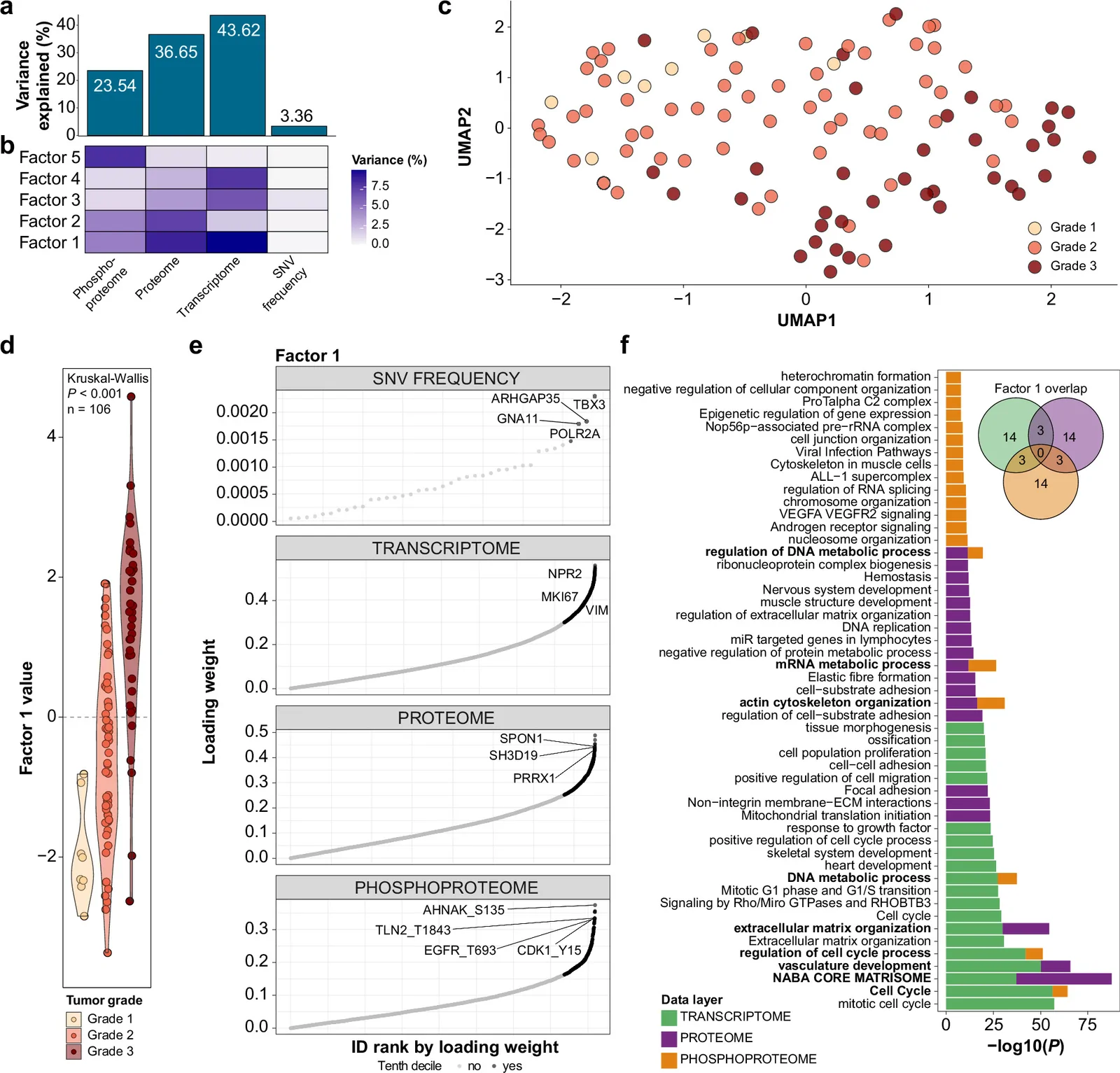
Multi-omics factor analysis of the SCAN-B *ESR1* dataset. MOFA was employed on the combined data layers, including SNV frequency, transcriptome, proteome and phosphoproteome. This analysis defined the major contributors to overall dataset variation (**a**). Decomposition of this variation into learned factors showed the first five to be the ones most contributing to the dataset variance. Color intensity indicates the percentage of variation (**b**). UMAP visualization of all learned factor values showed clustering according to tumor grade (**c**). The association of tumor grade with dataset variation was confirmed in the individual factor-level analysis, where factor 1 values showed a significant increase with tumor grade (**d**; Kruskal-Wallis test, two-sided *P* = 4.647 × 10^-10^). Loading weights for every data layer were extracted and plotted in waterfall plots (**e**). The top 10% features of each data layer (except SNV frequency) were analyzed in Metascape (**f**; bold text on the *y*-axis of the bar chart represents pathways enriched in at least two data layers). MOFA multi-omic factor analysis, SNV single-nucleotide variant, UMAP Uniform manifold approximation and projection.

In this analysis, more than 80.3% of the variation was defined by the transcript and protein data (Fig. 2a), while phosphoproteomics and SNV frequency accounted for 23.5% and 3.4%, respectively. The first five factors were associated with most of the variation in the dataset (Fig. 2b), with transcript and protein abundance associated with factors 1–4. In contrast, factor 5 was mainly associated with phosphoproteomic variation. These findings suggest that transcriptome and proteome dynamics largely contribute to shared axes of variation, whereas protein phosphorylation captures distinct and modality-specific regulatory processes.

To identify clinical and histopathological features associated with the observed variation, we visualized the MOFA factors using UMAPs. In this cohort, tumor grade (Fig. 2c) and *ERBB2* amplification (Fig. S3A) had the strongest impact on the molecular data layers, with a significant co-occurrence between intermediate/high grade and *ERBB2* amplification (*χ*^2^ *P* = 0.002). Notably, no distinct clustering was observed for *ESR1* mutational status (Fig. S3B), indicating that the impact of ER mutants is more subtle compared to the broader tissue remodeling linked to tumor grade or major proliferation pathways such as *ERBB2* amplification. Taken together, these data support a model where *ESR1* mutations exist on top of major pathways driving cancer progression and possibly exacerbating their effects due to constitutively activated ER signaling.

We next examined the individual factors and found that factor 1 was significantly associated with high tumor grade (*P* < 0.001; Fig. 2d), *ERBB2* amplification (*P* < 0.001; Fig. S3C), high Ki-67 (*P* < 0.001; Fig. S3D), and larger tumor size (*P* = 0.002; Fig. S3E). The remaining factors showed no significant associations with the available clinical or histopathological variables, with the exception of factor 2, which was also linked to *ERBB2* amplification (*P* = 0.039; Fig. S3C). We then extracted the loading weights from the MOFA model and selected the 90th percentile within each data layer to identify SNV frequency, transcripts, proteins, and phosphosites most strongly associated with each factor (Fig. 2e). Among the top-ranking features were well-established proliferation markers (e.g., CDK1, MKI67) and extracellular matrix (ECM) components (e.g., VIM). This pattern indicates a dual axis of proliferation and matrix remodeling, which was supported by functional enrichment analysis using Metascape^26^ (Fig. 2f). Collectively, these results indicate that the dominant molecular variation in this cohort reflects a tumor grade increase in proliferation-associated factors such as CDK1/2 or MKI67, alongside a concomitant decrease in ECM components i.e. collagens (Fig. S4), while *ESR1* mutations did not directly contribute to the top 5 factors of variation.

Given that tumor grade emerged as the primary driver of variation, we investigated whether key cancer pathways were directly linked to grade and whether *ESR1* mutations modulated their activation. Hallmarks pathways were selected based on GSVA scores that showed consistent linear trends with tumor grade across both the RNA and protein layers (Fig. S5A). We then assessed the additional impact of *ESR1* mutations by employing linear models. Pathways most affected by *ESR1* mutations included MTORC1 signaling (positive impact; Fig. S5B) and UV RESPONSE DN (negative impact; Fig. S5C). However, interaction analyses did not reveal significant synergistic effects between *ESR1* mutations and grade-associated pathways (*P* > 0.05). Taken together, these results indicate that *ESR1* mutations may potentiate already existing cancer-related pathways such as proliferation and survival, but largely operate in parallel to dominant determinants of tumor biology, particularly tumor grade.

### Heightened mutational burden in ESR1-mutated tumors is dependent on cell proliferation

The findings above suggest that ER has a modest effect variation across the dataset compared to grade and is more likely to contribute to pre-existing oncogenic mechanisms. Accordingly, we conducted an ad hoc analysis to identify the molecular changes associated with *ESR1* mutations. Mutations in the ER can disrupt DNA-binding events^8,10^, alter gene expression^12^, and protein signaling^13^ and trigger cascades that intersect with multiple pathways, including PI3K. However, it remains unclear whether co-occurring mutations in key cancer driver genes reflect underlying cross-talk mechanisms or synergistic effects that enhance the activity of mutant ER. To investigate this, we extracted all single-nucleotide variant calls in COSMIC genes from the RNAseq data to determine whether additional genetic changes typically occurred together with *ESR1* mutations, potentiating or complementing estrogen signaling.

**Fig. 3:**
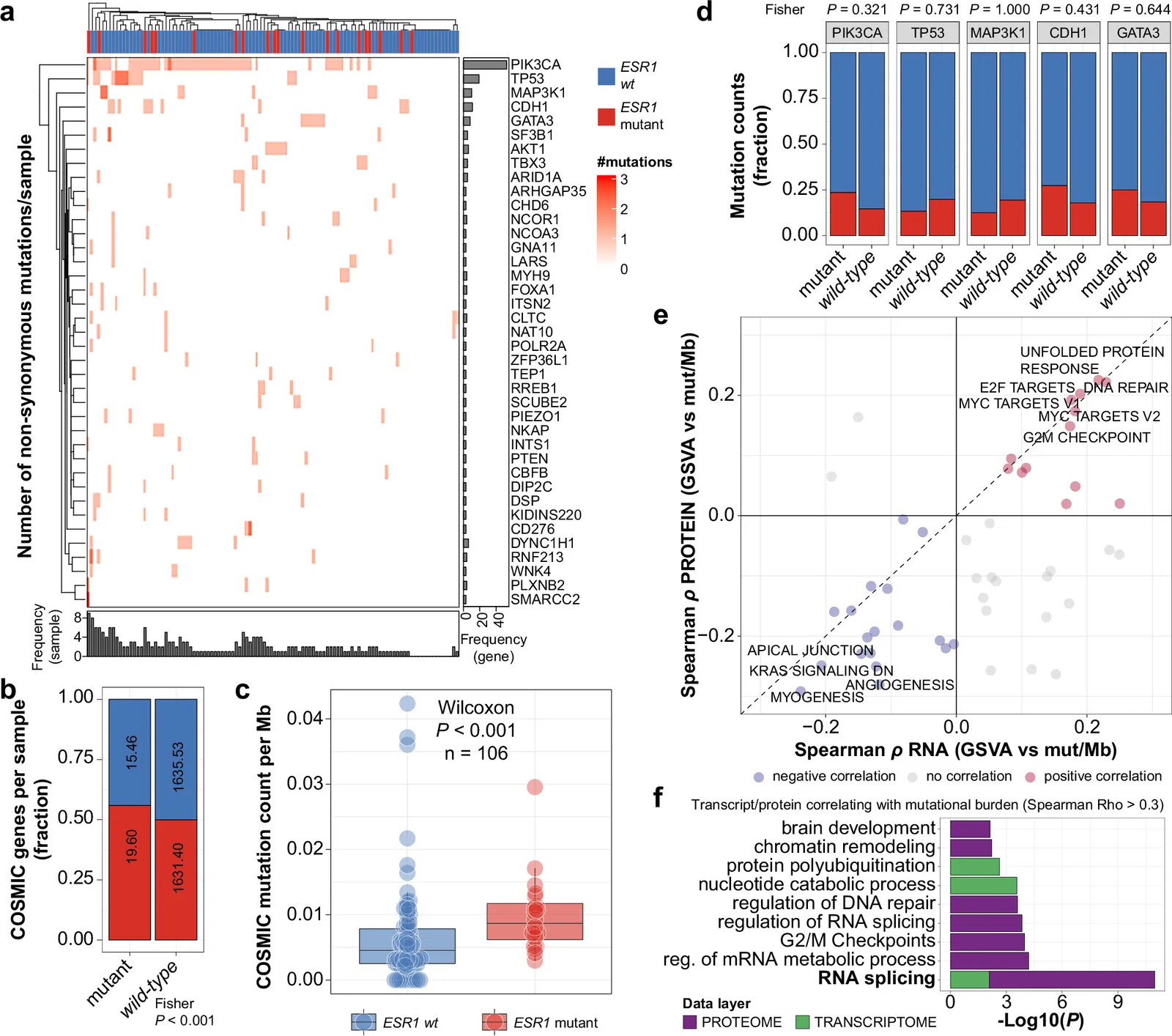
Mutational call analysis in association with *ESR1* mutations. We extracted the mutational repertoire based on the COSMIC gene census to assess the relationship between *ESR1* mutations and sample types (i.e., missense mutations). Hierarchical clustering of mutational occurrence (**a**). Bar plot to the right side of the heatmap indicates the most occurring mutations in the dataset, while the bar plot at the bottom of the heatmap indicates the number of mutations in each tumor specimen. **b** shows stacked bar plots indicating the fraction of the average number of mutated COSMIC genes in the *ESR1 wt* and mutated tumors (Fisher’s exact test, two-sided *P* = 4.398 × 10^-5^). **c** displays the number of COSMIC mutations per Mb in the *ESR1 wt* (*n* = 86) and mutated (*n* = 20) samples (Wilcoxon test, two-sided *P* = 4.611 × 10^-4^). **d** displays frequencies of the most mutated genes in the dataset (top 5) against *ESR1* mutation frequency (Fisher's exact test, two-sided). Fisher's exact tests were employed to assess any co-occurrence, but no concomitant variant was observed to occur with *ESR1*. **e** depicts the scatterplot of Spearman coefficients between TMB and GSVA-derived pathway scores. Sample-wise GSVA scores were calculated for the Hallmarks dataset from both the transcriptomic and proteomic datasets. The GSVA scores were then correlated with the tumor mutational burden (TMB). The Spearman *ρ* coefficients were then plotted in a two-dimensional plot. Positive correlations in both proteomic and transcriptomic datasets are colored red, and negative correlations are colored blue. **f** displays metascape analysis of transcripts and proteins highly correlating with TMB, which were used to confirm results in (**d**). **e** depicts displays enriched pathways for the transcriptome (green) and proteome (purple) data layers. COSMIC Catalog of Somatic Mutations in Cancer, GSVA gene set variation analysis, TMB tumor mutational burden.

Excluding *ESR1* mutations, we identified a list of 1651 missense variants, of which 39 were detected in more than two samples (frequency per gene range: 2.83% to 50.00%). As mutation calling from RNAseq is generally prone to a higher rate of false positive detections due to shallow sequencing, technical artifacts, or lack of intronic information^27,28^, we restricted the analysis to known cancer mutations (see *Methods*). Hierarchical clustering of non-synonymous mutation frequencies revealed enrichment in genes commonly altered in luminal breast cancer, such as PIK3CA and GATA3, although no clear separation between ER mutated and *wt* tumors was observed (Fig. 3a). Notably, tumors harboring *ESR1* mutations exhibited a significant higher mutational burden compared to *wt* tumors (median frequency ratio: 1.92, Wilcoxon *P* < 0.001; Fig. 3b, c), although no individual gene was found to be consistently co-mutated with *ESR1* (Fig. 3d). This findings indicate that increased mutational burden observed in ESR1 mutant tumors may arise from downstream effects of estrogen signaling, likely linked to enhanced proliferation and concurrent DNA replication. Consistent with this, standard clinical variables such as age at diagnosis (Spearman *ρ* = 0.094, *P* = 0.337) and KI67 levels (Spearman *ρ* = −0.039, *P* = 0.814) showed no significant correlation with the tumor mutational burden.

To further explore which biological pathways might underlie the differences in mutational burden between *ESR1* mutant and *wt* tumors, we calculated sample-wise GSVA scores^29^ for the Hallmarks using both transcriptomics and proteomics data, and correlated these to tumor mutational burden (TMB). GSVA scores for G2M checkpoint, DNA repair and unfolded protein response showed positive correlations with TMB (Fig. 3e). In contrast, negatively correlated gene sets included angiogenesis and apical junction, which are typically not associated with mutational processes. Metascape analysis of the most strongly correlated transcripts and proteins supported these observations (Fig. 3f)^26^. These patterns could potentially reflect differences in ER abundance between *ESR1* mutant and *wt* tumors. However, analysis of the transcriptomic and proteomic data revealed a strong correlation between ER transcript and protein levels, with no differences between mutant and *wt* cases (Fig. S6A). Furthermore, there was no significant association between *ESR1* mutant allele frequencies and *ESR1* transcript or ER protein abundance, indicating that the effect is independent of ER expression levels (Fig. S6B, C). Overall, these results suggest that *ESR1* mutant tumors exhibit a higher mutational burden that is associated with cell proliferation-related pathways such as E2F and MYC signaling, supporting a link between proliferative activity and elevated mutation rates.

### ESR1-mutated cancers display higher proliferation through pathway convergence

Next, we leveraged our transcriptomic, proteomic, and phosphoproteomic datasets to identify pathways enriched in the *ESR1*-mutated tumors that could function as cofactors promoting tumor growth or represent signaling pathways converging with ER activity. We first examined transcripts and proteins known to be associated with the ER pathway^30–32^. At the transcript level, we observed modest increases in ER-cofactors, e.g., FOXA1 and GATA3, as well as MKI67 (Fig. S7A), although these differences did not reach statistical significance. Consistent with this, only minor changes were detected in the protein phosphorylation states of these proteins (Fig. S7B) as well as in the early and late estrogen response gene sets (Fig. S7C). Together, these findings indicate that *ESR1* mutations do not induce substantial changes in abundance and phosphorylation of canonical ER pathway components, in line with previous observations from cell models expressing mutant *ESR1*^8,13^.

**Fig. 4:**
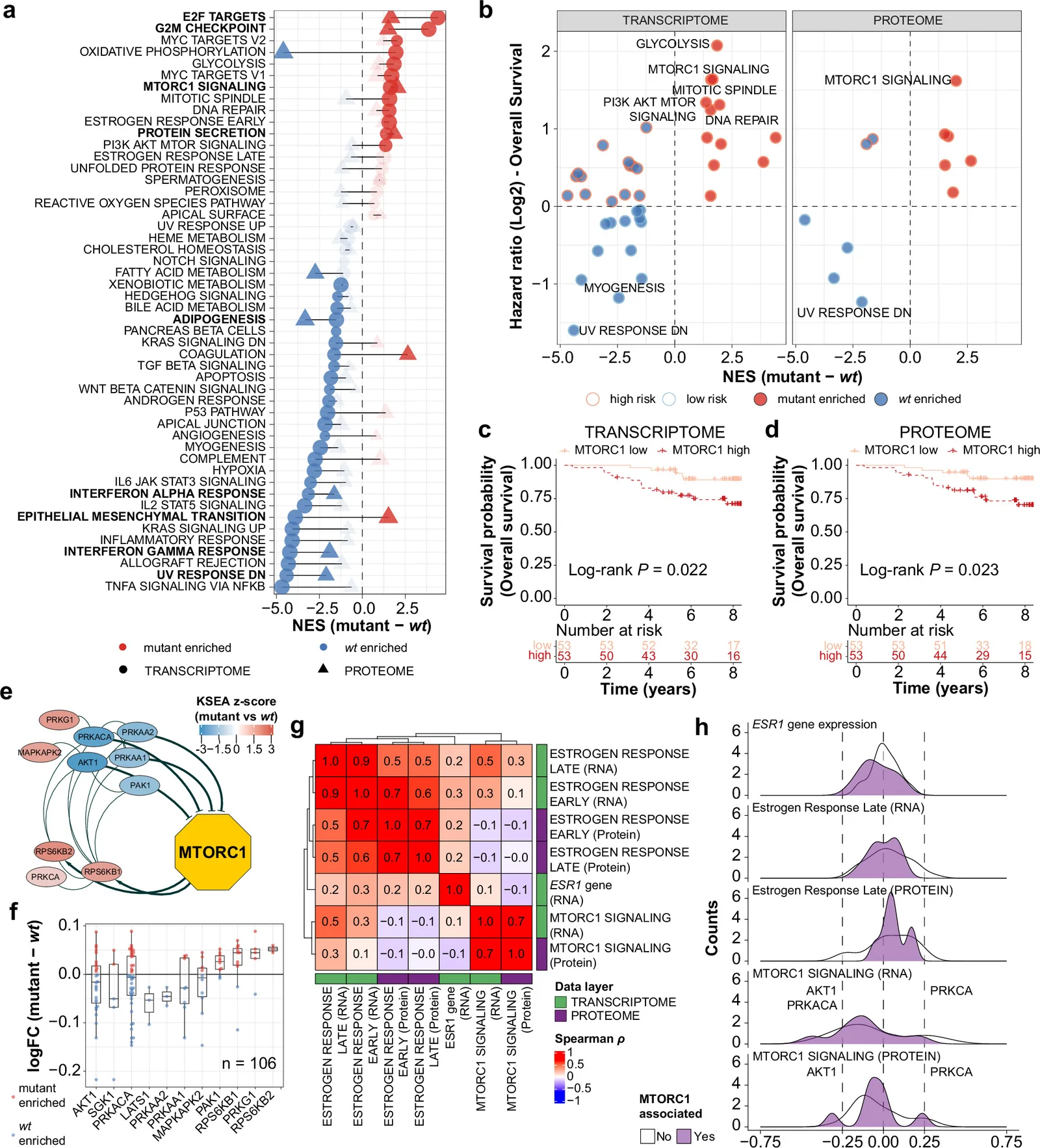
Integrated analysis of *ESR1* mutations in primary breast cancers. **a** Normalized gene set enrichment score (NES) was calculated between *ESR1* mutants and *wt* tumors for the transcriptomic and the proteomic data layers. The NES score for each gene set was then plotted for both proteomics (triangles) and transcriptomics (circles). Non-significant gene sets are opaque for clarity. Pathways in bold represent significant enrichment in both data layers (**a**). We calculated sample-wise Hallmarks pathway scores using GSVA and performed Cox regression analysis for Relapse-free Survival (RFS) for each pathway (transcriptomics and proteomics). Cox regression-derived HR were log-transformed and plotted against the NES of each pathway out of **a** in scatter plots. Pathways enriched in GSEA analyses and associated with high/low risk of relapse are located in the first and third quadrants of the plots (**b**). We extracted the GSVA scores for the pathway that was consistently enriched in *ESR1* mutants in both the transcriptome and proteome layers and significantly associated with poor prognosis, split the patient groups based on the median GSVA score and performed KM analysis (**c**, **d**). We performed KSEA analysis on the phosphoproteomic data to derive kinases enriched in *ESR1* mutants and controls. Cytoscape was employed to generate an enrichment map depicting KSEA results for the MTORC1 pathway-associated kinases (**e**). Phosphopeptide intensities of enriched kinase (**e**) substrates were then plotted in box plots (**f**) for all samples (*n* = 106). GSVA scores for the transcriptome and proteome were extracted along with *ESR1* transcript abundance, and a correlation matrix (method: Spearman) was computed. Hierarchical clustering was then performed and plotted as a heatmap (**g**). Phosphosites linked to MTORC1-associated kinases were assembled into GSVA scores (see *Methods*) and correlated to *ESR1* transcript abundance (top), MTORC1 signaling RNA-based (middle) and protein-based (bottom) GSVA scores (**h**). The top five kinases with the strongest positive and negative correlations are shown in the middle and bottom panels. GSEA gene set enrichment analysis, GSVA gene set variation analysis, HR hazard ratio, KM Kaplan–Meier, KSEA kinase-substrate enrichment analysis, NES normalized enrichment score, OS overall survival, RFS relapse-free survival.

In the next step, we performed GSEA to identify pathways activated downstream of *ESR1* mutations. Both the transcriptomics and the proteomics data revealed enrichments of pathways related to cell cycle progression (e.g., E2F targets, G2M checkpoint) and cell growth (e.g., MTORC1 signaling; Fig. 4a) in ER mutant tumors. These pathways have previously been reported in cell line models harboring *ESR1* mutations^8,10,13,16^ and may act as co-drivers of the enhanced survival and proliferative capacity observed when compared to *wt* cells. In contrast, downregulated gene sets were primarily associated with inflammation and immune signaling, including interferon pathways. Consistent with previous proteogenomic studies^24^, we observed discrepancies in pathways not directly governed by transcriptional regulation, such as epithelial-to-mesenchymal transition and coagulation, where enrichment at the protein-level likely reflects interactions with the tumor microenvironment rather than changes driven by transcription factor activation.

To address the high degree of redundancy among enriched pathways, we extracted transcripts and proteins from significant gene sets and consolidated them into seven functional clusters, which were visualized using UMAP (Fig. S8A, B). This analysis revealed a clear increase in clusters related to proliferation and the tumor microenvironment, suggesting enhanced tumor expansion, invasion and interaction with surrounding tissue (Fig. S8C–F). These findings were further supported by interrogation of the Reactome database, which identified differential enrichment of two distinct networks, DNA and cell replication and cell adhesion (Fig. S9). These results indicate that breast cancers harboring *ESR1* mutations exhibit increased proliferative capacity when compared to *wt* tumors, consistent with the shorter relapse time and reduced survival observed in this subgroup (Fig. 1e, f).

To determine which of these pathways were associated with poor prognosis, we performed Cox regression analyses for RFS and OS (Fig. 4b–d and Fig. S10) using GSVA scores stratified into high- and low-expression groups based on the median. We then plotted the resulting hazard ratios against the enrichment scores from our GSEA analyses. This approach revealed that MTORC1 and UV response pathways were consistently enriched across both transcriptome and proteome datasets (Fig. 4b and Fig. S10A). Elevated MTORC1 signaling was significantly associated with shorter RFS (transcriptome HR = 2.58, *P* = 0.037; proteome HR: 2.54, *P* = 0.039) and OS (transcriptome HR = 3.11, *P* = 0.029; proteome HR: 3.07, *P* = 0.031). In contrast, higher UV response pathway activity was associated with improved prognosis, although not consistently significant (RFS not significant; OS transcriptome HR = 0.33, *P* = 0.033; OS proteome HR = 0.42, *P* = 0.083). Kaplan-Meier analyses confirmed these findings, showing that increased MTORC1 signaling corresponded to faster relapse and reduced survival at both transcript and protein levels (Fig. 4c, d and Fig. S10B, C). Conversely, the UV response pathway, which was downregulated in *ESR1* mutant tumors and showed favorable hazard ratios, showed borderline significance in our survival analyses (Fig. S10D–G). These data indicate that the MTORC1 signaling pathway, which stands at the crossroads between metabolism and cell growth (reviewed in ref. ^33^) and of which several studies have reported significant enrichments in *ESR1* models^7,8,16,17^, might be one of the major drivers of tumor proliferation and invasiveness whose signaling might be potentiated by *ESR1* mutations.

### MTORC1 and estrogen signaling converge to enhance proliferation in ESR1-mutated cancers

To further investigate the relationship between MTORC1 signaling and *ESR1* mutations, we analyzed the phosphoproteomic dataset using Kinase-substrate enrichment analysis (KSEA). Here, KSEA scores indicated activation of the downstream signaling factors of the MTORC1 complex, including RPS6KB1-2, alongside inhibition of the upstream inhibiting kinases such as AKT1 and PRKACA (Fig. 4e, f)^34^. These results are consistent with observations from the transcriptomic and proteomic analysis, collectively providing multi-layered evidence from MTORC1 activation.

We next examined the relationship between MTORC1 signaling and ER downstream activity. A Spearman correlation matrix was computed between *ESR1* transcript levels and GSVA scores for the Estrogen Response and MTORC1 pathways at both RNA and protein levels. While MTORC1 signaling and Estrogen Response (Early/Late) GSVA scores were found to be positively correlated (average transcriptome/proteome intra pathway Spearman *ρ* > 0.5) and clustered according to pathway, only a weak-to-moderate positive correlation was observed between Estrogen Response and the MTORC1 signaling pathway. This suggests that MTORC1 activation is not directly driven by *ESR1* transcript abundance, but rather may reflect downstream ER activity (Fig. 4g).

To further assess pathway co-regulation at the kinase level, we correlated phosphosites regulated by MTORC1-associated kinases (Fig. 4e) with *ESR1* transcript levels, and the levels of Estrogen Response and MTORC1 pathways. No significant correlation was observed between MTORC1-related kinase activity and *ESR1* transcript levels, although a subset of kinases showed weak correlations, including AKT1 (negative correlation, Spearman *ρ* < −0.2), PRKCA (positive correlation, Spearman *ρ* > 0.2; Fig. 4h). These results indicate that MTORC1 pathway activation is only partially influenced by ER signaling and is likely potentiated by *ESR1* mutations in a context-dependent manner.

**Fig. 5:**
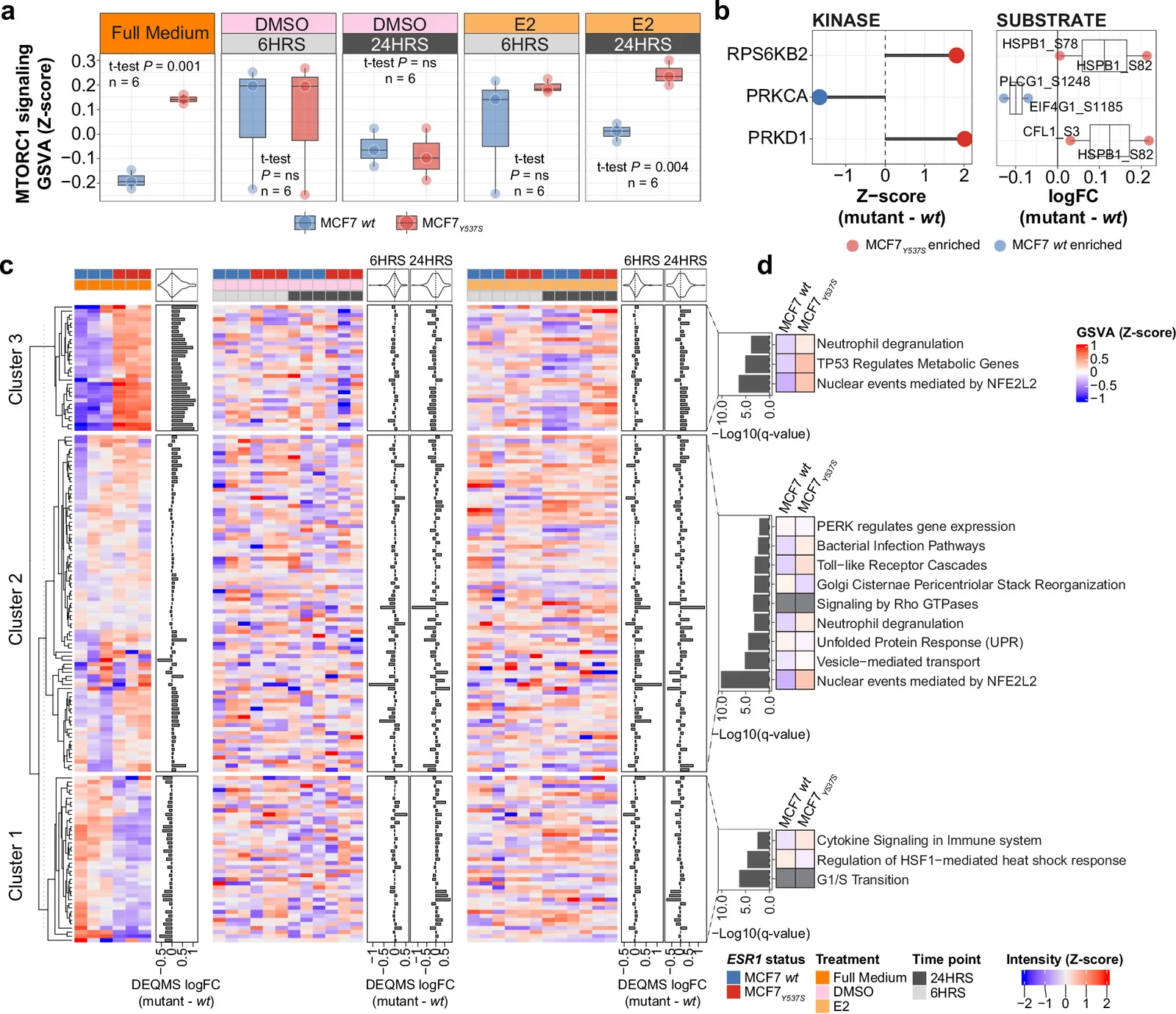
Preclinical model validation of MTORC1 and association with estrogen signaling. We mined the proteome data of our previously published dataset (ProteomeXchange ID: PXD046446^13^) and extracted the proteome and phosphoproteome data of the MCF7 models (i.e., isogenic *wt ESR1* and Y537S mutant). We calculated the GSVA scores for the MTORC1 pathway for all culture conditions (i.e., full medium, estrogen deprivation (DMSO), and estrogen stimulation (E2)) and time points (i.e., 6 h, 24 h). MTORC1 GSVA scores were plotted in boxplots for *ESR1* mutant and *wt* cells (*n* = 3 per each cell line, time point, and condition) and were compared by *t*-test (two-sided; **a**). Enrichments for MTORC1-associated kinases were derived from the phosphoproteomic data using KSEA (**b**, left). Phosphopeptide abundances of kinase substrates were also plotted (**b**, right). Proteins belonging to the MTORC1 pathway were extracted, and their abundances were plotted in heatmaps for every treatment condition and time-point (**a**) to derive clusters that were specifically enriched (**c**). Bar charts on the right of each heatmap depict the Log2 Fold-Changes of each protein (mutant over *wt*) for every culture condition. Violin plots on top of the bar charts depict the distribution of Log2 Fold-Changes for each condition and time point. A total of three distinct protein clusters were defined via *k*-means clustering. Proteins belonging to each cluster were analyzed by Metascape to define associated pathways, which were depicted in bar charts (**d**, left). GSVA scores for metascape pathways were then calculated using GSVA, and their expression was plotted as a heatmap for full medium MCF7 *wt* and MCF7Y537S (**d**, right). DMSO dimethyl sulfoxide, E2 estradiol, GSVA gene set variation analysis, KSEA kinase-substrate enrichment analysis.

To further verify these findings, we performed integrative proteomic analysis of prior cell line results by reanalyzing previously generated data to assess MTORC1 pathway enrichments^13^. GSVA-based analysis revealed a significant enrichment of the MTORC1 pathway in MCF7_Y537S_ mutant cells compared to their isogenic *wt* counterpart under full medium conditions. A similar pattern was observed following 24 h estrogen stimulation in hormone-depleted media (Fig. 5a), supporting a link between MTORC1 activation and estrogen signaling.

Phosphoproteomic data from full medium conditions further corroborated these observations, showing significant enrichment of kinases, including PRKCA (decreased), RPS6KB2 and PRKD1 (increased; Fig. 5b). Given the complex regulation of the MTORC1 pathway and its involvement in diverse cellular processes such as cell survival, cytoskeleton, metabolism, or autophagy, we next examined the full MTORC1 gene set. Hierarchical clustering of MTORC1 pathway proteins across different culture conditions revealed 3 distinct clusters (Fig. 5c). Notably, one (cluster 3) contained several proteins that were consistently more abundant in the MCF7_Y537S_ compared to *wt* cells, mirroring the observed increase GSVA score for the pathway (Fig. 5a). Metascape analysis for this subset revealed an overrepresentation of proteins involved in metabolism and oxidative stress, including targets of TP53 and pathways associated with NFE2L2 signaling (Fig. 5d).

To evaluate the potential translational relevance of these findings, we performed Connectivity map analysis^35^ on our cell line proteomic dataset (MCF7 and T47D)^13^ to identify compounds that would target *ESR1* mutant phenotypes and reverse their proliferative signatures. This analysis highlighted mTOR, proteasome, and tubulin inhibitors as the most over-represented drug classes (Fig. S11A). Notably, cluster 3 included TUBA4A, which is targeted by FDA-approved agents, indicating that tubulin inhibition may act synergistically with mTOR pathway targeting. To further support this observation, we analyzed the RNAseq data from a compendium of *ESR1* mutant clones (GSEA147745)^10^. This confirmed the association between MTORC1 signaling and estrogen response across 15 *ESR1* mutant models (Fig. S11B) and further indicated that tubulin inhibition could represent a complementary strategy for reversing the mutant phenotype (Fig. S11C). Taken together, these results show that MTORC1 signaling is enhanced following estrogen stimulation and further potentiated by *ESR1* mutations in in vitro models, particularly along the metabolic axis of the pathway. Integration of these findings with observations from the SCAN-B clinical cohort and MCF7 models suggests that *ESR1* mutations promote survival and proliferative signaling in a context-dependent manner.

### Integrated predictive models of clinical outcome

In this study we found that tumor grade is a major determinant of transcript and protein variation within this cohort (Fig. 2), and that MTORC1 signaling is a key pathway potentiated by *ESR1* mutations and associated with both tumor growth and patient outcomes (Figs. 4 and 5). Together, these factors appear to play critical roles in tumor progression. To evaluate whether this information could be used for patient stratification, we integrated MTORC1 signaling levels based on GSVA based, *ESR1* mutation status, and tumor grade to stratify tumors into low- and high-risk groups. High-risk tumors were defined by elevated MTORC1 activity, presence of *ESR1* mutations, and high tumor grade. Kaplan–Meier (KM) analyses revealed significant differences between the high- and low- risk groups, although this pattern was not entirely consistent across all data layers (Fig. S12).

**Fig. 6:**
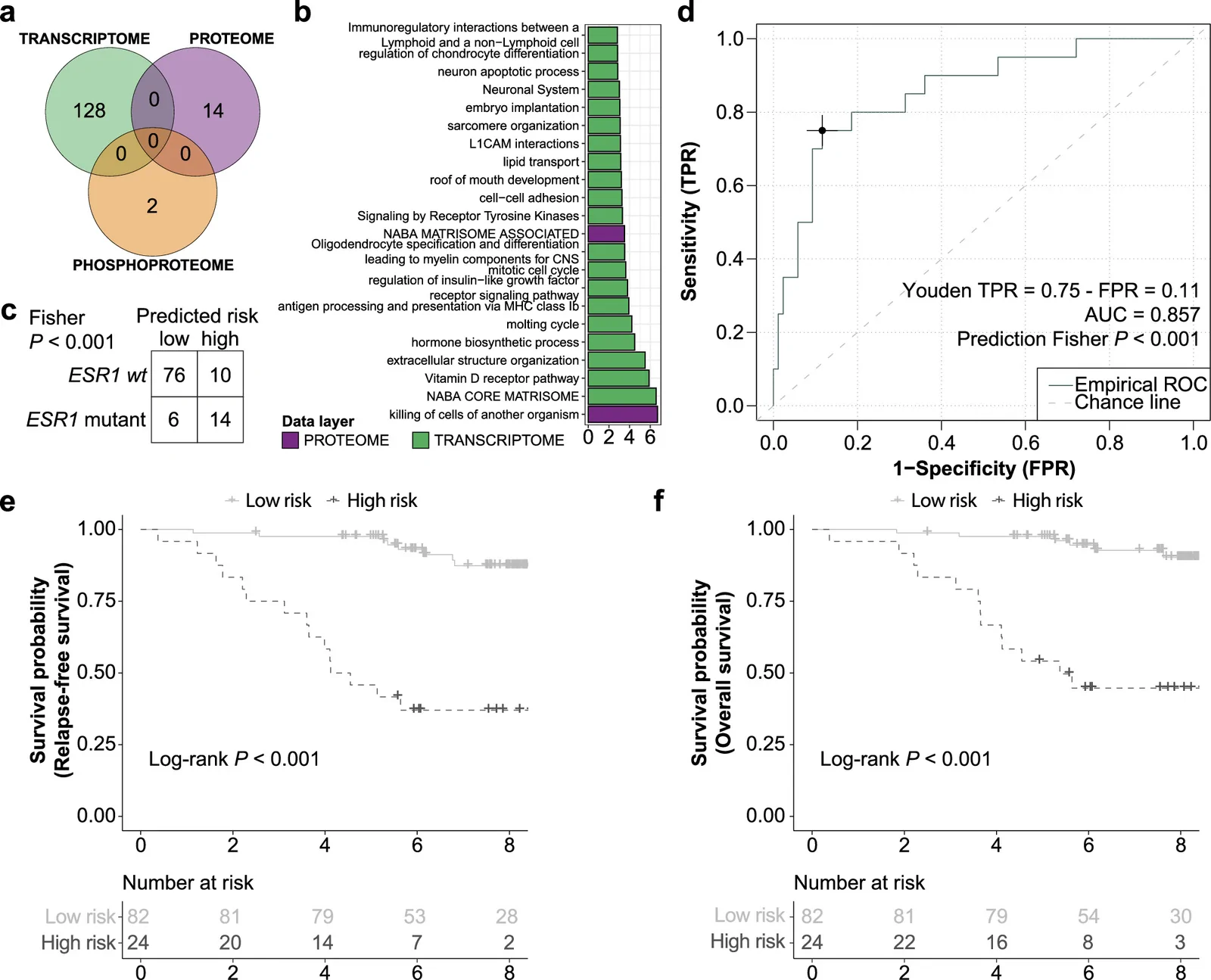
Multi-omic model for prediction of breast cancer prognosis. We extracted transcriptomic, proteomic, and phosphoproteomic features significantly associated to *ESR1* mutants and poor prognosis (see *Methods* and Fig. S6 for details). Overlap between extracted features across data layers was depicted in Venn diagrams (**a**). Metascape analyses were performed for all extracted features, and enriched pathways were plotted in bar charts (**b**). We next combined the features from all data layers to generate a multi-omic predictive model, which defined low and high-risk patients (based on the Youden index; see *Methods* and *Results* for details). We employed a contingency table and Fisher's exact test (two-sided *P* = 2.652 × 10^-5^) to define the association between predicted risk categories and *ESR1* mutations (**c**), while the model quality was evaluated by plotting its sensitivity and specificity in a Receiver Operator Characteristic (ROC) plot and calculating the area under the curve (AUC; **d**). Fisher exact test (two-sided *P* = 3.722 × 10^-7^) was employed to measure the association between observed and predicted groups. Performance of the model was evaluated by Kaplan–Meier plots and Log-rank tests in association with Relapse-free survival (RFS, Log-rank P = 1.303 × 10^-10^; **e**) and overall survival (OS, Log-rank P = 4.732 × 10^-9^; **f**). KM Kaplan–Meier, OS overall survival, RFS relapse-free survival.

We then hypothesized that a data-driven proteogenomic approach, using features that were strongly associated with *ESR1* mutations and clinical outcomes, could further improve the prediction of patient prognosis. To test this, we extracted 128 transcripts, 14 proteins and 2 phosphosites that were enriched in *ESR1* mutant cancers based on log-ratio and showed the strongest association with prognosis according to Cox regression p-value (see *Methods* for details and Fig. 6a). Functional enrichment analysis using Metascape revealed that these features were primarily involved in growth factor pathways (e.g., CNTFR) and interactions with the extracellular environment (e.g., MMP16; Supplementary Data 2 and Fig. 6b). Notably, there was only partial overlap between these features and those contributing most strongly to factor 1 in the MOFA analysis (transcriptome overlap *n* = 42; proteome and phosphoproteome n = 0), suggesting that the primary drivers of variation are not fully aligned with determinants of clinical outcome.

For each data layer, we aggregated the intensities of the selected features to generate a patient-specific score, which was subsequently used to generate receiver operator characteristic (ROC) curves. The optimal cutoff for each model was defined using the Youden index, enabling classification of patients into high and low-risk groups. The areas under the curve (AUC) values for each model based on individual data layers ranged from 0.694 to 0.781 (Fig. S13A–C), with comparable performances in identifying both high-risk patients and tumors harboring *ESR1* mutations (Fig. S13D–F). Survival analyses for RFS and OS demonstrated significant differences between the predicted risk groups, particularly for the proteome-based model (Fig. S13G–L). To assess robustness, we performed leave-one-out cross-validation (LOOCV), which yielded AUC values consistent with those obtained from the full dataset (Supplementary Table 5), indicating minimal overfitting. We next evaluated the predictive performance of these signatures in large public cohorts. Due to the limited variability of large-scale proteomics datasets in breast cancer, this analysis was restricted to the RNA-based model. In the METABRIC^36^, TCGA^37^, and SweBCG91RT^38^ cohorts, the model consistently stratified patients into high- and low-risk groups with significant differences in survival across the cohorts (Fig. S14A–C), as well as within the Luminal A/B and Her2 subtypes (Fig. S14D–F).

We next integrated features from all data layers into a single model, which outperformed most models based on individual data types in predicting both high-risk patients and *ESR1* mutation status (Fig. 6c–f and Fig. S15, Supplementary Table 5). The integrated model showed significantly higher discriminatory performance compared with the RNA-only model (AUC 0.844 vs. 0.685; paired DeLong test *P* = 0.003; Holm-adjusted *P* = 0.006) and the phosphoproteomic model (AUC 0.844 vs. 0.648; *P* = 0.002; Holm-adjusted *P* = 0.005). In comparison with the protein model, the integrated model showed a numerically higher AUC (0.844 vs. 0.752), but this difference did not reach statistical significance (*P* = 0.120).

Notably, this integrative model remained significant in multivariable Cox regression analyses (RFS HR = 9.19, 95% CI: 3.43 to 26.64, *P* < 0.001; OS HR = 8.87, 95% CI: 2.97 to 26.46, *P* < 0.001; Supplementary Tables 6 and 7). In summary, these results indicate that a combined proteogenomic feature panel provides generally higher predictive performance than single-layer signatures for patient risk stratification and clinical outcomes in internal cross-validation.

However, we acknowledge that the transcriptomic features, along with the reliance on nucleic acid and protein/modified protein assessments, may not be readily translatable to routine clinical diagnostics. Consequently, we focused on centering our predictions at the protein level and developed a predictive model based solely on protein data layers, which can be readily translated into immunohistochemical or ELISA assays. The 16 protein/phosphosite panel demonstrated only a slightly reduced AUC (0.818), while still effectively identifying high-risk patients and *ESR1* mutations (Fig. S16 and Supplementary Table 5). Additionally, both univariable and multivariable analyses for RFS and OS revealed significant associations for the proteome-centric predictive panel (multivariable RFS HR = 6.53, 95% CI: 2.10 to 22.25, *P* = 0.001; multivariable OS HR = 6.84, 95% CI: 1.87 to 24.98, *P* = 0.003; Supplementary Tables 8 and 9). These findings underscore the potential of integrated omics signatures to define disease states and predict patient prognosis, although further validation in independent cohorts will be required before clinical translation.

## Discussion

*ESR1* mutations are responsible for resistance to aromatase inhibitor-based endocrine therapies, and are frequently observed in ER-positive advanced breast cancers^39^. We and others have shown that these variants not only enhance estrogen receptor signaling, but also influence additional pathways, including mitotic activity or immune responses^8,10,13,16^. However, most of these findings are derived from preclinical models, which may not fully capture the complexity of clinical tumor biology. Furthermore, characterization of *ESR1* mutations in patient samples has often relied on pan-cancer datasets or metastatic cohorts, where heterogeneity in metastatic sites and prior treatment regimens can confound interpretation.

To address these limitations, we designed this study to evaluate the effects of *ESR1* mutations in a more controlled clinical context. Specifically, we analyzed therapy-naïve primary breast cancer samples from the SCAN-B cohort using proteogenomics, thereby minimizing confounding factors such as prior treatment exposure and elevated mutational burden. This strategy enabled unbiased assessment of the signaling changes associated with *ESR1* mutations in clinical specimens. Additionally, by integrating RNA sequencing and proteomic and phosphoproteomic data, we were able to define the relationships among gene expression, protein abundance and kinase activity, providing a more complete view of *ESR1* mutation signaling networks.

Clinical characterization of the cohort revealed a significant association between *ESR1* mutations and reduced relapse time and overall survival, consistent with previous reports^4^. Although *ESR1* mutations were predominantly observed in postmenopausal women, menopausal status itself did not significantly influence survival outcomes. Notably, adjuvant radiotherapy was associated with improved prognosis in Cox regression analyses, underscoring the protective role of radiotherapy in reducing recurrence and mortality rates^40^. No other clinical variables showed a significant association with survival, highlighting *ESR1* mutation status as the predominant risk factor in this cohort. Interestingly, MOFA analysis identified tumor grade and *ERBB2* amplification as the primary contributors to overall molecular variation, although neither was significantly linked to clinical outcome. Proteins associated with the most relevant latent factors were enriched for membrane-bound and extracellular matrix (ECM) components; this enrichment should be interpreted with caution. The use of AllPrep flow-throughs for proteomic extraction enriches for soluble proteins and may underrepresent membrane-associated and ECM proteins. While this approach has been shown to provide robust quantitative coverage of cytosolic and signaling proteins, it may be biased against structural and membrane-localized components.

Analysis of pathways associated with tumor grade showed a linear activation of several pathways, whereas *ESR1* mutations exerted a pronounced effect on only a subset. Tumor grade reflects key biological features, including the degree of tumor cell differentiation and proliferation^41,42^, and is a well-established determinant of patient outcome. Our results indicate that *ESR1* mutations tend to amplify certain tumor grade-associated pathways, but do not appear to act synergistically with them.

Interrogation of the mutational landscape using COSMIC revealed an enrichment of mutations commonly observed in ER-positive breast cancers, including alterations in genes such as PIK3CA or GATA3, consistent with established features of the luminal subtype^43^. Additionally, tumors harboring *ESR1* mutations exhibited a significantly higher TMB compared to control group samples. The elevated TMB was not associated with co-occurring mutations in other key driver genes or with clinical variables such as age at diagnosis. Instead, TMB correlated to transcriptional programs linked to cellular proliferation and DNA/RNA processing, suggesting a stochastic accumulation of genomic alterations driven by repeated cycles of accelerated cell division. Although mutation calling from RNAseq data is inherently more prone to false positives than DNA-based approaches, the application of stringent filtering criteria reduced the risk of overestimating per-sample TMB.

In breast cancer, the accumulation of mutations is often attributed to defined mutagenic processes such as defective DNA mismatch repair or the enzymatic activity of the APOBEC family of cytidine deaminases. APOBEC3B, in particular, has been implicated in facilitating DNA lesions through its interaction with ER, thereby contributing to chromatin remodeling and enhanced ER transcriptional activity^44^. Although the APOBEC protein family may potentiate the activity of mutant ER, we did not observe a clear association between APOBEC and *ESR1* mutations or provide evidence of increased APOBEC activity^45^. Collectively, these findings support a model in which *ESR1* mutations are associated with a proliferation-driven increase in TMB, likely mediated through downstream ER signaling pathways.

To further elucidate the molecular basis of increased tumor aggressiveness in breast cancers harboring *ESR1* mutations, we analyzed transcriptomic and proteomic data. This analysis revealed a consistent enrichment of cell cycle and metabolism pathways across both data layers, indicating activation of proliferative and biosynthetic processes in *ESR1* mutant tumors. In contrast, only modest enrichment was observed for canonical ER signaling pathways. Conversely, inflammation and immune-related signaling pathways were significantly downregulated in *ESR1* mutant tumors. These results are consistent with previous reports highlighting overactivation of proliferative signaling cascades in the context of mutated ER, including mTOR and CDKs. However, our results differ from earlier studies using preclinical models, where *ESR1* mutations were associated with increased interferon signaling^8,10,11,13^. This discrepancy may reflect differences in downstream signaling elicited by distinct *ESR1* mutations, as well as inter-model variability across metastatic breast cancer datasets^9^. Such heterogeneity may contribute to diverse metastatic phenotypes and underscores the context-dependent nature of *ESR1* mutant biology.

Consistent with previous observations in Y537S cell models^46^, we observed an enrichment of epithelial–mesenchymal transition (EMT) pathway at the proteome level in ER mutant tumors within the SCAN-B cohort. Notably, among the pathways enriched in *ESR1* mutant tumors, MTORC1 signaling was the only pathway significantly associated with shorter relapse time and overall survival. This association was consistently observed across all data layers, including the phosphoproteome, where KSEA indicated significant activation of RPS6Ks, which were found significantly activated alongside a reduced activity of inhibitory kinases such as PRKCA. These findings align with prior studies, demonstrating upregulation of MTORC1 signaling in both engineered and naturally occurring *ESR1* mutation models^8,13^, supporting a role for this pathway in promoting cancer progression. To further investigate this relationship, we re-analyzed our previously published multi-proteomic dataset^13^, which revealed an estrogen-dependent activation of the MTORC1 pathway in MCF7_Y537S_ cells. Under full media conditions and following 24-h estrogen stimulation, we identified an induction of a distinct protein sub-cluster enriched in metabolic proteins (e.g., ALDOA) and immune-related signaling components (e.g., GOT1), suggesting that these processes are regulated by estrogen signaling in the context of *ESR1* mutations.

Previous studies have explored the crosstalk between the estrogen and the mTOR pathways in breast cancer cells, mediated in part by S6K1 phosphorylation of the ER, which establishes a positive feedback loop that promotes proliferation^47–49^. On top of this, direct regulation of the MTORC1 pathway by estrogen has been shown in breast cancer models through both genomic (i.e., by modulation of downstream target genes) and non-genomic (i.e., via kinase signaling such as PI3K) activity of the ER^50^. Taking together the results in the SCAN-B cohort, where we observed *ESR1* mutations potentiating tumor grade-driven mechanisms, i.e., MTORC1, and the observations in MCF7 cell models where MTORC1 activation was dependent on estrogens and further increased in *ESR1* mutants, we argue that mutations in the ER exacerbate pre-existing mechanisms of metabolism, cell survival and proliferation in a context-dependent way. While we observed consistent MTORC1 signaling activation across multiple omics layers in both clinical samples and preclinical models, our analyses did not establish a direct regulatory hierarchy between *ESR1* mutations and MTORC1. Instead, the data support a model in which MTORC1 signaling represents a parallel proliferative program dependent on tumor grade that is exacerbated in tumor cells harboring *ESR1* mutations. While it is plausible that MTORC1 can be directly activated by estrogen and further potentiated under constitutive ER signaling, additional studies will be required to clarify the mechanistic relationship between *ESR1* mutants, ER activity, and MTORC1 signaling. Alternatively, both pathways may converge on shared proliferative networks driven by other determinants such as tumor grade.

Regardless of the precise mechanistic relationship, the interplay between the ER signaling and MTORC1 has possible therapeutic implications. Clinical trials such as BOLERO-2^51^ and TAMRAD^52^ have demonstrated that combining endocrine agents with MTORC1 inhibitors improves progression-free survival. More recently, results from the EMERALD trial have contributed to updated treatment recommendations for metastatic breast cancer, supporting the use of combined therapies, such as aromatase inhibitors or estrogen receptor degraders, with mTOR inhibition (e.g., everolimus)^53^. Despite these advances, modeling effective drug combinations in therapy-resistant settings remains challenging, especially in the context of *ESR1* mutations (reviewed in ref. ^54^), which are frequently observed in advanced disease and often exhibit intrinsic resistance to treatment. Multi-layered analysis of cancer mutations and their downstream effects is essential for accurately modeling signaling cross-talk mechanisms. Such approaches may ultimately guide the development of more effective, targeted treatment strategies.

In this study, we applied an integrative proteogenomic approach to elucidate the mechanisms by which *ESR1-*mutated tumors acquire a proliferative advantage, contributing to accelerated disease progression and reduced survival. This multi-layered analysis also enabled the identification of molecular signatures associated with highly aggressive breast cancers, independent of conventional clinical predictors. Notably, these signatures can identify patients at high risk of recurrence, regardless of *ESR1* mutation status in the study cohort.

While these signatures are promising, they currently represent a discovery step and require validation in larger, independent cohorts. The integrated proteogenomic model demonstrated the highest internal discriminatory performance, significantly exceeding the RNA and phosphoproteomic single-layer models, although its numerical improvement over the protein-centric model did not reach statistical significance. Moreover, the integrated signature has not yet been independently validated. The available external cohorts lack the matched transcriptomic, proteomic, and phosphoproteomic measurements required to evaluate the complete predictor, limiting our ability to assess its generalizability.

In particular, the extension of our observations at the proteomic levels remains limited by the lack of large-scale datasets. Thus, while these findings support the potential added value of integrating complementary molecular layers, independent validation in appropriately matched cohorts will be required before clinical generalizability can be established. The availability of comprehensive proteogenomic datasets would not only enhance statistical power for validation but also support the refinement of future predictive models using advances in computational approaches such as neural networks and digital twin frameworks^55^. Following extensive external validation via additional studies on datasets with matched multi-omic and clinical data, translation of these findings into clinically applicable assays such as immunohistochemistry or RNA in situ hybridization could facilitate their implementation in routine diagnostics.

## Conclusions

This study demonstrates the value of proteogenomics in dissecting the mechanisms underlying therapy resistance by integrating information across the central dogma, from DNA and RNA to protein and post-translational modifications. This approach provides a robust framework for advancing our understanding of cancer biology and for a discovery platform of clinically relevant multi-omic-based biomarkers.

## Methods

### SCAN-B primary tumor cohort

This study included a set of 27 breast cancer samples (female) expressing *ESR1* mutations for which residual material was available out of Swedish Cancerome Analysis Breast (SCAN-B; original set was described in refs. ^4,13^). A control set of 108 samples (i.e., four times the case group) was also selected based on matching ER status (staining positivity cutoff: 10%), PgR status (staining positivity cutoff: 10%), adjuvant endocrine treatment, ERBB2 amplification, and Luminal A/B subtype. All tumor samples were collected as part of the SCAN-B routine, where the specimen ischemia time, from excision from the patient to placement in RNAlater preservative solution and snap freezing, is kept to a minimum and is generally less than 1 h. Dataset median ischemic time was 45 min (IQR: 32–60 min), with ~70% of samples below 60 min. Eleven samples did not have a recorded ischemia time. Processing of frozen tumor specimens was performed using the Qiagen AllPrep method, and the extracted DNA, RNA, and flow-through portion that contains proteins were immediately frozen at −80 °C^22,56^.

Clinical parameters were defined according to the Swedish clinical routine. Age at diagnosis was employed for clinical analyses (range: 35 to 90). Proliferation marker Ki-67 status was defined according to local cutoffs. Tumor grading was performed according to the Nottingham scoring^57^. Sample dropout was based on the availability of material and data quality after proteomic data acquisition. Due to the retrospective nature of these samples, there was no attrition. A volume of 50–100 µL of Allprep flowthroughs from RNA extraction columns (Qiagen) was collected for this study. All samples were analyzed as biological replicates.

Inclusion of patients in the SCAN-B study, collection of clinical information and tumor specimens were conducted in accordance with the Declaration of Helsinki and with written informed consent prior to study enrollment. The use of residual material and RNAseq data was approved by the Regional Ethical Review Board of Lund with the following diary numbers: 2007/155, 2009/658, 2010/383, 2012/58, and 2013/459.

### RNA extraction, sequencing, and data processing

RNA extraction, quality control, and sequencing were performed as previously described^21,22,58^. Briefly, tumor specimens stored in RNAlater (Sigma), cut into pieces for RNA extraction using AllPrep RNA columns (Qiagen) following manufacturer protocols. RNA quality was evaluated by NanoDrop and BioAnalyzer (Agilent) or Caliper LabChip XT (PerkinElmer) capillary gel analysis. Strand-specific dUTP RNA-seq libraries were prepared, and sequenced to an average 38 million paired-end 75 bp reads on an Illumina HiSeq 2000 or NextSeq 500 instrument^4,13^.

Raw RNAseq data were processed as previously described. For expression analysis, reads were de-multiplexed, trimmed, and aligned to the human reference genome (GRCh38) with TopHat2 and gene expression in FPKM was estimated using Cufflinks^58^. For variant calling, reads were de-multiplexed, trimmed, and aligned to the human reference genome (GRCh38) with HISAT2. Variant calling was performed with VarDict-Java, and the resulting variants were annotated and filtered (see Brueffer et al, EMBO Mol Med for the detailed filtering methodology; calls are also available on: https://oncogenomics.bmc.lu.se/MutationExplorer)^21^. For this study, the calls were further filtered for COSMIC, Sanger 100 breast cancer exome study, and TCGA gene variants to minimize false discoveries.

### Cell culture and lysis

MCF-7 (RRID: CVCL_0031), MDA-MB-468 (RRID: CVCL_0419) and SKBr-3 (RRID: CVCL_A2FE) were kindly gifted by the laboratory of Prof. John W Martens (Erasmus Medical Center, Rotterdam, The Netherlands) and cells were cultured in Roswell Park Memorial Institute medium (RPMI supplemented with GlutaMAX™; Thermo-Fisher) containing 10% fetal bovine serum (FBS; Gibco) and supplemented with antibiotics (100 units/mL penicillin G; 0.1 mg/mL streptomycin; 45 µg/mL Gentamycin; Thermo-Fisher). Cells were harvested at 80–90% confluence and lysed in RIPA buffer with protease inhibitors (Halt Protease Inhibitor and 5 mM EDTA; Thermo-Fisher). Cells were lysed via sonication on a Bioruptor instrument (20 cycles: 30 s ON, 30 s OFF; RRID: SCR_023470, Bioruptor Plus, Diagenode), and centrifuged at 14,000 × *g* for 20 min at 4 °C. Protein supernatants were collected, and concentration was measured with the bicinchoninic acid assay (Thermo-Fisher), according to the manufacturer’s instructions. Around 1 mg of total protein lysate was employed for downstream protein digestion.

### Sample preparation for mass spectrometry

Protein mixtures (AllPrep flowthroughs and cell lysates) were precipitated in ice-cold (−20  °C) methanol.

Protein pellets of samples for spectral library generation (cell lines and pooled AllPrep flowthroughs; 1 mg approximate protein content) were resuspended in 7 M Urea in 100 mM ammonium bicarbonate buffer (AMBIC), then reduced by adding a tris(2-carboxyethyl)phosphine in 100 mM AMBIC (final concentration: 5 mM) and incubating them for 1 h at 37  °C. Alkylation was performed by adding iodoacetamide in AMBIC buffer (final concentration: 10 mM) and incubation for 30 min in the dark at RT. Urea was then diluted to 1 M with AMBIC, and Trypsin/Lys-C (Promega) was added in a 1:100 w/w ratio (enzyme/protein) to the samples, which were incubated at 37 °C for 16 h under agitation. A solution of 10% formic acid (FA) was then added to acidify the peptide mixtures. Samples were centrifuged at 14,000 × *g* for 20 min at 4 °C, supernatants were collected, and peptide mixtures were desalted using OASIS HLB columns according to previously published methods^59,60^. Briefly, Oasis HLB cartridges (Waters) were loaded onto a vacuum manifold, primed with 100% V/V methanol, conditioned with 80% V/V acetonitrile (ACN) in 0.1% V/V FA solution, and equilibrated with 0.1% V/V FA in high-grade water. Samples were loaded, washed with 0.1% V/V FA in water, and eluted with 45% V/V ACN in 0.1% V/V FA solution. Eluates were dried in a SpeedVac until further processing.

SCAN-B sample set protein pellets were then dried and re-suspended in 4 M Urea in 100 mM tri-ethyl-ammonium bicarbonate (TEAB) buffer, and loaded on 96-well plates for downstream digestion according to previously published protocols on the AssayMAP Bravo^61^. Briefly, protein mixtures were reduced by adding 10 mM of dithiothreitol (final concentration) and incubating them for 1 h at 37  °C. Alkylation was performed by adding 20 mM iodoacetamide (final concentration) and incubating mixtures for 30 min in the dark at RT. Urea was then diluted to 1 M using TEAB, and Trypsin/Lys-C (Promega) was added in a 1:100 w/w ratio (enzyme/protein). Samples were then incubated overnight at 37  °C. Digestion was stopped by the addition of 20 µL of 10% trifluoroacetic acid (TFA) solution, incubation at 37  °C for 30 min, and peptide mixtures were then desalted using the Peptide Clean-up v2.0 protocol. Briefly, C18 cartridges (Agilent) were primed with 100 μL of 90% ACN, equilibrated with 70 μL of 0.1% TFA at 10 μL/min, and loaded with peptide mixtures. Internal cartridge wash and cup wash with 0.1% TFA at 10 μL/min were performed. Peptides were eluted with 30 μL 80% ACN with 0.1% TFA. A pre-existing volume of 50 μL containing 50% ACN with 0.1% TFA was present in the collection plate upon elution. About 10% of the eluate volumes were dried and stored for downstream proteome MS analysis, while the remainder was subjected to phosphopeptide enrichment.

### High-pH sample fractionation

Peptide mixtures from breast cancer cell lines and pooled AllPrep flowthroughs were fractionated using a Waters XBridge BEH130 C18 (3.5 μm, 2.1 × 150 mm column; Waters) coupled to an Ultimate 3000 system (Thermo). Equilibration and peptide elution buffers were composed of 20 mM ammonium formate in high-grade water and 10 mM ammonium formate in 90% V/V ACN, respectively. Gradient was run as follows (constant flow of 200 μL/min): 3 min at 0% B, 35% B in 94 min, 80% B in 1 min, and 10 min at 80% B at a column oven temperature of 30  °C. Fractions were collected for the first 97 min using a Thermo Scientific fraction collector, for a total of 96 fractions. Of these, 90% of the eluted volume was concatenated into 12 fractions for downstream phosphopeptide enrichment, while the remainder was concatenated into 24 fractions for proteome analysis. The concatenation scheme for the eluates’ 96-well plates was: fraction 1 (well A1, C1, E1, G1), fraction 2 (well A2, C2, E2, G2), etc. Concatenated fractions were dried and frozen until downstream processing or MS analysis.

### Phosphopeptide enrichment

Samples for spectral library preparation (cell line and AllPrep flowthrough fractions) and clinical cohort analysis (SCAN-B AllPrep flowthroughs) were processed according to a previously published automated phosphopeptide enrichment protocol^62^. Briefly, peptide mixtures out of high-pH fractionation or directly out of C18 desalting were loaded on the AssayMAP Bravo platform. The Fe(III)-NTA cartridges employed here were primed with 50% V/V ACN, 0.1% V/V trifluoroacetic acid (TFA), equilibrated with loading buffer (i.e., 80% V/V ACN and 0.1% V/V TFA in high-grade water). Peptide mixtures were then loaded on phosphoenrichment cartridges, washed with loading buffer, and eluted with 5% V/V ammonia solution into a 50% V/V FA solution (eluates’ plate). Samples were then dried and stored at -80 °C until MS analysis.

### Mass spectrometry analysis

Peptide mixtures were loaded onto Evosep tips (Evosep Biosystems) according to manufacturer instructions. An approximate amount of 400 ng of digested peptides was loaded on each tip. Loaded peptides were separated on a nanoflow reversed-phase chromatography Evosep One LC system (Evosep Biosystems) coupled to a timsTOF HT ion mobility MS (Bruker). The LC was equipped with a 15 cm × 150 µm × 1.5 μm PepSep C18 column (Bruker). Separation was performed using the built-in 30-sample-per-day method (44 min gradient length).

For library generation (proteome and phosphoproteome), the MS was run in standard DDA Parallel Accumulation Serial Fragmentation (PASEF) mode, with ten PASEF scans every acquisition cycle. For the SCAN-B clinical sample set, the MS was run in DIA PASEF mode, which was in turn optimized with py_diAID^63^ based on generated proteome and phosphoproteome libraries, respectively. For both methods, precursors with *a* + 1 charge were ignored, and the target intensity was set to 20,000, with a dynamic exclusion window set to 0.4 min. Isolation width was set to 2 at 700 Th and 3 at 800 Th.

### Proteomic file processing

DDA PASEF MS data were processed through Fragpipe (v22.0; https://fragpipe.nesvilab.org/) with the built-in LFQ-MBR method and additionally activating the spectral library generation plug-in. For the generation of the phosphoproteome spectral library, the STY option was enabled in the modification tab of the MSFragger panel.

DIA PASEF MS data was processed using DIA-NN (v1.9.0)^64^ using default settings, with the exception of match between runs (enabled) and using the previously generated and above-described spectral libraries. The STY option was enabled in the modification panel for the phosphoproteome data search.

A FASTA file for database search employed for DDA and DIA searches was downloaded from Uniprot (RRID: SCR_002380; www.uniprot.org), selecting only the Swissprot subset (version 2024.01.15). Protein true identifications were determined using an FDR threshold of 1%.

### Data analysis

RNAseq data expressed as FPKM were mean-scaled and centered across samples and genes prior to further processing. Variant calling tables were filtered to include only frameshift, missense, nonsense, and splicing variants. Tables were then dichotomized as 0 (non-mutated) and 1 (mutated) for further analysis.

Phosphosite table (90% probability cutoff; phosphoproteome), precursor, and protein group (proteome) tables out of DIA-NN were employed for downstream analysis. For all data layers, input tables were filtered for missing data (MD filter transcriptome: 30%, proteome: 40%; phosphoproteome: 80%), log2-transformed, and mean-centered across samples and features (genes, proteins, phosphosites). For the proteomic and phosphoproteomic datasets, batch-effect correction and quantile normalization were performed with limma (RRID: SCR_010943; v3.46.0) in R (v4.0.5).

MOFA analysis was run with a random seed for 100 iterations to check for robustness and model convergence. As the variances explained by each data layer across the 100 iterated models were identical, we assumed MOFA results did not depend on stochastic initialization. MOFA was run with default settings and variance decomposed into 15 factors. Subsampling analysis was also performed in 5 iterations of randomized sampling, picking 80% of the total samples at each iteration. Correlation with the full MOFA model was then performed for factor 1.

Pathway analyses were performed with Gene Set Enrichment Analysis (GSEA; v4.1.0)^65^ using the Hallmarks and Reactome databases (RRID: SCR_003485; from MSigdDB, v2024.01). GSEA settings were as follows: gene set permutation type, classic scoring method and *t*-test two-group test metric. False discovery rate (FDR) cutoff for significant pathways was set to 0.25. Reactome pathways were plotted using the Enrichment Map plug-in in Cytoscape (RRID: SCR_003032; v3.8.2). Sample-wise pathway (Hallmarks subtypes and Kinase-substrate out of the KSEA database) scores were calculated using GSVA^29^ (v1.38.2) in R. Phosphoproteomic data were analyzed by Kinase-Substrate Enrichment Analysis (KSEA, v0.99.0)^34^ in R. Here, Network KIN terms were allowed with a cutoff of 5. Enriched Kinases were selected based on *P*-value (<0.05) and number of substrates (>2). Metascape^26^ analyses were performed with the online tool (RRID:SCR_016620; https://metascape.org/) employing gene names or Uniprot IDs as input. MOFA analysis and statistical tests (i.e., Fisher, *χ*^2^, Wilcoxon, one-way ANOVA, Kruskal-Wallis, Log-rank, *t*-test, and Cox regression) were all performed in R (v4.5.0).

Previously published proteomic and phosphoproteomic datasets (from proteomeXchange ID PXD046446; https://www.ebi.ac.uk/pride/archive/projects/PXD046446) were subjected to the same pre-processing as the other expression tables described here. The GSE147745 (https://www.ncbi.nlm.nih.gov/geo/query/acc.cgi?acc=GSM4445210) RNAseq dataset was downloaded from GEO, and raw counts were analyzed by DESeQ2 in R (RRID: SCR_015687; v1.49.3). The external datasets on engineered *ESR1* mutant cell lines were analyzed by DEqMS (RRID: SCR_025605; proteomic) and DeSeq2 (RNAseq). The top and bottom (*n* = 150 + 150 by fold change) significant features were queried in clue.io^35^ against the L1000 gene expression database. Output was filtered to only contain the top 10 (by connectivity score) drug perturbagens (i.e., MOA_CLASS) for each query (i.e., each *ESR1* mutant over *wt*).

For predictive ROC models, feature selection and model training were performed using the SCAN-B dataset. To minimize overfitting, coefficients were derived independently from differential expression statistics and Cox regression significance. Nevertheless, because model development and evaluation were performed within the same cohort, predictive performance estimates should be interpreted cautiously.

We selected transcripts, proteins, and phosphosites that passed the following threshold: Cox regression (median cutoff for two groups comparison; tie method: Breslow) *P*-value < 0.05 for RFS and OS, absolute Log fold change from differential expression (transcriptomics: DeSeq2, proteomics: DEqMS; phosphoproteomics: limma) >5× Quartile-IV Log fold change. Predictive ROC models were generated in R (Rocit v2.1.2). Scores for each feature (transcript, protein, or phosphosite) were calculated by multiplying scaled intensities by the test coefficient (transcriptomic: DeSeq2 “stat”; proteomic: DEqMS “t”; phosphoproteomic: limma “t”). Scores for each feature were summed for each sample and *Z*-scored across the dataset. Predictions were based on the *Z*-score resulting from each combination (i.e., single data layer or combinations). Youden index (i.e., max specificity and sensitivity) was selected as the cutoff point for defining high and low risk groups.

Model robustness and predictive performance of predefined signatures at the single-data-layer and integrated-layer levels were assessed using leave-one-out cross-validation (LOOCV). In each iteration, the predictive model was trained on all samples except one, and the left-out sample was used for prediction. Paired De Long’s tests were performed to compare the AUCs of the different ROC models, and *P*-values were corrected using Holm’s method.

For survival analyses involving GSVA scores, median values were employed as cutoffs to define high and low-expressing samples. While this approach provides an unbiased threshold in the absence of predefined cutoffs, we acknowledge that different thresholds may be more accurate in linking survival and pathway activation.

For the METABRIC, TCGA, and SweBCG91RT breast cancer cohorts, scaled and centered gene expression data were used to calculate the signature’s sample-wise score. High-risk patients were defined here based on fast recurrence (i.e., recurrence event within 1 year) due to a lack of *ESR1* mutation status. Fisher and Kaplan-Meier plots were employed to measure the performance of each predictive model's cutoff.

## Supplementary information

Transparent Peer Review file

Supplementary Information

Description of Additional Supplementary Files

Supplemental Data 1

Supplemental Data 2

Supplemental Data 3

## Data Availability

The source data for Figures is presented in Supplementary Data 3 and is accessible on Figshare via https://doi.org/10.6084/m9.figshare.33277794. RNA-seq raw data contain potentially identifiable human genetic information and are therefore regarded as personal information under Swedish law and cannot be made publicly available. RNAseq-derived gene expression and SNV tables, proteomic and phosphoproteomic tables and clinical metadata for all samples used in this study are also available on Figshare under https://doi.org/10.6084/m9.figshare.33277794. Requests for access to the raw RNA-seq data should be directed to Lao H. Saal (lao.saal@med.lu.se). Access may be granted where permitted by applicable ethical, legal, and data-protection requirements and subject to a data access agreement governing the use of the data. Proteomic and phosphoproteomic data and related sample descriptor files have been deposited to the ProteomeXchange Consortium via the PRIDE (RRID: SCR_012052) partner repository^66^ with dataset identifiers PXD060347 (library samples DDA data and SCAN-B samples DIA data; https://www.ebi.ac.uk/pride/archive/projects/PXD060347). RNAseq data and full clinical information are available on Mendeley via https://doi.org/10.17632/yzxtxn4nmd.4.
