# Supplementary material for: ESR1 mutations are associated with enhanced MTORC1 signaling in a context-dependent manner
Source: Commun Biol. 2026 Oct 7;9:1304. doi: 10.1038/s42003-026-11096-w (PMC13645737; doi:10.1038/s42003-026-11096-w)
Supplement: Supplementary file 1 [file 42003_2026_11096_MOESM1_ESM.pdf]

nature portfolio

Peer Review File

ESR1 mutations are associated with enhanced MTORC1 signaling in a context-dependent manner

Corresponding Author: Dr Tommaso De Marchi

This file contains all reviewer reports in order by version, followed by all author rebuttals in order by version.

Version 0:

Reviewer comments:

Reviewer #1

(Remarks to the Author)
This manuscript presents an analysis of primary breast cancers harbouring ESR1 mutations, integrating transcriptomic, proteomic, and phosphoproteomic data. The consistent association between ESR1 mutations, proliferative programs, and adverse outcome is reported by the authors alongside the identification of MTORC1 signalling as a recurrently enriched pathway across data layers. Several central claims rely on correlative evidence and are framed more strongly than the data support. These issues do not invalidate the study, but they require clearer framing, explicit acknowledgment of limitations, and moderation of causal language.
Major points
1.The central conceptual claim is that MTORC1 acts as a co-regulator of tumour aggressiveness in ESR1-mutant cancers. The data demonstrate that MTORC1 pathway signatures are consistently enriched in ESR1-mutant tumours and that higher "MTORC1 activity" associates with shorter relapse-free and overall survival. Kinase enrichment analysis supports downstream pathway activation, and cell line data show estrogen-conditioned MTORC1 elevation.
There is no direct mechanistic or regulatory interaction between ESR1 mutants and MTORC1 demonstrated. All evidence remains at the level of pathway enrichment, kinase-substrate inference, and correlative survival analysis. As currently written, the term co-regulator implies functional interdependence or regulatory hierarchy. The data instead support MTORC1 as a parallel, consistently activated growth program associated with ESR1-mutant disease. This distinction matters because it underpins the title, abstract, and mechanistic interpretation.
2. Proteins and phosphopeptides were derived from Allprep RNA extraction flowthroughs. While somewhat unusual based on approaches we have utilised, this approach potentially introduces systematic pre-analytical biases that are not sufficiently discussed.
Extraction method strongly influences protein recovery, particularly for membrane, ECM, and cytoskeletal proteins. Flowthrough-based inputs are likely enriched for soluble proteins and depleted for membrane-associated and ECM components. This is particularly relevant here because ECM, EMT, and microenvironment-related pathways feature prominently in the results and discussion. Without explicit acknowledgment, readers may overinterpret pathway-level differences as purely biological rather than partly shaped by input bias.
3. While the authors correctly note the limited availability of large-scale breast cancer proteomic datasets for validation, the current framing overstates clinical readiness. The conclusions should more clearly distinguish discovery from validation and acknowledge that proteome-level generalization remains to be established. Moderating the translational claims would bring the manuscript in line with analytical validation standards without requiring additional experiments.
Minor points and clarity issues
-The MOFA analysis shows that ESR1 mutation status does not contribute strongly to the dominant axes of variance. This is an important observation but is somewhat under-integrated into the interpretation. The manuscript should more explicitly reconcile this with later claims of ESR1-driven signalling programs.
-Survival analyses use median splits of GSVA scores. While acceptable, sensitivity to cut-off choice is not discussed. A brief acknowledgment would improve robustness.
-The discussion occasionally conflates estrogen dependence, ESR1 mutation effects, and MTORC1 activation. These relationships should be described with greater precision to avoid implying causality where none is shown.
-The predictive models include relatively small numbers of proteomic features. The risk of overfitting should be acknowledged more explicitly, especially given the cohort size.

Reviewer #2

(Remarks to the Author)
This study extends prior preclinical findings by validating them in patient tumor samples rather than relying solely on cell-line models. It identifies increased overall mutational burden as a feature of ESR1-mutant disease and narrows the focus to MTORC1 activation, including a proposed estrogen-dependent component that may contribute to tumor aggressiveness. The analysis links MTORC1 signaling to higher tumor grade and recurrence risk and introduces a proteogenomic signature intended for patient selection and risk stratification, highlighting its potential clinical relevance.
There are few major concerns for this study:
Unclear Tissue Processing and Preservation
The manuscript does not specify whether samples were fresh frozen, FFPE, or otherwise preserved. Since phosphoproteomic signals are highly sensitive to handling and fixation, these pre-analytical details are critical for interpreting MTORC1 activation results.
Lack of Omics Quality Control Reporting
Essential QC metrics for RNA-seq, proteomics, and phosphoproteomics are not presented. Information on sequencing quality, protein/phosphosite identification confidence, normalization, batch correction, and reproducibility is necessary to assess data robustness.
Potential Circular Validation (Figure 5)
Figure 5 appears to validate MTORC1 elevation using a dataset that may overlap with the group's prior preclinical study. If not fully independent, this risks circular validation rather than true confirmation. The authors should clarify dataset independence and how this analysis represents external validation.

Minor concern:
Cis and trans association across omes should be reported

Reviewer #3

(Remarks to the Author)
This study presents a multi-omics analysis of ESR1-mutant breast cancers integrating transcriptomic, proteomic and phosphoproteomic data from the SCAN-B cohort. The study provides evidence that MTORC1 signaling is enriched in ESR1-mutant tumors and may contribute to tumor aggressiveness. While this link is not entirely novel, it is expanded and further characterized in the current study, which also provides relevant molecular signatures associated with clinical outcome.
1. The current study confirms and extends the ER–mTOR association using an integrated transcriptomic, proteomic and phosphoproteomic approach in a clinically relevant cohort. However, the extent to which the findings represent fundamentally new biological insights versus a multi-layered confirmation of previously reported mechanisms should be better clarified and, if possible, expanded. The study may benefit from assessing functional and statistical interactions within the integrated dataset.
2. The study also proposes predictive signatures of clinical outcome. However, it appears that feature selection and model development were largely performed within the same dataset used for evaluation. This raises concerns about potential overfitting and inflated predictive performance. Although validation in external transcriptomic datasets is presented, the full multi-omic predictor cannot be validated due to the lack of comparable datasets. The authors may clarify the training and validation strategy used and provide more details regarding cross-validation procedures and model stability.
3. The observation of an association with tumor mutational burden (TMB) is interesting but should be interpreted cautiously. It is not entirely clear whether the increased mutation burden observed here reflects a direct biological consequence of ESR1 mutations, underlying tumor heterogeneity, or other confounding factors. In addition, mutational burden is inferred from RNA-seq data, which may introduce biases. Comparison with other cohorts and data types would strengthen this observation.
4. Although the study generates a rich multi-omics dataset, the predictive models appear to rely predominantly on transcriptomic features. This raises the question of whether the predictive performance is largely driven by the transcriptomic component rather than by true integration across data layers. The authors should clarify how features from different omics layers were selected and integrated into the predictive models, and whether models based on each individual layer were systematically compared to assess the added value of the proteomic and phosphoproteomic data generated in this study. In addition, describing cross-omic interactions could add a novel layer of interpretation linking functional connections and clinical outcomes.
5. The study applies Multi-Omics Factor Analysis (MOFA) to identify major sources of variation across the transcriptomic, proteomic and phosphoproteomic layers. While this is an appropriate framework, it should be noted that the number of ESR1-mutant tumors included in the study is relatively limited. Given the high dimensionality of the dataset and the relatively small number of mutant samples, it would be helpful for the authors to further discuss the robustness of the factor decomposition and the stability of the identified factors. Additional information on how sensitive the MOFA results are to sample composition, or whether robustness analyses were performed, would strengthen confidence into the analyses.

Version 1:

Reviewer comments:

Reviewer #1

(Remarks to the Author)
Thank you for the detailed revision. The additional analyses and revised discussion have improved the manuscript and the authors now address several of my previous concerns.
I still think the revised title expresses language stronger than the data suggests and implies a functional and causal relationship. Secondly, I think the reporting of phosphoproteomic handling needs to be improved (time from resection to lysis/freezing, use of phosphotase and protease inhibitors, statement on any difference in processing time between samples).

Reviewer #2

(Remarks to the Author)
The authors have addressed the all my major concerns

Reviewer #3

(Remarks to the Author)
The authors have performed substantial additional analyses and improved the framing of the study, enhancing its overall presentation and interpretability.

Nevertheless, in my opinion, the claim that the integrated proteogenomic model provides superior predictive performance remains insufficiently supported. Although the integrated and protein-centric models show higher LOOCV AUC values than the transcriptomic model, the confidence intervals overlap, no formal comparison of predictive performance is presented, and external validation remains restricted to the transcriptomic signature.

As acknowledged by the authors, I recognize that such validation is challenging given the nature and breadth of the required datasets. It also remains unclear whether feature selection, scaling, and cutoff determination were repeated within each cross-validation fold. The results therefore support a numerical improvement in internal performance, but generalizability cannot yet be claimed, particularly as the analyses are limited to a single dataset. Thus, while the results and conclusions are potentially valuable, further studies will be required to extend and independently confirm them, and the corresponding language should be moderated accordingly.

Open Access This Peer Review File is licensed under a Creative Commons Attribution 4.0 International License, which permits use, sharing, adaptation, distribution and reproduction in any medium or format, as long as you give appropriate credit to the original author(s) and the source, provide a link to the Creative Commons license, and indicate if changes were made.
In cases where reviewers are anonymous, credit should be given to 'Anonymous Referee' and the source.
The images or other third party material in this Peer Review File are included in the article’s Creative Commons license, unless indicated otherwise in a credit line to the material. If material is not included in the article’s Creative Commons license and your intended use is not permitted by statutory regulation or exceeds the permitted use, you will need to obtain permission directly from the copyright holder.

To view a copy of this license, visit https://creativecommons.org/licenses/by/4.0/

Reviewer #1 (Remarks to the Author):

This manuscript presents an analysis of primary breast cancers harbouring ESR1 mutations, integrating transcriptomic, proteomic, and phosphoproteomic data. The consistent association between ESR1 mutations, proliferative programs, and adverse outcome is reported by the authors alongside the identification of MTORC1 signalling as a recurrently enriched pathway across data layers. Several central claims rely on correlative evidence and are framed more strongly than the data support. These issues do not invalidate the study, but they require clearer framing, explicit acknowledgment of limitations, and moderation of causal language.

Major points

1. The central conceptual claim is that MTORC1 acts as a co-regulator of tumour aggressiveness in ESR1-mutant cancers. The data demonstrate that MTORC1 pathway signatures are consistently enriched in ESR1-mutant tumours and that higher "MTORC1 activity" associates with shorter relapse-free and overall survival. Kinase enrichment analysis supports downstream pathway activation, and cell line data show estrogen-conditioned MTORC1 elevation.

There is no direct mechanistic or regulatory interaction between ESR1 mutants and MTORC1 demonstrated. All evidence remains at the level of pathway enrichment, kinase-substrate inference, and correlative survival analysis. As currently written, the term co-regulator implies functional interdependence or regulatory hierarchy. The data instead support MTORC1 as a parallel, consistently activated growth program associated with ESR1-mutant disease. This distinction matters because it underpins the title, abstract, and mechanistic interpretation.

We thank the reviewer for this comment and agree that our analysis reveals MTORC1 to be operating at the same time as *ESR1* mutations, but not necessarily as a functionally dependent mechanism. We have expanded this analysis, starting with our MOFA results and evaluating the relationship with the major determinant of dataset variation, tumor grade. Our analyses here have shown that several Hallmarks pathways are linearly connected with grade in either a direct or inverse relationship. For these pathways, we also evaluated additive and interaction effects with *ESR1* mutations, the first indicating an effect on UV response (negative relationship with grade) and MTORC1 signaling (positive relationship with grade), while the other indicating a parallel and independent activation due to the non-significance p-values. In addition to this, we also evaluated the correlation between ER transcript abundance, the ER and MTORC1 signaling pathways (GSVA scores), which indicated the absence of a direct relationship with ER gene levels and only a partial one with ER downstream effects (signaling). Altogether, these data point out that *ESR1* mutations operate on top of major determinants of tumor biology and may exacerbate their biological effects and patient outcome.

In the light of these results, we have made changes through all sections of the manuscript:

- The title of the manuscript was changed to "*ESR1* mutations enhance MTORC1 signaling in a context-dependent manner to promote tumor aggressiveness"

- Abstract. We edited our text to reflect the convergence of MTORC1 and *ESR1* pathways in contributing to worse prognosis rather than the causal bond between them (p4, lines 36-40).
- Results. We have edited the text on: p11, lines 185-7; p12, lines 203-13 and lines 216-7; p16, lines 305-9 and 311; p17, lines 318-33; p18, lines 359-64; p18, line 370.
- Discussion. We have text on: p23, lines 455-60; p25, lines 507-9; p25-6, lines 512-28.

We have added Figure S5 and modified main Figure 4 (below) to better define the connection between MTORC1, grade and *ESR1* mutations. We have also edited the text and clarified our points and our language through the whole manuscript.

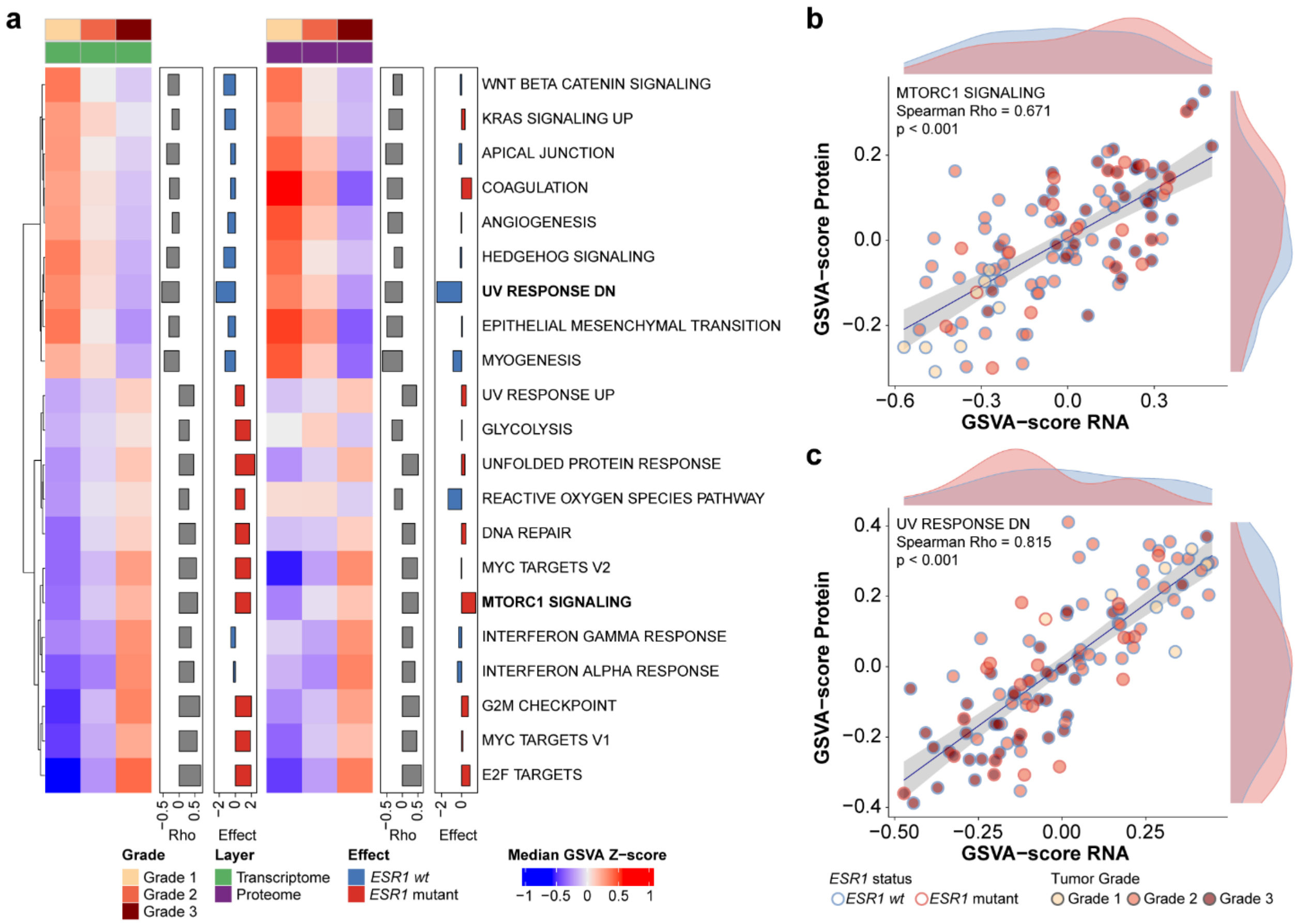

Figure S5. Pathways regulated by tumor grade and impact of *ESR1* mutations.

Hallmarks RNA and protein GSVA scores were summarized (metric: median) and evaluated for linearity association. Pathways overlapping between RNA and protein data layers were plotted in a heatmap (panel A). Bar charts to the right of each heatmap depict Spearman Rho between tumor grade and GSVA scores (left) and linear model statistic to measure ESR1 mutation effect on each pathway (right). Panels B and C show scatterplots of RNA and protein GSVA scores for pathways where ESR1 mutations had an effect (linear model |statistic| > 1) both at the RNA and protein level.

Acronyms: GSVA: Gene Set Variation Analysis.

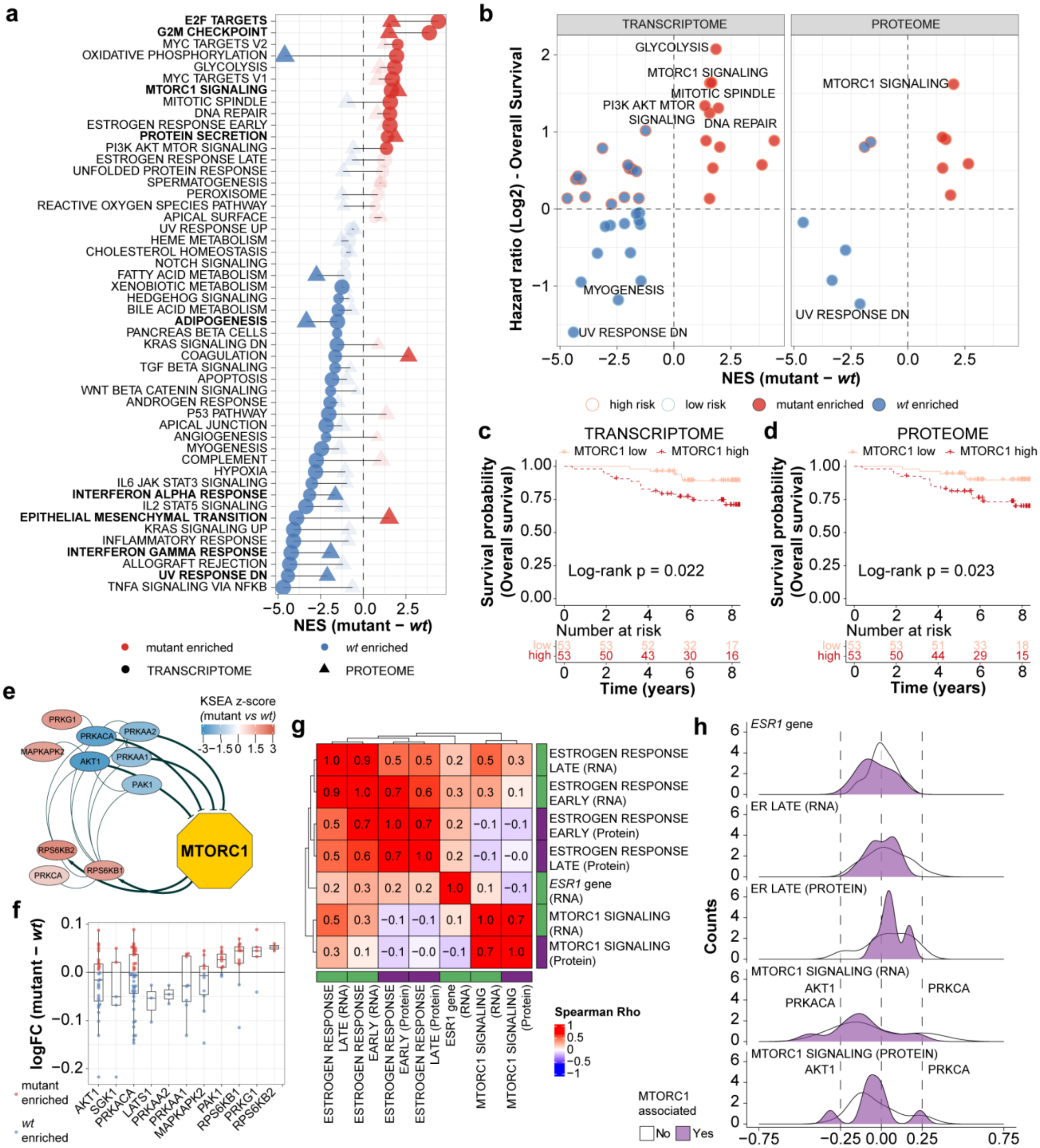

Fig 4. Integrated analysis shows ESR1 mutations in primary breast cancers are geared towards proliferation and metabolism.

A normalized gene set enrichment score (NES) was calculated between ESR1 mutants and wt tumors for the transcriptomic and the proteomic data layers. The NES score for each gene set was then plotted for both proteomics (triangles) and transcriptomics (circles). Non-significant gene sets are opaque for clarity. Pathways in bold represent significant enrichment in both data layers (panel A).We calculated sample-wise Hallmarks pathway scores using GSVA and performed Cox regression analysis for Relapse-free Survival (RFS) for each pathway (transcriptomics and

proteomics). Cox regression derived Hazard Ratios (HR) were log transformed and plotted against the NES of each pathway out of panel A in scatter plots. Pathways enriched in GSEA analyses and associated with high/low risk of relapse located in the first and third quadrant of the plots (panel B). We extracted the GSVA scores for the pathway that was consistently enriched in ESR1 mutants in both the transcriptome and protoeme layers and significantly associated to poor prognosis, split the patient groups based on median GSVA score and performed KM analysis (panels C and D). We performed KSEA analysis on the phosphoproteomic data to derive kinases enriched in ESR1 mutants and controls. Cytoscape was employed to generate enrichment an map depicting KSEA results for the MTORC1 pathway associated kinases (panel E). Phosphopeptide intensities of enriched kinase (from panel E) substrates were then plotted in box plots (panel F). GSVA scores for the transcriptome and proteome were extracted along with ESR1 transcript abundance and a correlation matrix (method: Spearman) was computed. Hierarchical clustering was then performed and plotted as a heatmap (panel G). Phosphosites linked to MTORC1 associated kinases were assembled into GSVA scores (see Methods) and correlated to ESR1 transcript abundance (top), MTORC1 signaling RNA-based (middle) and protein based (bottom) GSVA scores (panel H). Top 5 positively and negatively correlating kinases are shown in middle and bottom panels.

Acronyms: GSEA: Gene Set Enrichment Analysis; GSVA: Gene Set Variation Analysis; KM: Kaplan-Meier; KSEA: Kinase-Substrate Enrichment Analysis; NES: Normalized Enrichment Score; OS: Overall Survival; RFS: Relapse-free survival.

2. Proteins and phosphopeptides were derived from Allprep RNA extraction flowthroughs. While somewhat unusual based on approaches we have utilised, this approach potentially introduces systematic pre-analytical biases that are not sufficiently discussed.

Extraction method strongly influences protein recovery, particularly for membrane, ECM, and cytoskeletal proteins. Flowthrough-based inputs are likely enriched for soluble proteins and depleted for membrane-associated and ECM components. This is particularly relevant here because ECM, EMT, and microenvironment-related pathways feature prominently in the results and discussion. Without explicit acknowledgment, readers may overinterpret pathway-level differences as purely biological rather than partly shaped by input bias.

We agree with the reviewer on the use of caution in inferring enrichments of particular subsets of proteins (e.g. ECM) out of Allprep protein extractions. We have actually analyzed our previously published proteomic datasets (PMID: 33855848; dataset: PXD018830), where we extracted proteins from the same tissues by tissue homogenization and Allprep. GSEA analysis showed an enrichment of coagulation, EMT and myogenesis gene sets (Hallmarks database) in homogenate over Allprep samples (see below).

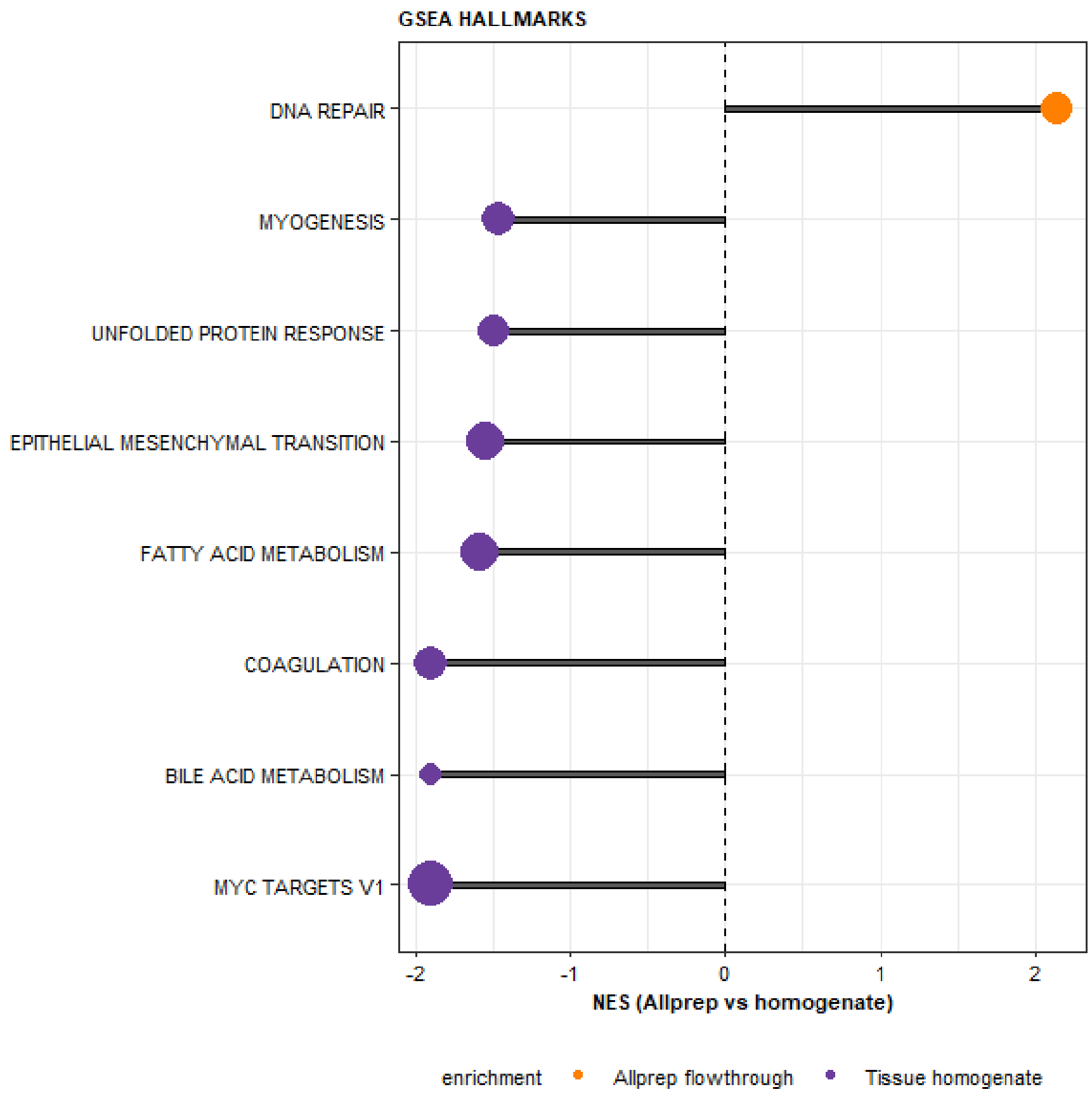

We have not mentioned this dataset as it was generated and published with other aims than homogenate-Allprep comparison and this analysis was not incorporated into the final version of the manuscript, but we have added this point in the Results (p8, lines 121-5) and Discussion sections (p23, lines 449-54).

3. While the authors correctly note the limited availability of large-scale breast cancer proteomic datasets for validation, the current framing overstates clinical readiness. The conclusions should more clearly distinguish discovery from validation and acknowledge that proteome-level generalization remains to be established. Moderating the translational claims would bring the manuscript in line with analytical validation standards without requiring additional experiments.

We agree with the reviewer on this point and have edited our text accordingly (Abstract: p4, lines 44-5; Results: p21, lines 421-2; Discussion: p27, lines 547-52 and lines 557-9).

Minor points and clarity issues

-*The MOFA analysis shows that ESR1 mutation status does not contribute strongly to the dominant axes of variance. This is an important observation but is somewhat under-integrated into the interpretation. The manuscript should more explicitly reconcile this with later claims of ESR1-driven signalling programs.*

We thank the reviewer for this observation. MOFA analyses showed that *ESR1* mutations seem to exist on top of major pathways, but do not re-write luminal breast cancer biology. In this light, these mutations might work in exacerbating already existing mechanisms of tumor growth and aggressiveness. We have added and expanded this point in our Results (p11, lines 185-7; p12, line 216-7; p16, lines 305-9 and line 311; p17 318-33; p18, lines 359-64) and Discussion (p23, lines 455-60; p25, lines 507-9) sections.

-*Survival analyses use median splits of GSVA scores. While acceptable, sensitivity to cut-off choice is not discussed. A brief acknowledgment would improve robustness.*

We have added this in our Methods section (p36, lines 757-60).

-*The discussion occasionally conflates estrogen dependence, ESR1 mutation effects, and MTORC1 activation. These relationships should be described with greater precision to avoid implying causality where none is shown.*

We agree with this comment and we have edited the text throughout the entire manuscript.

-*The predictive models include relatively small numbers of proteomic features. The risk of overfitting should be acknowledged more explicitly, especially given the cohort size.*

We thank the reviewer for this comment and apologized for omitting our cross-validation analyses. We have added these and the proper text across the sections of the manuscript (Table S7; Results: p20, lines 396-8; Methods: p35, lines 739-43 and p36, lines 754-6).

Table S7. Leave-one-out-cross-validation of predictive models.

Model	n features	AUC LOOCV	95% Confidence Interval	
Transcriptome	128	0.685	0.556	0.815
Proteome	14	0.771	0.659	0.882
Phosphoproteome	2	0.648	0.507	0.788
Proteogeomics	128+14+2	0.844	0.741	0.946
Protein-centric	14+2	0.797	0.696	0.898

Acronyms: AUC: area under the curve; LOOCV: leave-one-out-cross-validation.

Reviewer #2 (Remarks to the Author):

This study extends prior preclinical findings by validating them in patient tumor samples rather than relying solely on cell-line models. It identifies increased overall mutational burden as a feature of ESR1-mutant disease and narrows the focus to MTORC1 activation, including a proposed estrogen-dependent component that may contribute to tumor aggressiveness. The analysis links MTORC1 signaling to higher tumor grade and recurrence risk and introduces a proteogenomic signature intended for patient selection and risk stratification, highlighting its potential clinical relevance.

There are few major concerns for this study:

1. Unclear Tissue Processing and Preservation - The manuscript does not specify whether samples were fresh frozen, FFPE, or otherwise preserved. Since phosphoproteomic signals are highly sensitive to handling and fixation, these pre-analytical details are critical for interpreting MTORC1 activation results.

We thank the reviewer for this comment and apologize for omitting this critical information on tissue processing. All samples were collected as fresh specimens at tumor surgical resection, processed via Allprep the same day and protein flowthroughs were stored at -80C until processing (mass spectrometry). We have now added this in our Methods section (p28, lines 567-9).

2. Lack of Omics Quality Control Reporting - Essential QC metrics for RNA-seq, proteomics, and phosphoproteomics are not presented. Information on sequencing quality, protein/phosphosite identification confidence, normalization, batch correction, and reproducibility is necessary to assess data robustness.

We agree with the reviewer that a more comprehensive QC analysis of the data layers should have been added to the manuscript.

RNAseq was performed following previously published protocols (PMID: 25722745; PMID: 32926574), but we have added a paragraph in the Methods section explaining tumor tissue processing from OR collection to RNAseq, computation of gene expression tables, and variant calling (p28-9, lines 580-95; p33-4, lines 696-702).

On the protein data layers (proteome and phosphoproteome), we have added QC plots to Figure S1 (below and in *Supplementary Material*), and expanded the Methods and Results sections discussing data processing, assessment/correction of batch effects, and overall protein quantitation performance using CV% and waterfall plots (p8-9, lines 116-27).

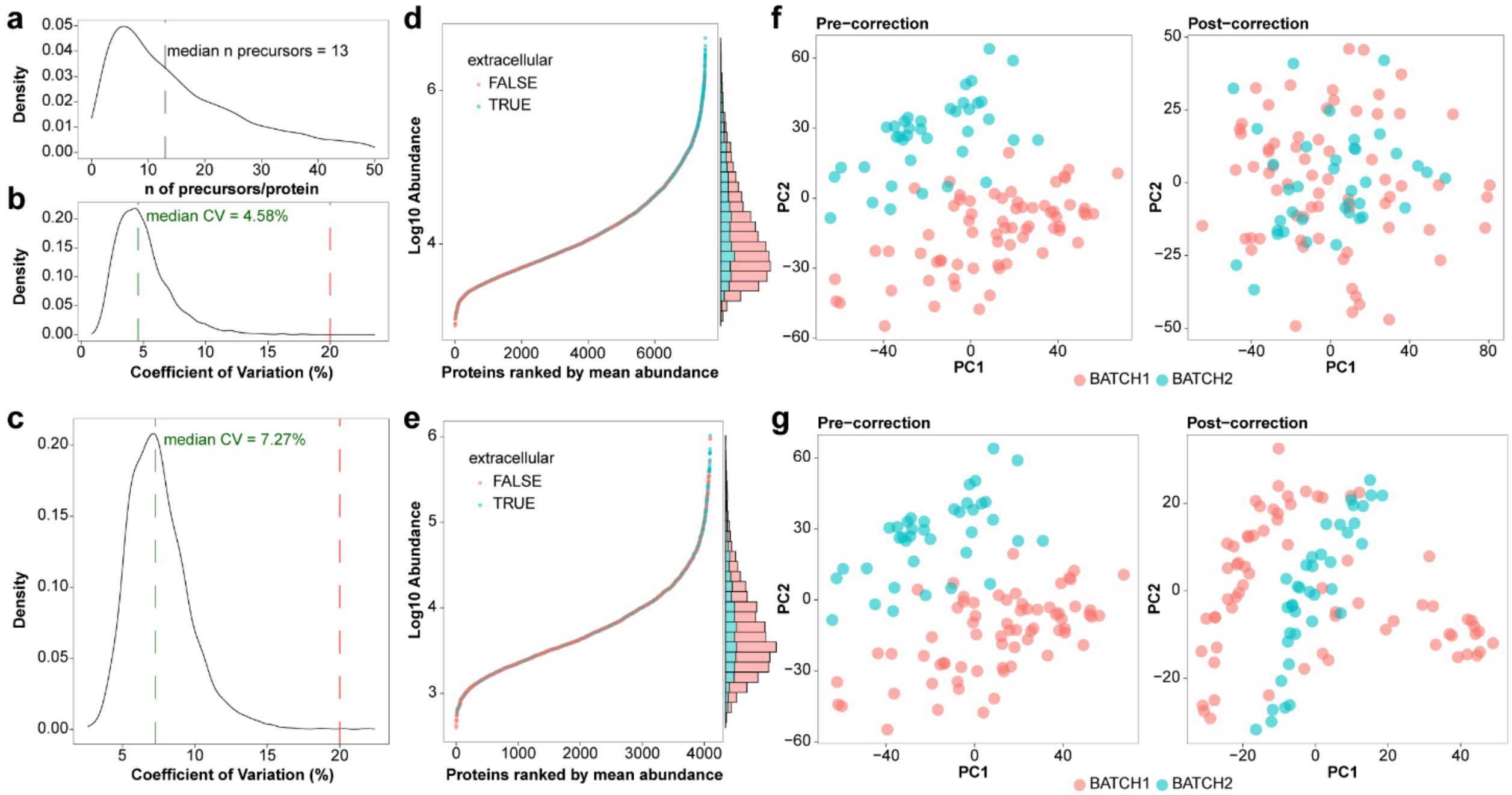

Figure S1. Quality control plots for the proteomic data layers.

Figure displays plots for proteome and phosphoproteome quality control. Panela A displays the number of precursors per each protein (min: 1; median: 13; max: 821) out of the proteome layer. Panels B and C display protein and phosphosite CV distribution for proteins and phosphosites, respectively. Panels D and E display waterfall plots for the proteome and phosphoproteome data layers, respectively. Panels G and F show PCA plots of proteome and phosphoproteome before (left panels) and after (right panels) batch correction, respectively.

Acronyms: CV: coefficient of variation.

3. Potential Circular Validation (Figure 5) - Figure 5 appears to validate MTORC1 elevation using a dataset that may overlap with the group's prior preclinical study. If not fully independent, this risks circular validation rather than true confirmation. The authors should clarify dataset independence and how this analysis represents external validation.

We thank the reviewer for this comment. The cell-line cohort was generated independently of the clinical SCAN-B cohort and re-analyzed in this study just to evaluate MTORC1 pathway activation, which was then found consistent with observations in the clinical dataset. We have specified this in the text (p17, lines 334-36).

Minor concern:

Cis and trans association across omes should be reported

We do agree with the reviewer that cis/trans regulation should be described in a proteogenomic dataset. We have computed RNA-protein and protein-phosphosite correlations to assess the degree of agreement between data layers. Distributions of correlation coefficients showed that most

regulation was in cis, as changes in (e.g.) transcriptome was followed by a similar change in protein abundance. Still, a small cluster of transcript-protein and protein-phosphosite pairs showed negative correlations. We performed metascape analysis on these subsets to define the most enriched pathways. We have added this to the Results section (p10, lines 152-64; Figure S2).

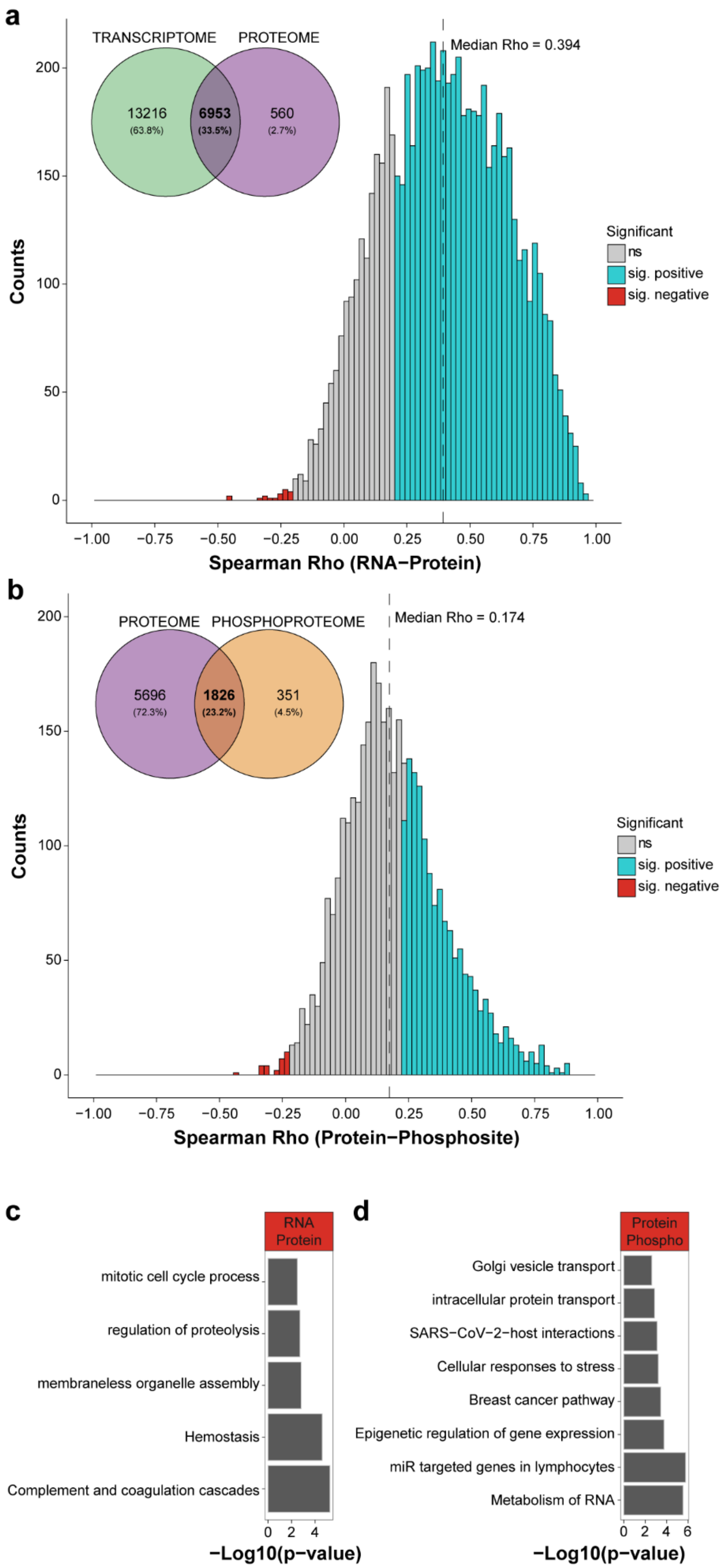
a
TRANSCRIPTOME
PROTEOME
13216
(63.8%)
6953
(33.5%)
560
(2.7%)
Median Rho = 0.394
Counts
Spearman Rho (RNA−Protein)
Significant
ns
sig. positive
sig. negative
b
PROTEOME
PHOSPHOPROTEOME
5696
(72.3%)
1826
(23.2%)
351
(4.5%)
Median Rho = 0.174
Counts
Spearman Rho (Protein−Phosphosite)
Significant
ns
sig. positive
sig. negative
c
RNA
Protein
mitotic cell cycle process
regulation of proteolysis
membraneless organelle assembly
Hemostasis
Complement and coagulation cascades
−Log10(p−value)
d
Protein
Phospho
Golgi vesicle transport
intracellular protein transport
SARS−CoV−2−host interactions
Cellular responses to stress
Breast cancer pathway
Epigenetic regulation of gene expression
miR targeted genes in lymphocytes
Metabolism of RNA
−Log10(p−value)

Figure S2. Correlation between data layers.

The unions of the transcriptome and proteome (Venn, panel A) and the proteome and phosphoproteome (Venn, panel B) data layers were employed to compute feature-wise correlations. Distributions of Spearman Rho coefficients were then plotted in histograms (transcriptome-proteome in panel A and proteome-phosphoproteome in panel B). Blue (agreement) and red (disagreement) bars indicate significant (adjusted p-value < 0.05) correlations. Significant negative correlating features were then analyzed by metascape and plotted in bar charts (transcriptome-proteome in panel C and proteome-phosphoproteome in panel D)

In addition to this, we performed a similar analysis for the MTORC1 pathway to assess any possible regulation via the estrogen receptor. Here we observed no direct relationship between MTORC1 signaling (i.e. GSVA scores computed based on RNA or protein data) and *ESR1* transcript levels, though we noticed a positive one with the estrogen response pathway, indicating that downstream effects of estrogen signaling participate in MTORC1 signaling activation. Interestingly, some transcripts and proteins correlated with MTORC1 associated kinases, indicating partial regulation. We have added this to the Results section (p17, 318-33; Figure 4G-H).

Reviewer #3 (Remarks to the Author):

This study presents a multi-omics analysis of ESR1-mutant breast cancers integrating transcriptomic, proteomic and phosphoproteomic data from the SCAN-B cohort. The study provides evidence that MTORC1 signaling is enriched in ESR1-mutant tumors and may contribute to tumor aggressiveness. While this link is not entirely novel, it is expanded and further characterized in the current study, which also provides relevant molecular signatures associated with clinical outcome.

1. The current study confirms and extends the ER–mTOR association using an integrated transcriptomic, proteomic and phosphoproteomic approach in a clinically relevant cohort. However, the extent to which the findings represent fundamentally new biological insights versus a multi-layered confirmation of previously reported mechanisms should be better clarified and, if possible, expanded. The study may benefit from assessing functional and statistical interactions within the integrated dataset.

We share the reviewer's statement and agree that several parts of the manuscript should be clarified. We have clarified each Results section with regards to the relationship between *ESR1* mutations and tumor grade (p11, lines 185-7; p12, lines 203-13) and the MTORC1 signaling pathway (p16, line 305-9; p17, lines 318-33; p18, lines 360-4). The Discussion section was also modified: p23, lines 455-60; p24, lines 507-9, p25-6, lines 512-28.

In addition to this, we have analyzed the relationship between data layers to assess the levels of regulation dependent on gene expression or other factors (p10, lines 152-64; Figure S2).

2. The study also proposes predictive signatures of clinical outcome. However, it appears that feature selection and model development were largely performed within the same dataset used for evaluation. This raises concerns about potential overfitting and inflated predictive performance. Although validation in external transcriptomic datasets is presented, the full multi-omic predictor cannot be validated due to the lack of comparable datasets. The authors may clarify the training and validation strategy used and provide more details regarding cross-validation procedures and model stability.

We have added information regarding predictive models (transcriptome, proteome, phosphoproteome, proteogenomic and proteo-centric) generation and leave-one-out-cross-validation (Results: p20, lines 396-8; Table S7; Methods: p35, lines 739-43 and lines 754-6).

Although transcriptomic features constituted the majority of predictive variables, the inclusion of proteomic and phosphoproteomic layers provided complementary information as shown in the proteogenomic predictor (Figure 6) and in the associations between the ER pathway and MTORC1 signaling (Figures 4-5). The lack of external verification of the proteo-centric and proteogenomic predictors is due to the lack of large enough multi-omic datasets, which we clarified on p18 (lines 375-7). While we recognize the limited applicability of this approach and the necessity for additional validation, we believe our workflow to be apt for the discovery of multi-omic biomarkers (p4, lines 44-5; p21, lines 421-2; p27, lines 557-9 and 557-9).

3. The observation of an association with tumor mutational burden (TMB) is interesting but should be interpreted cautiously. It is not entirely clear whether the increased mutation burden observed here reflects a direct biological consequence of ESR1 mutations, underlying tumor heterogeneity, or other confounding factors. In addition, mutational burden is inferred from RNA-seq data, which may introduce biases. Comparison with other cohorts and data types would strengthen this observation.

We agree with the reviewer that RNAseq derived mutational calling should be analyzed with care as it is more prone to identifying false positives and thus less accurate when compared to whole genome and exome sequencing. Still, we have also added a more detailed description of how the RNAseq data was processed and how filtering for mutational calling was performed to minimize false positives. We have specified this in our Results (p13, lines 226-30), Methods (p28-9, lines 580-95; p33-4, lines 696-702), and Discussion (p23-4, lines 469-71) sections. We do agree on the fact that these observations are preliminary in nature and need to be validated, but comparison with other cohorts remains difficult as ESR1 mutations are not always reported or in too few numbers (e.g. METABRIC, TCGA) or exist in metastatic cohorts (e.g. MET500, POG570), where larger genome-wide aberrations have taken place. We have evaluated the TMB within these two datasets that included *ESR1* mutations in metastatic breast cancers, but we found no difference between tumors with *wt* and mutated ER (POG570 Wilcoxon p-value = 0.253; MET500 Wilcoxon p-value = 0.192). Due to this and the fact that multiple processes related to invasion, immune evasion, and therapy resistance are operating in metastatic breast cancer, we have decided to not include these data in our manuscript.

4. Although the study generates a rich multi-omics dataset, the predictive models appear to rely predominantly on transcriptomic features. This raises the question of whether the predictive performance is largely driven by the transcriptomic component rather than by true integration across data layers. The authors should clarify how features from different omics layers were selected and integrated into the predictive models, and whether models based on each individual layer were systematically compared to assess the added value of the proteomic and phosphoproteomic data generated in this study. In addition, describing cross-omic interactions could add a novel layer of interpretation linking functional connections and clinical outcomes.

We thank the reviewer for this comment, but the reliance on transcriptomics relies on the fact that large proteogenomic or well annotated proteomic datasets are not available to date, hence the limitation of this study (now more clearly stated on p27, lines 547-52).

With regards to predictive performance, we would like to point out that each model was independently tested (transcriptome, proteome, and phosphoproteome; p18, lines 365-75; Figure S13 and Table S7), then merged into a model that included all three data layers (p19, lines 382-88; Figure 6, Figure S14, and Tables S7-9) or just the proteome and phosphoproteome ones (p19, lines 389-98; Figure S15 and Tables S7, S11, and S12). All model tests, including LOOCV, were performed independently, indicating the validity of our approach in defining biomarker candidates, but still necessitating further validation.

As for the feature selection and validation of each predictive model, we have extended our Methods section (p34, lines 703-9; p35, lines 739-43).

In addition to this, we have added correlation analyses across the omics layers as a representation of cis and trans interactions for the complete datasets (p10, lines 152-64; Figure S2) and in relation to estrogen signaling and the MTORC1 pathway (p17, lines 318-333; Figure 4G-H; p18, lines 359-64; p18, lines 369-70; p20, lines 395-8; p27, lines 547-52). This in turn reinforces the complementarity of multiple omics layers, not only in the generation of predictive models, but also in defining pathway regulation, such as the case of MTORC1.

5. The study applies Multi-Omics Factor Analysis (MOFA) to identify major sources of variation across the transcriptomic, proteomic and phosphoproteomic layers. While this is an appropriate framework, it should be noted that the number of ESR1-mutant tumors included in the study is relatively limited. Given the high dimensionality of the dataset and the relatively small number of mutant samples, it would be helpful for the authors to further discuss the robustness of the factor decomposition and the stability of the identified factors. Additional information on how sensitive the MOFA results are to sample composition, or whether robustness analyses were performed, would strengthen confidence into the analyses.

We thank the reviewer for this comment and we do agree with the fact that the number of *ESR1* mutant cases is relatively small to provide broad generalizations. Despite of this, while *ESR1* mutations are rare in primary tumors, the ones in metastatic breast cancer have not been characterized in-depth. This study is the first one to provide a clinical sample-oriented analysis complementary to the ones performed in preclinical models.

With regards to MOFA robustness, we apologize for not clarifying this. We have run 100 iterations of the MOFA models with different random seeds, which yielded identical variances explained across data layers, in turn indicating a stable factor decomposition. Additionally, we ran 5 iterations of the MOFA model with random resampling, which showed excellent correlation with the full model for the dominant factor weights (factor 1). We have added this to the Results (p10, lines 165-71) and Methods sections (p34, lines 710-5).

Reviewers' comments:

Reviewer #1 (Remarks to the Author)

Thank you for the detailed revision. The additional analyses and revised discussion have improved the manuscript and the authors now address several of my previous concerns.

We thank the reviewer for their comments in helping us improve the manuscript in all its parts.

I still think the revised title expresses language stronger than the data suggests and implies a functional and causal relationship.

We agree with the reviewer on this point and have changed the title accordingly to denote an association rather than a causation. The revised title is: "*ESR1* mutations are associated with enhanced MTORC1 signaling in a context-dependent manner"

Secondly, I think the reporting of phosphoproteomic handling needs to be improved (time from resection to lysis/freezing, use of phosphotase and protease inhibitors, statement on any difference in processing time between samples).

We thank the reviewer for this important point. We have added this information in the Methods section (p 29, lines 585-91).

The Allprep standard protocol employs RTL buffer, which contains guanidinium thiocyanate, and beta-mercaptoethanol is added by the user. This ensures rapid protein denaturation, including proteases and phosphatases. From this we argue that remaining protease/phosphatase activity might be negligible.

Nonetheless we have investigated this further and analyzed the impact of ischemic time on the identification and quantification of both protein and phosphosites.

Spearman correlation coefficients between time from excision to RNAlater (i.e. ischemic time) and the number of identified proteins (i.e. having non-missing quantification values) showed that no association was found with protein (Spearman Rho = -0.022, p-value = 0.831) and phosphosite (Spearman Rho = -0.204, p-value = 0.047) identifications.

Furthermore, we assessed whether clustering or sub-clustering of samples was associated to ischemic time in our PCA analyses, but no clear groupings was found for either the proteome or the phosphoproteome dataset (Fig rev1). We have added Spearman correlation results between number of identified proteins/phosphosites and ischemic time in our Results section (p 8, lines 118-9).

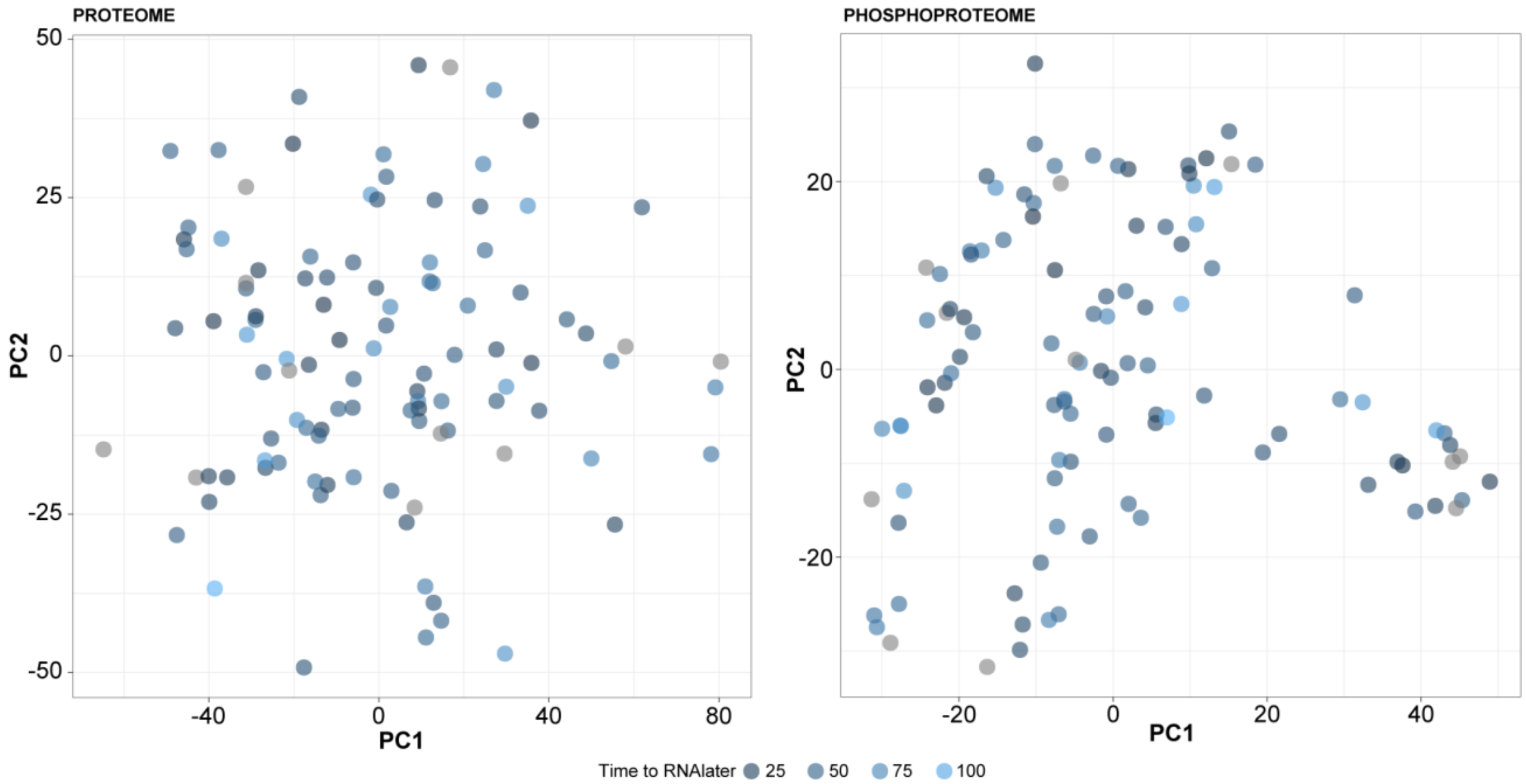

Fig rev1. Principal component analysis of the proteome and phosphoproteome datasets in relation to ischemic time, defined here as time to RNAlater.

Reviewer #2 (Remarks to the Author)

The authors have addressed the all my major concerns.

We thank the reviewer for this and for taking the time to review the manuscript first submission and revised version.

Reviewer #3 (Remarks to the Author)

The authors have performed substantial additional analyses and improved the framing of the study, enhancing its overall presentation and interpretability.

We thank the reviewer for giving us the opportunity to improve our manuscript and to further ameliorate it with this revision.

Nevertheless, in my opinion, the claim that the integrated proteogenomic model provides superior predictive performance remains insufficiently supported. Although the integrated and protein-centric models show higher LOOCV AUC values than the transcriptomic model, the confidence intervals overlap, no formal comparison of predictive performance is presented, and external validation remains restricted to the transcriptomic signature.

We thank the reviewer for this important comment. We agree that differences in LOOCV AUC values alone do not establish superior predictive performance and that formal comparison of the models is required. We have therefore performed paired comparisons of the LOOCV predictions using DeLong's test, which showed that the integrated predictor has significantly better prediction than the RNA and phosphoproteome single models, but not that of the proteome one. We have specified this in the Results section (p 20, lines 406-11).

In addition to this, we have modified our claims to better reflect these results (p 21, lines 414-6).

We have also moderated our claims in the Discussion section, highlighting the need for external multi-omic validation (p27, line 553, lines 555-61, lines 563-5 and lines 568-71).

The Methods section has also been expanded to include the description of these new analyses (p 37, lines 776-9).

As acknowledged by the authors, I recognize that such validation is challenging given the nature and breadth of the required datasets. It also remains unclear whether feature selection, scaling, and cutoff determination were repeated within each cross-validation fold. The results therefore support a numerical improvement in internal performance, but generalizability cannot yet be claimed, particularly as the analyses are limited to a single dataset. Thus, while the results and conclusions are potentially valuable, further studies will be required to extend and independently confirm them, and the corresponding language should be moderated accordingly.

We agree that independent validation of the integrated model remains an important limitation. The available external datasets do not contain the matched transcriptomic, proteomic and phosphoproteomic measurements required to independently evaluate the integrated predictor. We have therefore moderated the corresponding claims of generalizability and now explicitly state that the integrated model requires independent validation in cohorts with matched multi-omic and clinical data (p27, line 553, lines 555-61, lines 563-5, and lines 568-71).

With regard to model performance analysis, the predictors evaluated in the LOOCV analysis were predefined based on the discovery analyses described in the previous paragraph (i.e. differential expression testing and Cox regressions). Within each LOOCV iteration, the logistic regression model was fitted exclusively on the training samples, and the resulting model was used to generate a prediction for the held-out sample. We have clarified this distinction in the Methods section and now explicitly state that the LOOCV was used to evaluate the predictive performance of the predefined signatures, rather than to repeat the upstream discovery/feature-selection procedure within each fold (p 37, lines 779-80).

We have also edited the text to moderate our claims in the Introduction section (p7, lines 99-102).
