# Supplementary material for: ESR1 mutations are associated with enhanced MTORC1 signaling in a context-dependent manner
Source: Commun Biol. 2026 Oct 7;9:1304. doi: 10.1038/s42003-026-11096-w (PMC13645737; doi:10.1038/s42003-026-11096-w)
Supplement: Supplementary file 2 [file 42003_2026_11096_MOESM2_ESM.pdf]

TITLE: "*ESR1* mutations are associated with enhanced MTORC1 signaling in a context-dependent manner"

Authors

Tommaso De Marchi[1,9,10], Magdalena Kuras[2], Lazaro H. Betancourt[3], Sven Kjellström[4], Jeanette Valcich[5,9,10], Johan Vallon-Christersson[5,9,10], Christian Brueffer[5,9,10], Lao H. Saal[5,6,9,10], Johan Malmström[7], Emma Niméus[1,8,9,10]

Author affiliations

1. Division of Surgery, Oncology, and Pathology, Department of Clinical Sciences, Lund University, Solvegatan 19, SE 22362, Lund, Sweden.

2. Department of Biomedical Engineering, Lund University, SE 22100, Lund, Sweden

3. Department of Translational Medicine, Lund University, Skåne University Hospital Malmö, Malmö, Sweden.

4. Swedish National Infrastructure for Biological Mass Spectrometry – BioMS, Lund, Sweden.

5. Division of Oncology, Department of Clinical Sciences Lund, Lund University, Medicon Village, SE 22381, Lund, Sweden.

6. Center for Interdisciplinary Research on Cancer and Equity in Women, Lund University, Lund, Sweden.

7. Division of Infection Medicine, Department of Clinical Sciences Lund, Faculty of Medicine, Lund University, Klinikgatan 32, SE 22184, Lund, Sweden.

8. Department of Surgery, Skåne University Hospital, Lund, Sweden.

9. Lund University Cancer Center, Lund, Sweden

10. Skåne University Hospital Comprehensive Cancer Center, Lund, Sweden

Supplementary Tables

Supplementary Table 1. Univariable Cox regression of clinical and histopathological characteristics for relapse-free survival.

		95% Confidence Interval		
***ESR1* status**	**Hazard Ratio***	**lower**	**upper**	***P***
wt	1			
**mutant**	2.743	1.161	6.482	0.021
**Age at diagnosis†**				
**<55**	1			
**≥55**	5.055	1.184	21.57	0.029
**Menopausal status†**				
**Premenopausal**	1			
**Postmenopausal**	3.233	0.758	13.79	0.113
**PgR status**				
**Negative**	1			
**Positive**	>>10	0	Inf	0.998
**ERBB2 status**				
**Normal**	1			
**Amplified**	1.858	0.813	4.246	0.142
**KI67 status†**				
**Low**	1			
**High**	1.336	0.156	11.45	0.791
**Tumor Grade**				
**Low**	1			
**Moderate**	>>10	0	Inf	1
**High**	>>10	0	Inf	1
**Tumor Size**				
**T0+Tis**	1			
**T1**	1.172	0.243	5.647	0.843
**T2+Tx**	3.557	0.800	15.815	0.095
**T3+T4**	1.681	0.152	18.550	0.672
**Positive lymph-nodes**				
**N0**	1			
**N1**	2.273	0.935	5.530	0.07

**Adjuvant Endocrine†**				
**No**	1			
**Yes**	>>10	0	Inf	1.000
**Adjuvant Radiotherapy†**				
**No**	1			
**Yes**	0.282	0.122	0.652	0.003

*Univariable Cox proportional hazards regression, two-sided. Reported *P*-values are non-adjusted.

†Missing data

Acronyms: ER: Estrogen Receptor; KI67: tumor marker Ki67; PgR: Progesterone Receptor.

Supplementary Table 2. Multivariable Cox regression of clinical and histopathological characteristics for relapse-free survival.

		95% Confidence Interval		
***ESR1* status**	**Hazard Ratio***	**lower**	**upper**	***P***
wt	1			
**mutant**	2.626	1.097	6.283	0.030
**Age at diagnosis**				
**<55**	1			
**≥55**	3.421	0.774	15.116	0.105
**Adjuvant Radiotherapy†**				
**No**	1			
**Yes**	0.328	0.139	0.772	0.011

*Multivariable Cox proportional hazards regression, two-sided. Reported *P*-values are non-adjusted.

†Missing data

Supplementary Table 3. Univariable Cox regression of clinical and histopathological characteristics for overall survival.

		95% Confidence Interval		
***ESR1* status**	**Hazard Ratio***	**lower**	**upper**	***P***
wt	1			
**mutant**	2.98	1.167	7.611	0.022
**Age at diagnosis†**				
**<55**	1			
**≥55**	3.985	0.92	17.26	0.064
**Menopausal status†**				
**Premenopausal**	1			
**Postmenopausal**	2.567	0.593	11.11	0.208
**PgR status**				
**Negative**	1			
**Positive**	3.90E-08	0	Inf	0.998
**ERBB2 status**				
**Normal**	1			
**Amplified**	1.70E+00	0.679	4.253	0.257
**KI67 status†**				
**Low**	1			
**High**	1.046	0.117	9.368	0.968
**Tumor Grade**				
**Low**	1			
**Moderate**	>>10	0	Inf	0.998
**High**	>>10	0	Inf	0.998
**Tumor Size**				
**T0+Tis**	1			
**T1**	1.943	0.234	16.15	0.539
**T2+Tx**	5.569	0.717	43.22	0.101
**T3+T4**	3.28	0.205	52.52	0.401
**Positive lymph-nodes**				
**N0**	1			
**N1**	2.338	0.887	6.156	0.086

**Adjuvant Endocrine†**				
**No**	1			
**Yes**	>>10	0	Inf	0.998
**Adjuvant Radiotherapy†**				
**No**	1			
**Yes**	0.201	0.076	0.529	0.001

*Univariable Cox proportional hazards regression, two-sided. Reported *P*-values are non-adjusted.

†Missing data

Acronyms: ER: Estrogen Receptor; KI67: tumor marker Ki67; PgR: Progesterone Receptor.

Supplementary Table 4. Multivariable Cox regression of clinical and histopathological characteristics for overall survival.

		95% Confidence Interval		
***ESR1* status**	**Hazard Ratio***	**lower**	**upper**	***P***
wt	1			
**mutant**	3.302	1.276	8.546	0.014
**Adjuvant Radiotherapy†**				
**No**	1			
**Yes**	0.189	0.071	0.499	0.001

*Multivariable Cox proportional hazards regression, two-sided. Reported *P*-values are non-adjusted.

†Missing data

Supplementary Table 5. Leave-one-out-cross-validation of predictive models.

Model	n features	AUC LOOCV	95% Confidence Interval	
Transcriptome	128	0.685	0.556	0.815
Proteome	14	0.752	0.659	0.882
Phosphoproteome	2	0.648	0.507	0.788
Proteogenomics	128+14+2	0.844	0.741	0.946
Protein-centric	14+2	0.797	0.696	0.898

Acronyms: AUC: area under the curve; LOOCV: leave-one-out-cross-validation.

Supplementary Table 6. Multivariable Cox regression of the proteogenomic predictor characteristics for relapse-free survival.

		95% Confidence Interval		
***ESR1* status**	**Hazard Ratio***	**lower**	**upper**	***P***
wt	1			
**mutant**	0.905	0.337	2.436	0.844
**Adjuvant Radiotherapy†**				
**No**	1			
**Yes**	0.378	0.158	0.908	0.029
**Proteogenomic predictor**				
**Low risk**	1			
**High risk**	9.19	3.43	24.642	<0.001

*Multivariable Cox proportional hazards regression, two-sided. Reported *P*-values are non-adjusted.

†Missing data

Supplementary Table 7. Multivariable Cox regression of the proteogenomic predictor for overall survival.

		95% Confidence Interval		
***ESR1* status**	**Hazard Ratio***	**lower**	**upper**	***P***
wt	1			
**mutant**	1.016	0.349	2.953	0.977
**Adjuvant Radiotherapy†**				
**No**	1			
**Yes**	0.253	0.093	0.684	0.007
**Proteogenomic predictor**				
**Low risk**	1			
**High risk**	8.871	2.973	26.467	<0.001

*Multivariable Cox proportional hazards regression, two-sided. Reported *P*-values are non-adjusted.

†Missing data

Supplementary Table 8. Multivariable Cox regression of the proteo-centric predictor for relapse-free survival.

***ESR1* status**	**Hazard Ratio***	**95% Confidence Interval** lower	upper	***P***
wt	1			
**mutant**	1.424	0.577	3.513	0.442
**Adjuvant Radiotherapy†**				
**No**	1			
**Yes**	3.01	0.143	0.773	0.01
**Proteo-centric predictor**				
**Low risk**	1			
**High risk**	6.529	2.104	22.25	0.001

*Multivariable Cox proportional hazards regression, two-sided. Reported *P*-values are non-adjusted.

†Missing data

Supplementary Table 9. Multivariable Cox regression of the proteo-centric predictor for overall survival.

		95% Confidence Interval		
***ESR1* status**	**Hazard Ratio***	**lower**	**upper**	***P***
wt	1			
**mutant**	1.547	0.575	4.167	0.387
**Adjuvant Radiotherapy†**				
**No**	1			
**Yes**	0.232	0.087	0.613	0.003
**Proteoe-centric predictor**				
**Low risk**	1			
**High risk**	6.84	1.872	24.987	<0.001

*Multivariable Cox proportional hazards regression, two-sided. Reported *P*-values are non-adjusted.

†Missing data

Supplementary Figures

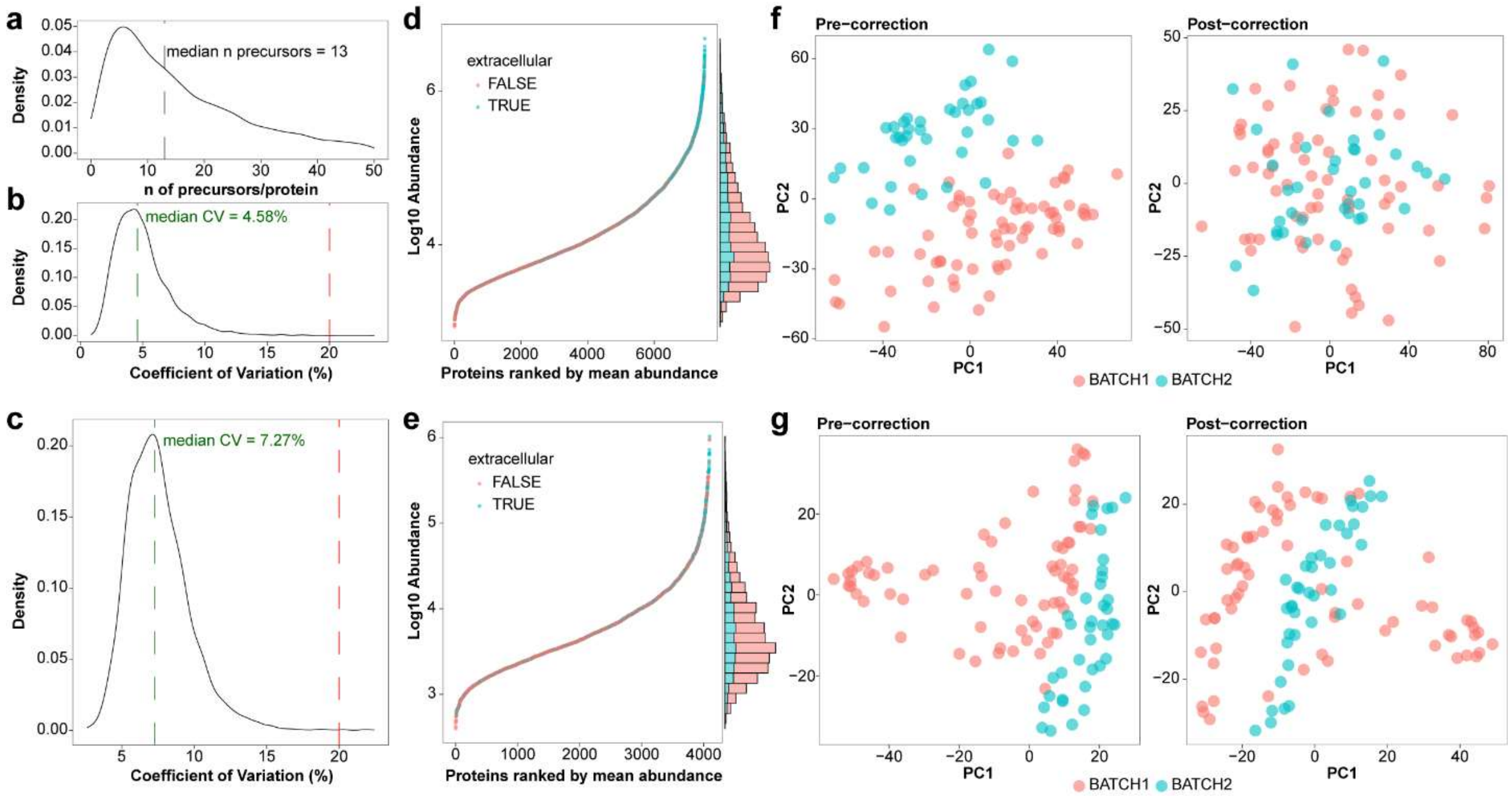

Supplementary Figure 1. Quality control plots for the proteomic data layers.

Figure displays plots for proteome and phosphoproteome quality control. Panela A displays the number of precursors per each protein (min: 1; median: 13; max: 821) out of the proteome layer. Panels B and C display protein and phosphosite CV distribution for proteins and phosphosites, respectively. Panels D and E display waterfall plots for the proteome and phosphoproteome data layers, respectively. Panels G and F show PCA plots of proteome and phosphoproteome before (left panels) and after (right panels) batch correction, respectively.

Acronyms: CV: coefficient of variation.

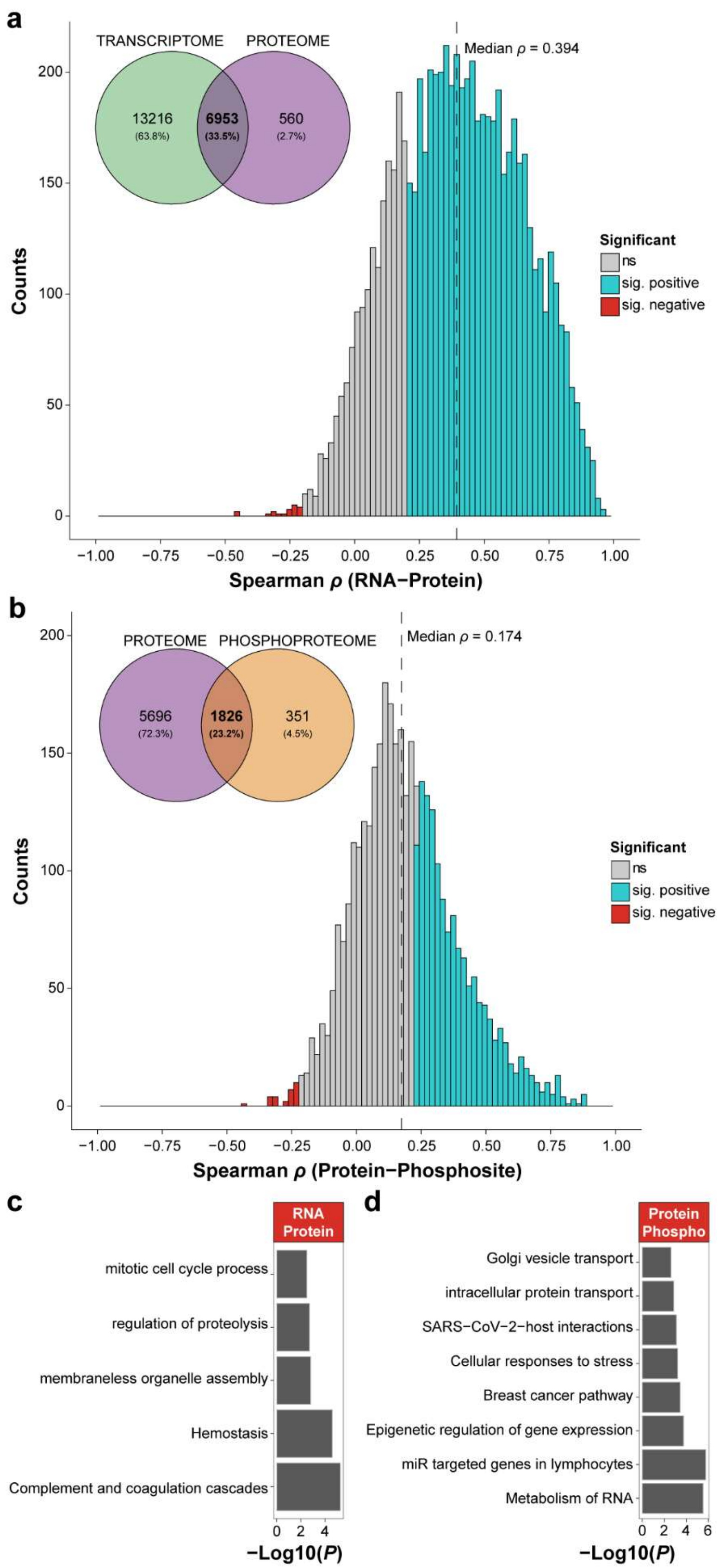
a
TRANSCRIPTOME
PROTEOME
13216
(63.8%)
6953
(33.5%)
560
(2.7%)
Median ρ = 0.394
Counts
Significant
ns
sig. positive
sig. negative
Spearman ρ (RNA−Protein)
b
PROTEOME
PHOSPHOPROTEOME
5696
(72.3%)
1826
(23.2%)
351
(4.5%)
Median ρ = 0.174
Counts
Significant
ns
sig. positive
sig. negative
Spearman ρ (Protein−Phosphosite)
c
RNA
Protein
mitotic cell cycle process
regulation of proteolysis
membraneless organelle assembly
Hemostasis
Complement and coagulation cascades
−Log10(P)
d
Protein
Phospho
Golgi vesicle transport
intracellular protein transport
SARS−CoV−2−host interactions
Cellular responses to stress
Breast cancer pathway
Epigenetic regulation of gene expression
miR targeted genes in lymphocytes
Metabolism of RNA
−Log10(P)

Supplementary Figure 2. Correlation between data layers.

The unions of the transcriptome and proteome (Venn, panel A) and the proteome and phosphoproteome (Venn, panel B) data layers were employed to compute feature-wise correlations. Distributions of Spearman ρ coefficients were then plotted in histograms (transcriptome-proteome in panel A and proteome-phosphoproteome in panel B). Blue (agreement) and red (disagreement) bars indicate significant (adjusted p-value < 0.05) correlations. Significant negative correlating features were then analyzed by metascape and plotted in bar charts (transcriptome-proteome in panel C and proteome-phosphoproteome in panel D)

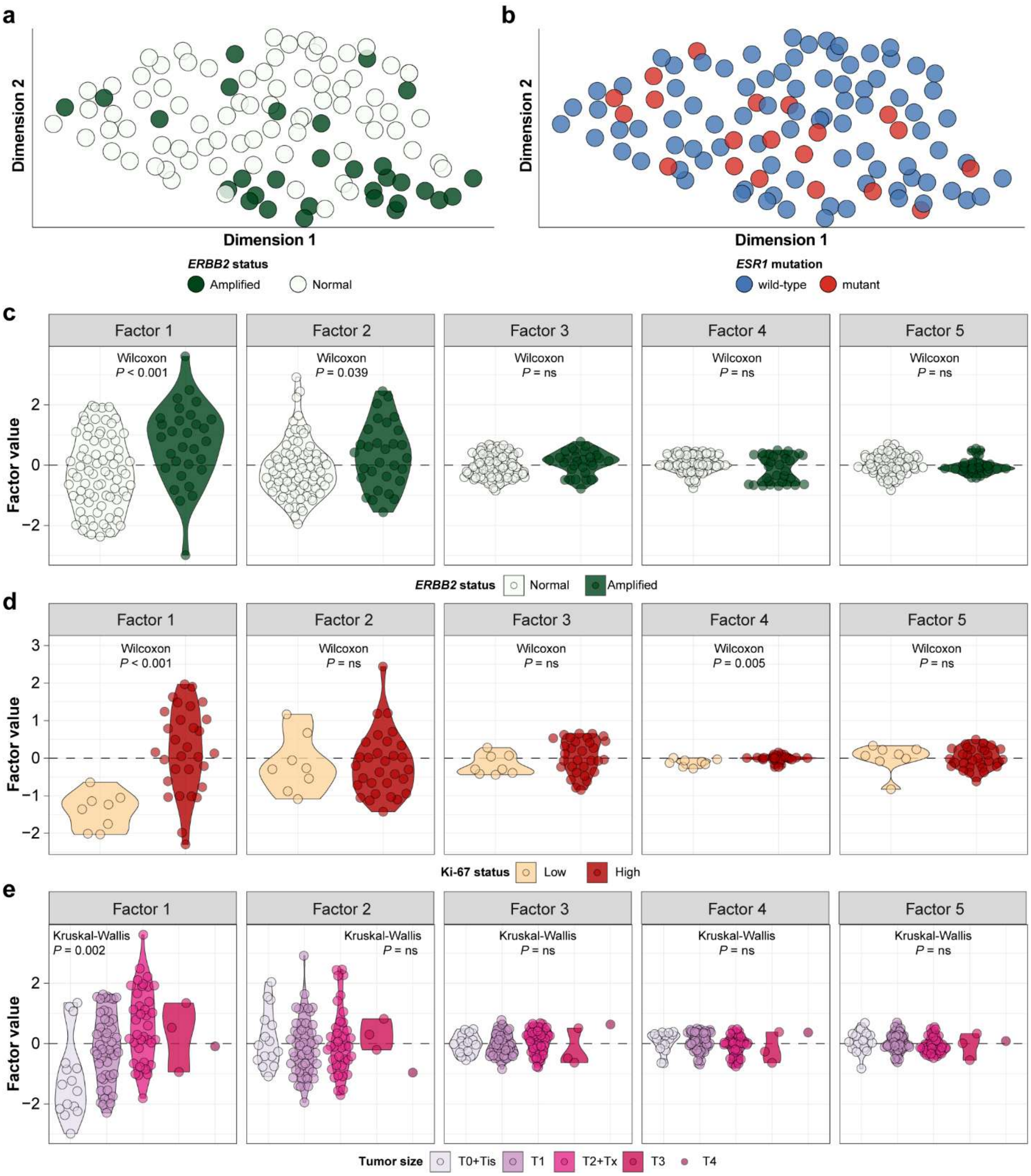
a
Dimension 2
Dimension 1
ERBB2 status
Amplified
Normal
b
Dimension 2
Dimension 1
ESR1 mutation
wild-type
mutant
c
Factor 1
Wilcoxon
P < 0.001
Factor 2
Wilcoxon
P = 0.039
Factor 3
Wilcoxon
P = ns
Factor 4
Wilcoxon
P = ns
Factor 5
Wilcoxon
P = ns
Factor value
ERBB2 status
Normal
Amplified
d
Factor 1
Wilcoxon
P < 0.001
Factor 2
Wilcoxon
P = ns
Factor 3
Wilcoxon
P = ns
Factor 4
Wilcoxon
P = 0.005
Factor 5
Wilcoxon
P = ns
Factor value
Ki-67 status
Low
High
e
Factor 1
Kruskal-Wallis
P = 0.002
Factor 2
Kruskal-Wallis
P = ns
Factor 3
Kruskal-Wallis
P = ns
Factor 4
Kruskal-Wallis
P = ns
Factor 5
Kruskal-Wallis
P = ns
Factor value
Tumor size
T0+Tis
T1
T2+Tx
T3
T4

Supplementary Figure 3. Single factor analysis and association to clinical and histo-pathological variables. We performed MOFA analysis on all our data layers (SNV frequency, transcriptome, proteome, and phosphoproteome) and visualized the dataset axes of variation using UMAPs (*ERBB2* amplification and *ESR1* mutation statuses are shown in panels A and B, respectively). We then plotted loading weights for the top 5 factors against clinical variables and tested for significant associations. Panels C, D, and E display violin plots of factors 1-5 loading weights versus *ERBB2* gene amplification, KI67 status, and tumor size, respectively (two-sided Wilcoxon and Kruskal-Wallis tests were employed here to detect significant differences; n = 106).

Acronyms: MOFA: Multi-Omic Factor Analysis; UMAP: Uniform Manifold Approximation and Projection.

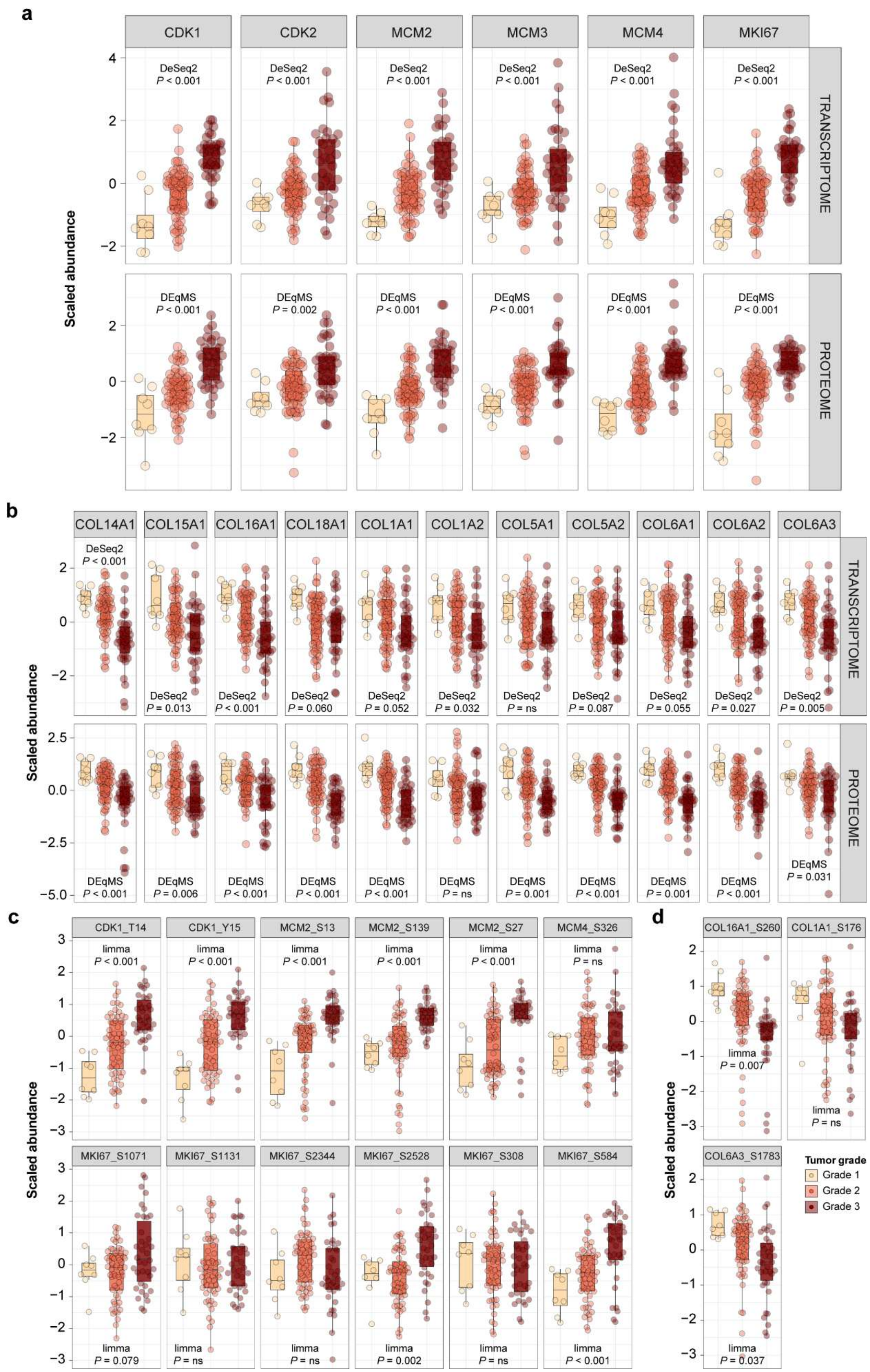
a
CDK1
CDK2
MCM2
MCM3
MCM4
MKI67
TRANSCRIPTOME
PROTEOME
Scaled abundance
DeSeq2 P < 0.001
DEqMS P < 0.001
DEqMS P = 0.002
b
COL14A1
COL15A1
COL16A1
COL18A1
COL1A1
COL1A2
COL5A1
COL5A2
COL6A1
COL6A2
COL6A3
DeSeq2 P = 0.013
DeSeq2 P = 0.060
DeSeq2 P = 0.052
DeSeq2 P = 0.032
DeSeq2 P = ns
DeSeq2 P = 0.087
DeSeq2 P = 0.055
DeSeq2 P = 0.027
DeSeq2 P = 0.005
DEqMS P = 0.006
DEqMS P = ns
DEqMS P = 0.001
DEqMS P = 0.031
c
CDK1_T14
CDK1_Y15
MCM2_S13
MCM2_S139
MCM2_S27
MCM4_S326
MKI67_S1071
MKI67_S1131
MKI67_S2344
MKI67_S2528
MKI67_S308
MKI67_S584
limma P < 0.001
limma P = ns
limma P = 0.079
limma P = 0.002
d
COL16A1_S260
COL1A1_S176
COL6A3_S1783
limma P = 0.007
limma P = 0.037
Tumor grade
Grade 1
Grade 2
Grade 3

Supplementary Figure 4. Proliferation and extracellular matrix variation based on tumor grade.

Transcript, protein, and phosphosite abundances of genes involved in proliferation and components of the extracellular matrix were extracted and plotted against tumor grade categories. Panel A depicts key transcripts and proteins involved in cell cycle progression, while panel B displays collagen expression across tumor grade categories. Phosphosite data for matching proteins and its associations to tumor grade is shown in panels C (cell proliferation) and D (collagens; p-values were calculated using ANOVA and adjusted using the Benjamini-Hochberg method).

Acronyms: n/a

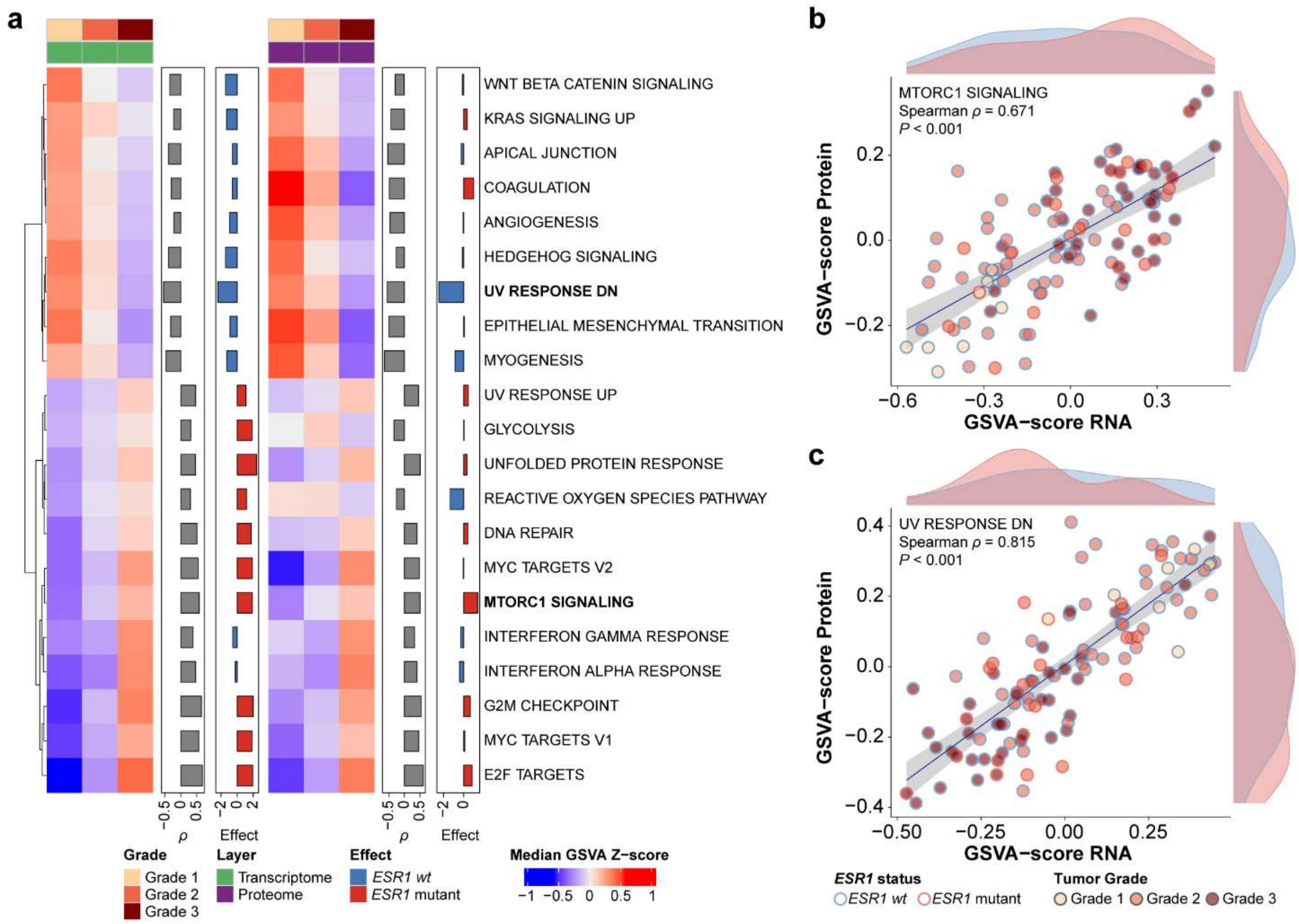
a
WNT BETA CATENIN SIGNALING
KRAS SIGNALING UP
APICAL JUNCTION
COAGULATION
ANGIOGENESIS
HEDGEHOG SIGNALING
UV RESPONSE DN
EPITHELIAL MESENCHYMAL TRANSITION
MYOGENESIS
UV RESPONSE UP
GLYCOLYSIS
UNFOLDED PROTEIN RESPONSE
REACTIVE OXYGEN SPECIES PATHWAY
DNA REPAIR
MYC TARGETS V2
MTORC1 SIGNALING
INTERFERON GAMMA RESPONSE
INTERFERON ALPHA RESPONSE
G2M CHECKPOINT
MYC TARGETS V1
E2F TARGETS
ρ
Effect
Grade
Grade 1
Grade 2
Grade 3
Layer
Transcriptome
Proteome
Effect
ESR1 wt
ESR1 mutant
Median GSVA Z-score
-1 -0.5 0 0.5 1
b
MTORC1 SIGNALING
Spearman ρ = 0.671
P < 0.001
GSVA-score Protein
GSVA-score RNA
c
UV RESPONSE DN
Spearman ρ = 0.815
P < 0.001
GSVA-score Protein
GSVA-score RNA
ESR1 status
ESR1 wt
ESR1 mutant
Tumor Grade
Grade 1
Grade 2
Grade 3

Supplementary Figure 5. Pathways regulated by tumor grade and impact of *ESR1* mutations.

Hallmarks RNA and protein GSVA scores were summarized (metric: median) and evaluated for linearity association. Pathways overlapping between RNA and protein data layers were plotted in a heatmap (panel A). Bar charts to the right of each heatmap depict Spearman ρ between tumor grade and GSVA scores (left) and linear model statistic to measure *ESR1* mutation effect on each pathway (right). Panels B and C show scatterplots of RNA and protein GSVA scores for pathways where *ESR1* mutations had an effect (linear model $|statistic| > 1$) both at the RNA and protein level.

Acronyms: GSVA: Gene Set Variation Analysis.

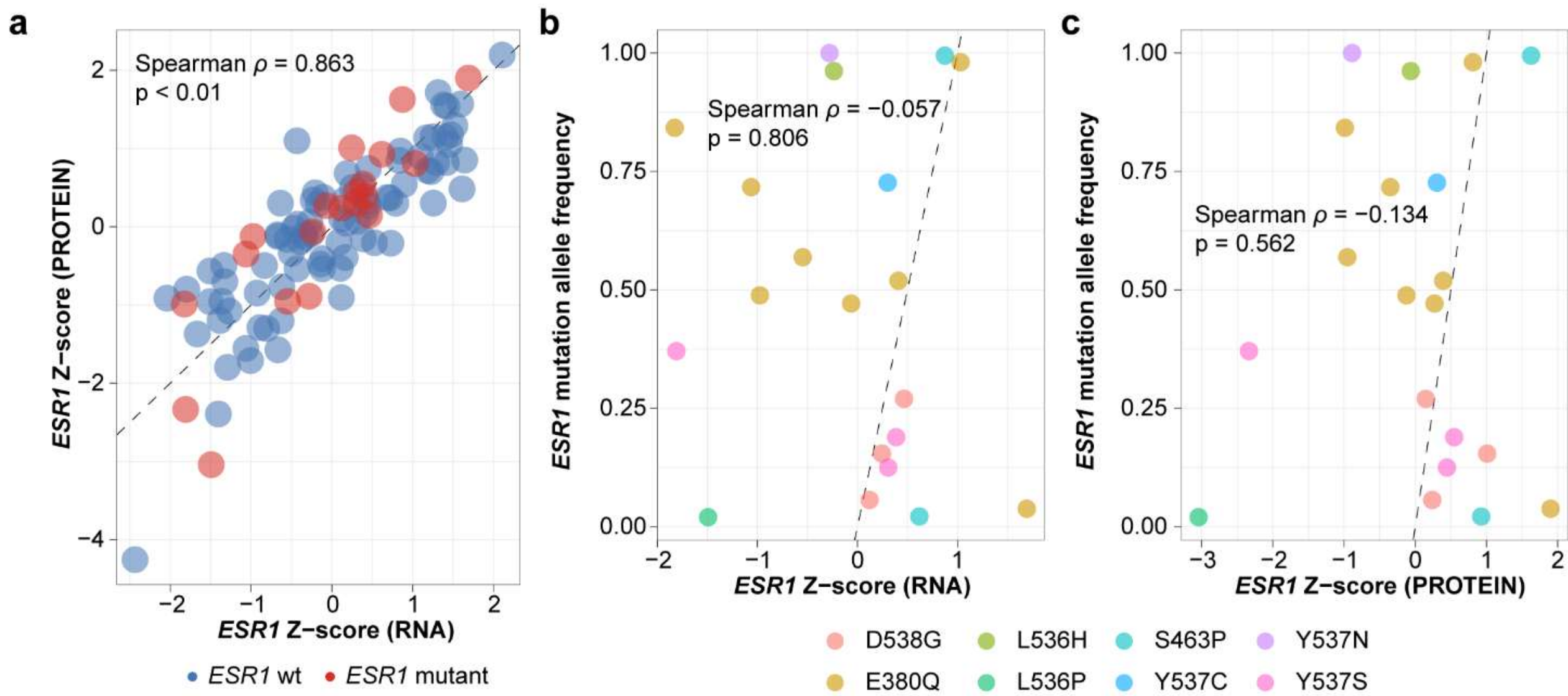
a
Spearman ρ = 0.863
p < 0.01
ESR1 Z-score (PROTEIN)
ESR1 Z-score (RNA)
ESR1 wt
ESR1 mutant
b
Spearman ρ = −0.057
p = 0.806
ESR1 mutation allele frequency
ESR1 Z-score (RNA)
c
Spearman ρ = −0.134
p = 0.562
ESR1 mutation allele frequency
ESR1 Z-score (PROTEIN)
D538G
E380Q
L536H
L536P
S463P
Y537C
Y537N
Y537S

Supplementary Figure 6. Transcript- and protein-centered evaluation of Estrogen Receptor levels.

ER transcript and protein levels were plotted against each other (panel A). Next, *ESR1* mutation AFs were plotted against transcript (panel B) and protein (panel C) abundances. In all panels, correlation coefficients were calculated using the Spearman method.

Acronyms: AF: allele frequency; ER: Estrogen Receptor.

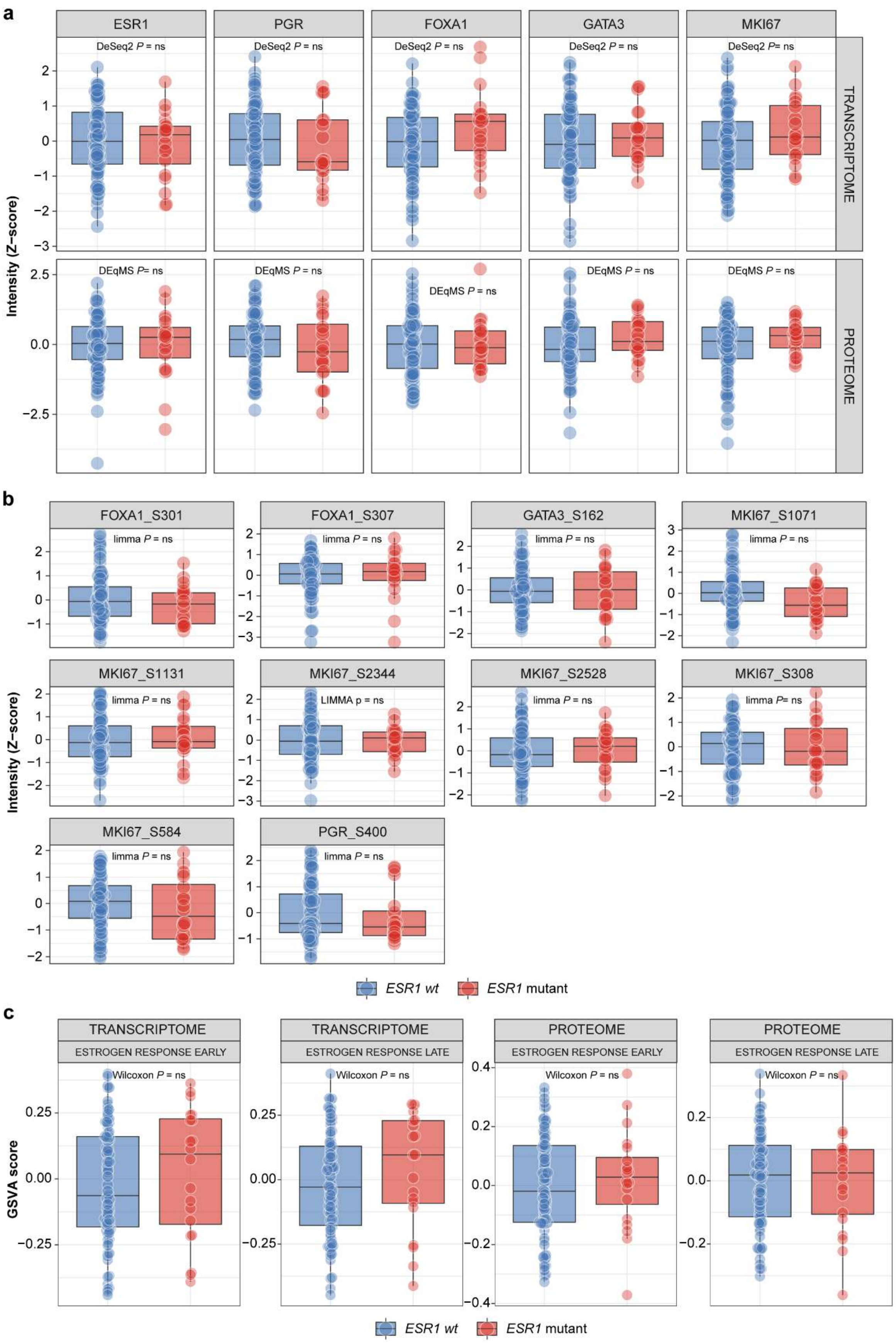
a
ESR1
PGR
FOXA1
GATA3
MKI67
TRANSCRIPTOME
PROTEOME
DeSeq2 P = ns
DEqMS P = ns
Intensity (Z-score)
b
FOXA1_S301
FOXA1_S307
GATA3_S162
MKI67_S1071
MKI67_S1131
MKI67_S2344
MKI67_S2528
MKI67_S308
MKI67_S584
PGR_S400
limma P = ns
LIMMA p = ns
Intensity (Z-score)
ESR1 wt
ESR1 mutant
c
TRANSCRIPTOME
ESTROGEN RESPONSE EARLY
ESTROGEN RESPONSE LATE
PROTEOME
Wilcoxon P = ns
GSVA score
ESR1 wt
ESR1 mutant

Supplementary Figure 7. Assessment of ER signaling related factors between *ESR1* mutated and *wt* tumors. Transcriptomic and proteomic data layers were interrogated for differences between *ESR1* mutants and *wt* tumors (transcriptome: DESeq2; proteome: DEQMS; phosphoproteome: limma). Panel A displays transcript (upper panels) and protein (lower panels) levels of *ESR1*, key ER co-factors, and tumor proliferation marker Ki67 in the two sample groups. Panel B displays phosphosite enrichments of the proteins reported in panel A. Panel C displays GSVA scores for the estrogen pathways (early and late) from the Hallmarks database (MSigdDB, v2024.01).

Acronyms: ER: Estrogen Receptor; GSVA: Gene Set Variation Analysis.

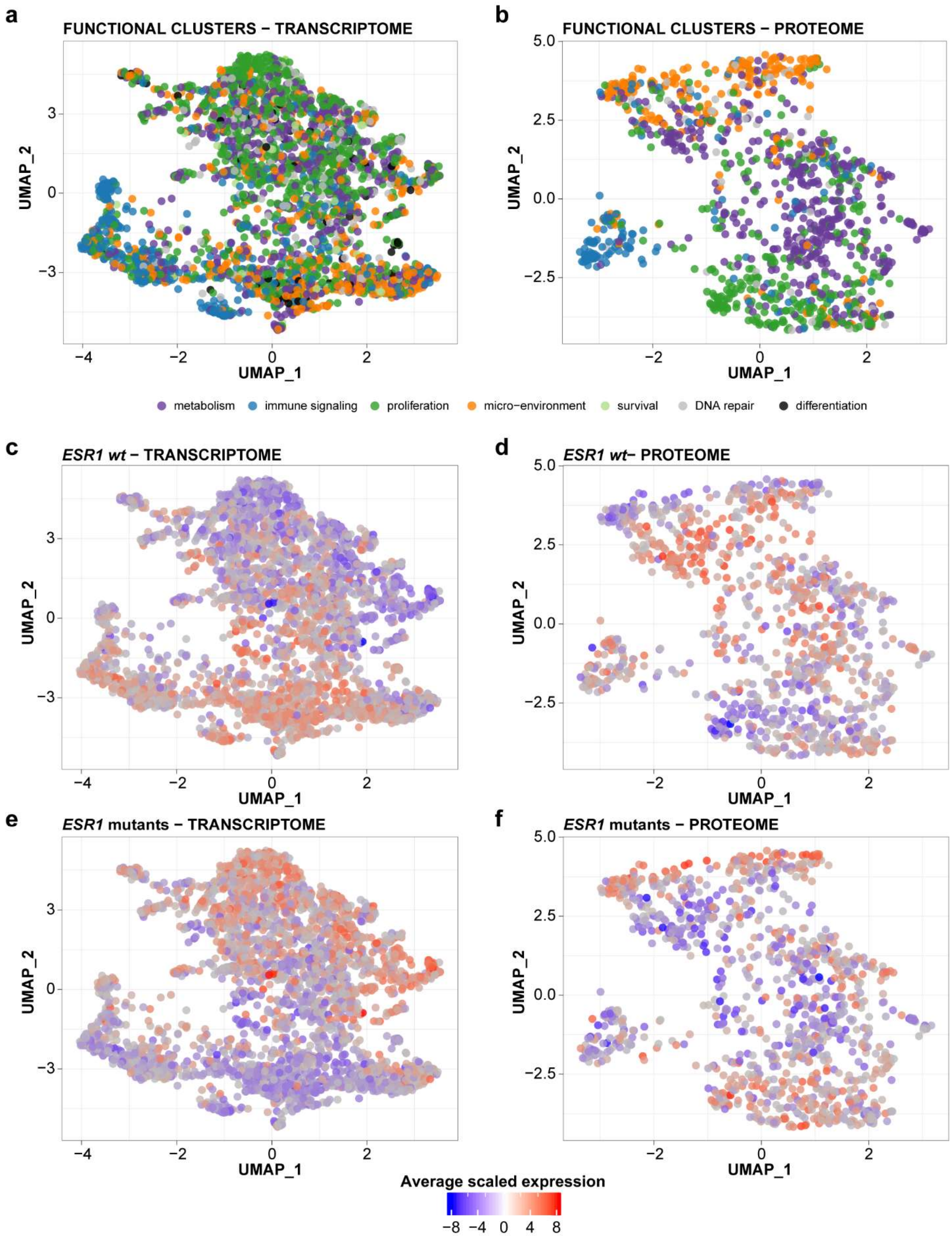

a
FUNCTIONAL CLUSTERS − TRANSCRIPTOME
b
FUNCTIONAL CLUSTERS − PROTEOME
UMAP_1
UMAP_2
metabolism
immune signaling
proliferation
micro−environment
survival
DNA repair
differentiation
c
ESR1 wt − TRANSCRIPTOME
d
ESR1 wt− PROTEOME
e
ESR1 mutants − TRANSCRIPTOME
f
ESR1 mutants − PROTEOME
Average scaled expression
−8 −4 0 4 8

Supplementary Figure 8. UMAP clustering of pathway-enriched genes and transcripts between *ESR1* mutants and *wt* tumors.

Transcripts and proteins belonging to significant pathways out of GSEA analyses of transcriptome and proteome data (FDR cutoff < 0.25; Pathways are depicted in Figure 4A) were selected and clustered using UMAP. Collapsing pathways-specific transcripts and proteins into functional units revealed distinct clusters of regulation (panels A and B). Average of transcript and protein abundances in *ESR1 wt* (panels C and D) and mutant tumors (panels E and F) was employed to identify changes in expression patterns.

Acronyms: FDR: False Discovery Rate; GSEA: Gene Set Enrichment Analysis; UMAP: Uniform Manifold Approximation and Projection.

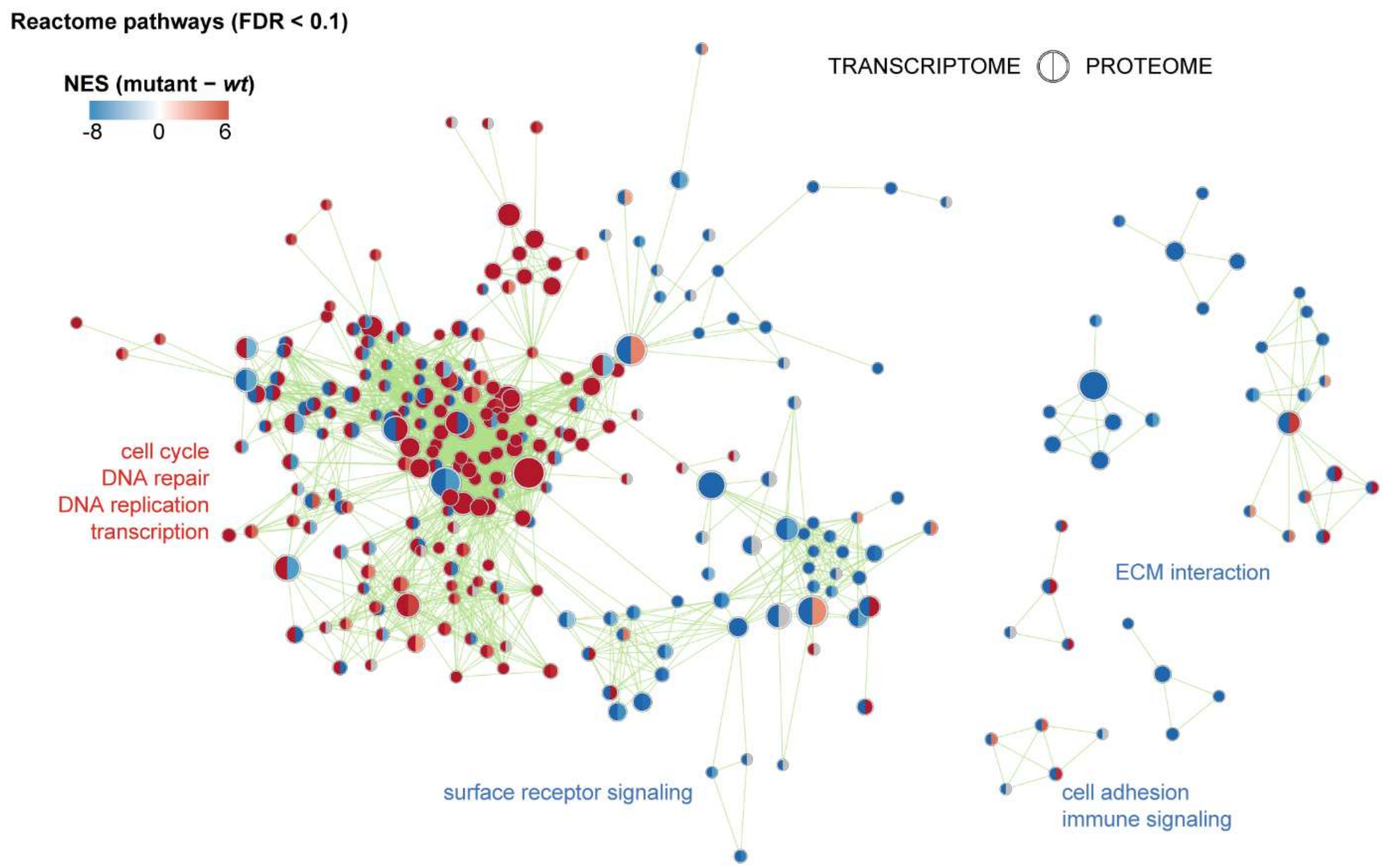
Reactome pathways (FDR < 0.1)
NES (mutant − wt)
-8 0 6
TRANSCRIPTOME PROTEOME
cell cycle
DNA repair
DNA replication
transcription
surface receptor signaling
ECM interaction
cell adhesion
immune signaling

Supplementary Figure 9. Reactome based enrichment map of *ESR1* mutations enriched pathways.

GSEA analyses of the transcriptome and proteome datasets for Reactome terms were imported (pathway FDR cutoff < 0.1) in Cytoscape using the Enrichment Map tool. Transcriptome and Proteome GSEA-derived NES was employed to color pathway nodes.

Acronyms: GSEA: Gene Set Enrichment Analysis; NES: Normalized Enrichment Score.

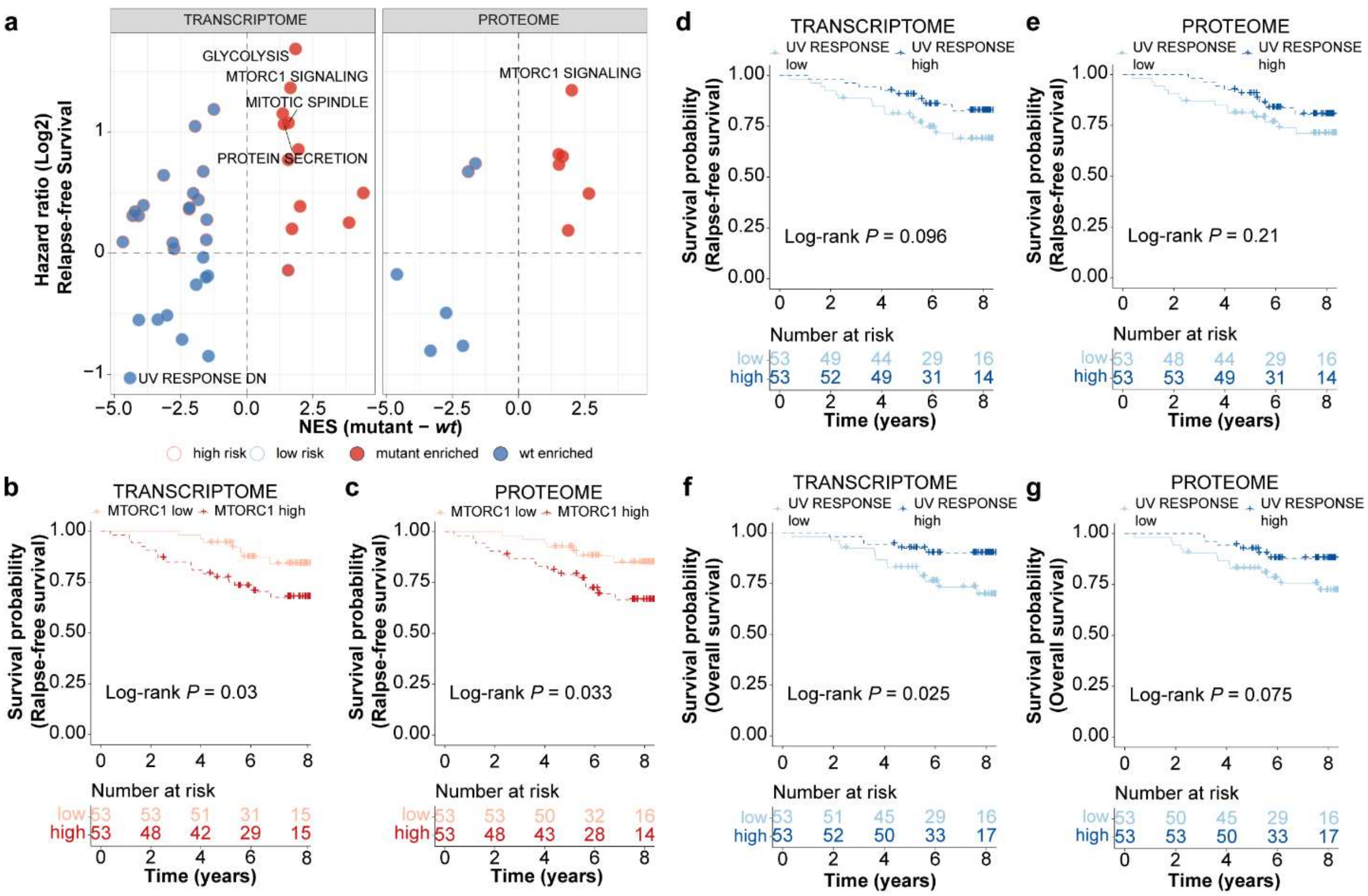
a
TRANSCRIPTOME
PROTEOME
GLYCOLYSIS
MTORC1 SIGNALING
MITOTIC SPINDLE
PROTEIN SECRETION
UV RESPONSE DN
Hazard ratio (Log2) Relapse-free Survival
NES (mutant − wt)
high risk
low risk
mutant enriched
wt enriched
b
TRANSCRIPTOME
MTORC1 low
MTORC1 high
Log-rank P = 0.03
c
PROTEOME
Log-rank P = 0.033
d
TRANSCRIPTOME
UV RESPONSE low
UV RESPONSE high
Log-rank P = 0.096
e
PROTEOME
Log-rank P = 0.21
f
TRANSCRIPTOME
Log-rank P = 0.025
g
PROTEOME
Log-rank P = 0.075
Survival probability (Ralpse-free survival)
Survival probability (Overall survival)
Number at risk
Time (years)

Supplementary Figure 10. Integrated analysis of pathway enrichment and association to prognosis in *ESR1* mutants.

GSVA scores of significant pathways out of GSEA analyses at the transcriptome and proteome levels (FDR < 0.25; Figure 4A) were calculated and tested for their association with RFS by Cox regression analysis. Log-transformed Cox regression-derived HRs were plotted against GSEA NES for each pathway (panel A). The most enriched (by NES) and prognosis-associated (by HR) pathways were selected and plotted in KM curves: panels B and C display transcriptome and proteome KM curves for the MTORC1 pathway and its association with RFS. Panels D and E display KM curves of the UV response pathway for RFS at the transcriptome and proteome level, while panels F and G represent the same analyses performed with OS as endpoint.

Acronyms: FDR: False Discovery Rate; GSEA: Gene Set Enrichment Analysis; GSVA: Gene Set Variation Analysis; HR: Hazard Ratio; KM: Kaplan-Meier; NES: Normalized Enrichment Score; OS: Overall Survival; RFS: Relapse-free Survival.

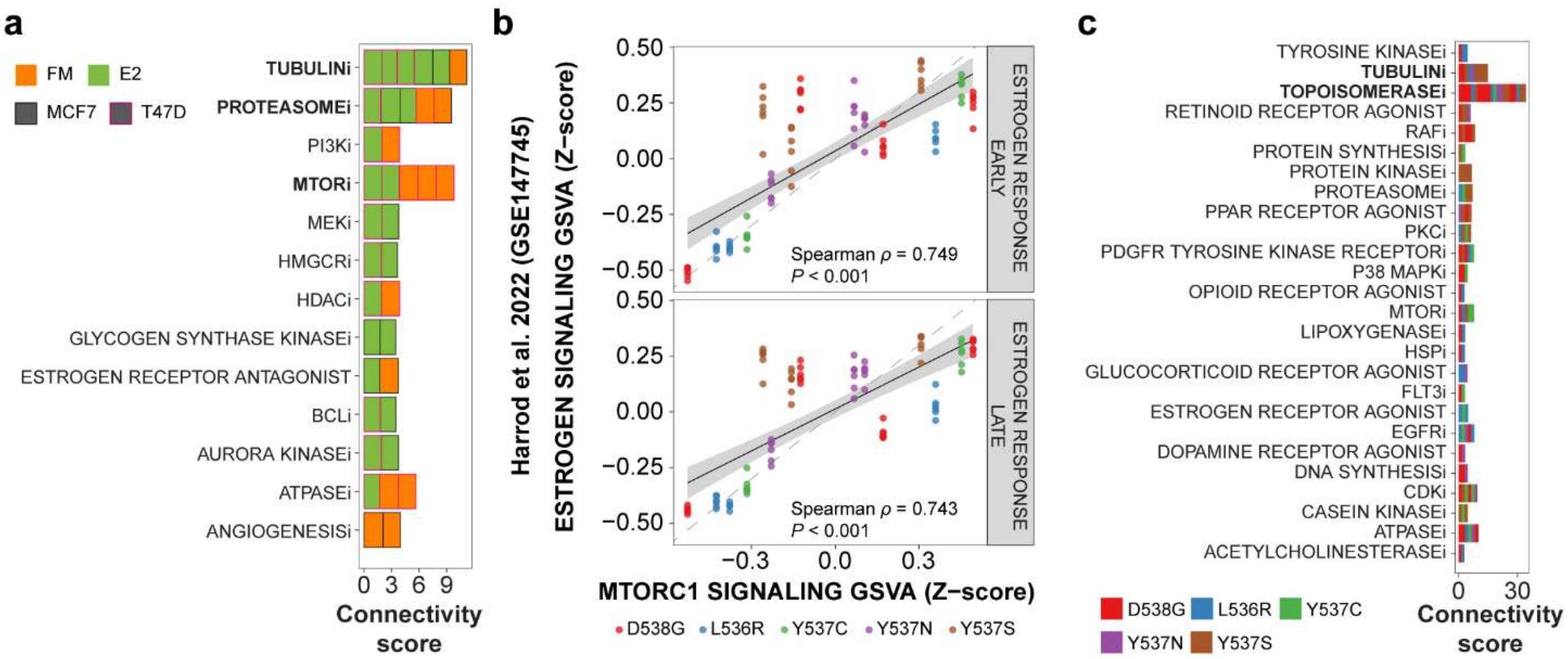
a
FM
E2
MCF7
T47D
TUBULINi
PROTEASOMEi
PI3Ki
MTORi
MEKi
HMGCRi
HDACi
GLYCOGEN SYNTHASE KINASEi
ESTROGEN RECEPTOR ANTAGONIST
BCLi
AURORA KINASEi
ATPASEi
ANGIOGENESISi
0 3 6 9
Connectivity score
b
Harrod et al. 2022 (GSE147745)
ESTROGEN SIGNALING GSVA (Z−score)
ESTROGEN RESPONSE EARLY
ESTROGEN RESPONSE LATE
Spearman ρ = 0.749
P < 0.001
Spearman ρ = 0.743
P < 0.001
0.50
0.25
0.00
−0.25
−0.50
−0.3 0.0 0.3
MTORC1 SIGNALING GSVA (Z−score)
D538G L536R Y537C Y537N Y537S
c
TYROSINE KINASEi
TUBULINi
TOPOISOMERASEi
RETINOID RECEPTOR AGONIST
RAFi
PROTEIN SYNTHESISi
PROTEIN KINASEi
PROTEASOMEi
PPAR RECEPTOR AGONIST
PKCi
PDGFR TYROSINE KINASE RECEPTORi
P38 MAPKi
OPIOID RECEPTOR AGONIST
MTORi
LIPOXYGENASEi
HSPi
GLUCOCORTICOID RECEPTOR AGONIST
FLT3i
ESTROGEN RECEPTOR AGONIST
EGFRi
DOPAMINE RECEPTOR AGONIST
DNA SYNTHESISi
CDKi
CASEIN KINASEi
ATPASEi
ACETYLCHOLINESTERASEi
0 30
Connectivity score
D538G L536R Y537C
Y537N Y537S

Supplementary Figure 11. Data-driven drug selection for phenotype reversal.

We employed Connectivity map to determine which drugs would reverse the *ESR1* phenotype passed on our proteomic dataset on MCF7 cells and the additional T47D set in our previous study. Connectivity scores for significant drug pathways were plotted in stacked bars (panel A). We analyzed the external RNAseq dataset of 15 *ESR1* mutant clones (GSEA147745) and plotted the correlations between the GSVA scores of the MTORC1 signaling and estrogen response (early and late) pathways (panel B). Connectivity map was employed too in this dataset to determine drug treatments for phenotype reversal (panel C).

Acronyms: GSVA: Gene Set Variation Analysis.

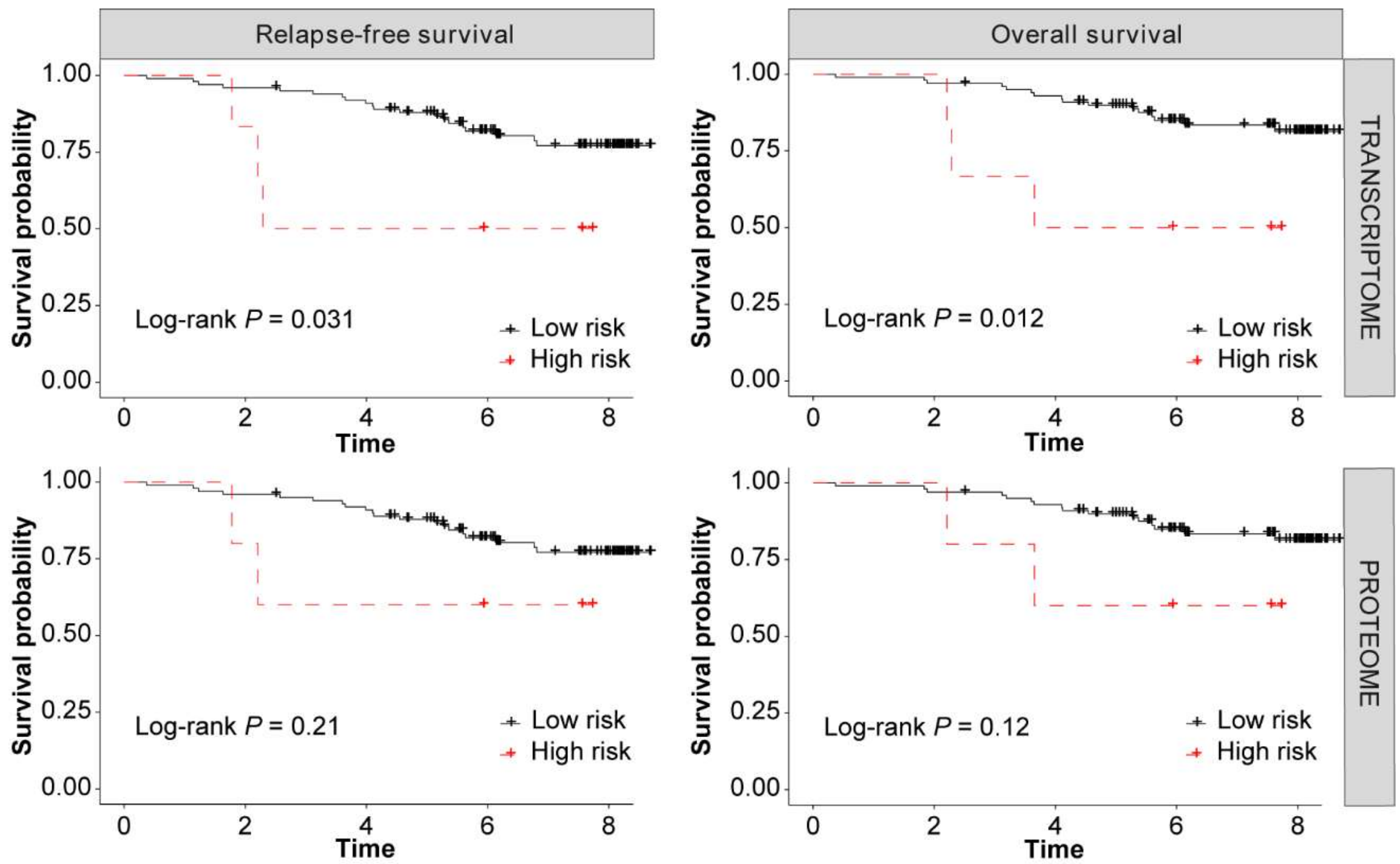
Relapse-free survival
Overall survival
TRANSCRIPTOME
PROTEOME
Survival probability
1.00
0.75
0.50
0.25
0.00
Time
0
2
4
6
8
Log-rank P = 0.031
Log-rank P = 0.012
Log-rank P = 0.21
Log-rank P = 0.12
Low risk
High risk

Supplementary Figure 12. Definition of high and low risk patients based on tumor grade, MTORC1 signaling, and *ESR1* mutations.

We combined tumor grade, *ESR1* mutational status, and GSVA-based MTORC1 signaling (transcriptome and protoeme) expression into a single score to define high and low risk patients. High MTORC1 expression (based on median dichotomization), high grade, and expression of an ER mutations were combined together to define high risk patients, while all other patients were grouped into the low risk group. KM plots and Log-rank tests were employed to test for significant differences in RFS (left panels) and OS (right panels).

Acronyms: GSVA: Gene Set Variation Analysis; KM: Kaplan-Meier; OS: Overall Survival; RFS: Relapse-free survival.

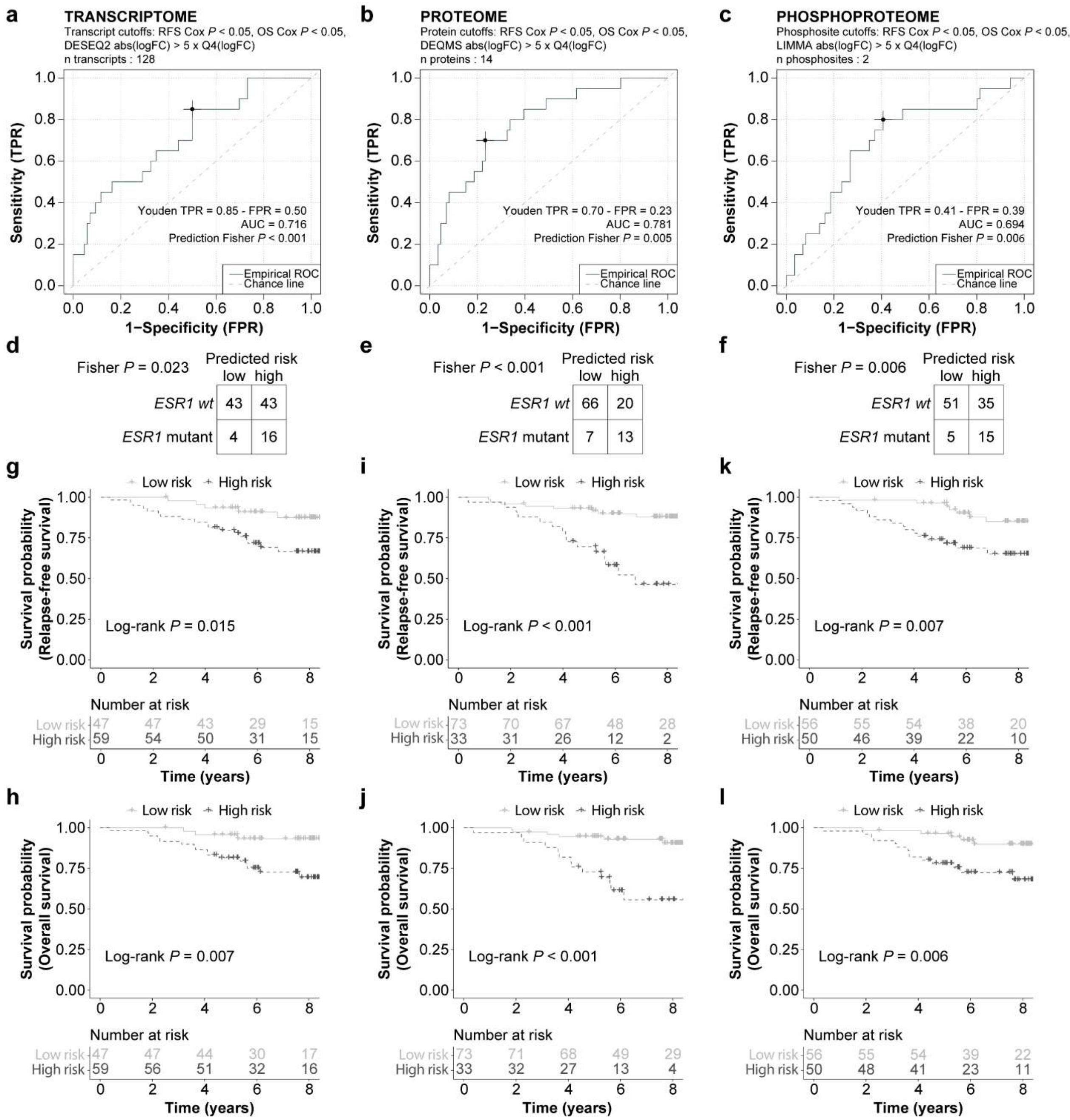
a
TRANSCRIPTOME
Transcript cutoffs: RFS Cox P < 0.05, OS Cox P < 0.05, DESEQ2 abs(logFC) > 5 x Q4(logFC)
n transcripts : 128
Youden TPR = 0.85 - FPR = 0.50
AUC = 0.716
Prediction Fisher P < 0.001
b
PROTEOME
Protein cutoffs: RFS Cox P < 0.05, OS Cox P < 0.05, DEQMS abs(logFC) > 5 x Q4(logFC)
n proteins : 14
Youden TPR = 0.70 - FPR = 0.23
AUC = 0.781
Prediction Fisher P = 0.005
c
PHOSPHOPROTEOME
Phosphosite cutoffs: RFS Cox P < 0.05, OS Cox P < 0.05, LIMMA abs(logFC) > 5 x Q4(logFC)
n phosphosites : 2
Youden TPR = 0.41 - FPR = 0.39
AUC = 0.694
Prediction Fisher P = 0.006
Sensitivity (TPR)
1−Specificity (FPR)
Empirical ROC
Chance line
d
Fisher P = 0.023
Predicted risk low high
ESR1 wt 43 43
ESR1 mutant 4 16
e
Fisher P < 0.001
Predicted risk low high
ESR1 wt 66 20
ESR1 mutant 7 13
f
Fisher P = 0.006
Predicted risk low high
ESR1 wt 51 35
ESR1 mutant 5 15
g
Low risk High risk
Survival probability (Relapse-free survival)
Log-rank P = 0.015
Number at risk
Low risk 47 47 43 29 15
High risk 59 54 50 31 15
Time (years)
i
Log-rank P < 0.001
Low risk 73 70 67 48 28
High risk 33 31 26 12 2
k
Log-rank P = 0.007
Low risk 56 55 54 38 20
High risk 50 46 39 22 10
h
Survival probability (Overall survival)
Log-rank P = 0.007
Low risk 47 47 44 30 17
High risk 59 56 51 32 16
j
Log-rank P < 0.001
Low risk 73 71 68 49 29
High risk 33 32 27 13 4
l
Log-rank P = 0.006
Low risk 56 55 54 39 22
High risk 50 48 41 23 11

Supplementary Figure 13. Transcriptomic, proteomic, and phosphoproteomic signatures.

Transcript, proteins, and phosphosites were selected based on their significant association to RFS and OS by Cox regression analysis ($p < 0.05$) and based on their enrichment in either group (*wt ESR1* or mutant). For each data layer, a ROC model was generate based on the selected features and Youden index was selected as cutoff for group dichotomization (transcriptomics n = 128; proteomics n = 14; phosphoproteomics n = 2; panels A-C). Performance in detecting *ESR1* mutant tumors was assessed for each model by employing contingency tables and Fisher's exact test (panels D-F). KM curves and Log-rank tests were generated to assess the predicted groups for RFS (panels G, I, K) and OS (panels H, J, L).

Acronyms: KM: Kaplan-Meier; OS: Overall Survival; RFS: Relapse-free survival.

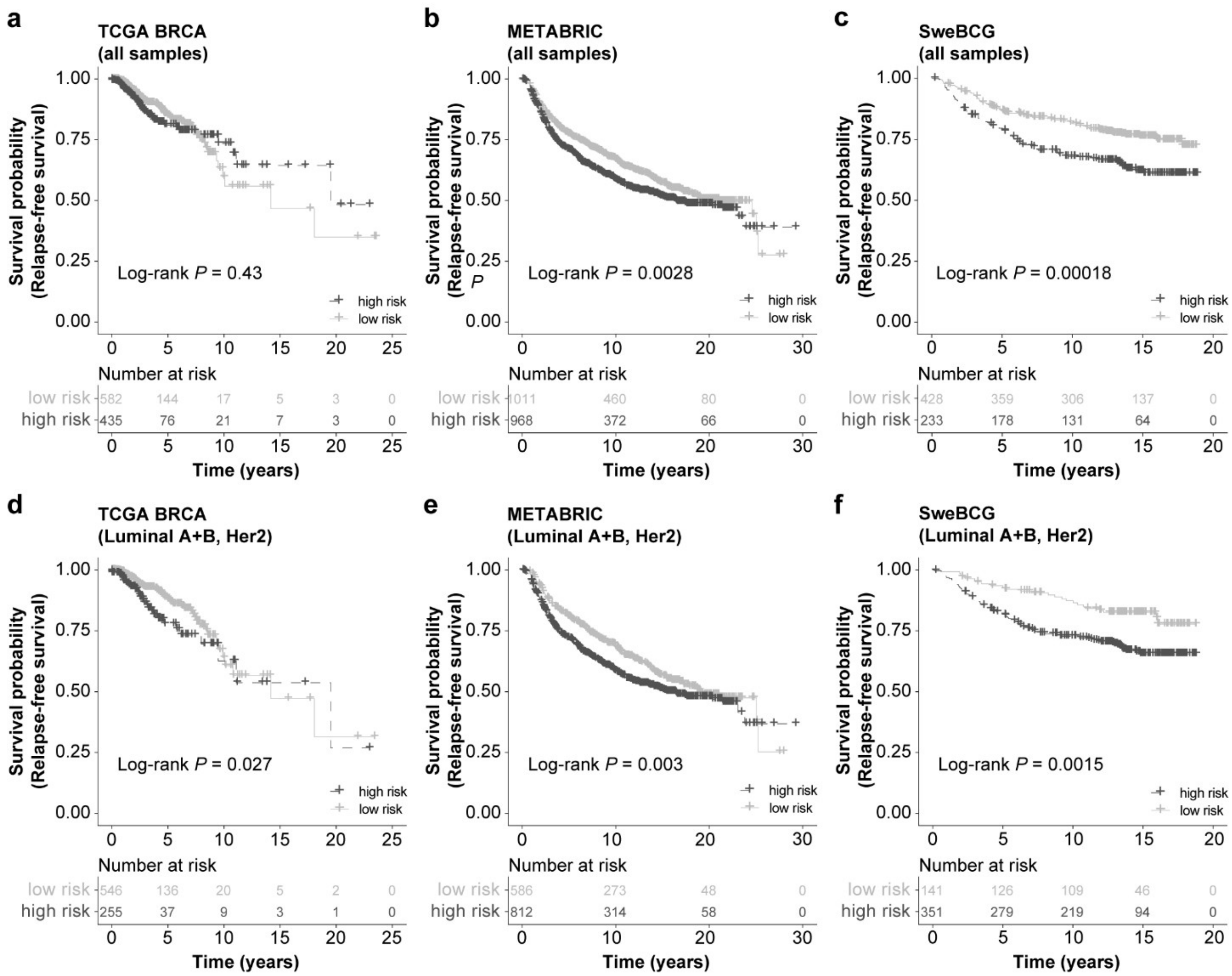
a
TCGA BRCA
(all samples)
Log-rank P = 0.43
b
METABRIC
(all samples)
Log-rank P = 0.0028
c
SweBCG
(all samples)
Log-rank P = 0.00018
d
TCGA BRCA
(Luminal A+B, Her2)
Log-rank P = 0.027
e
METABRIC
(Luminal A+B, Her2)
Log-rank P = 0.003
f
SweBCG
(Luminal A+B, Her2)
Log-rank P = 0.0015
Survival probability
(Relapse-free survival)
high risk
low risk
Number at risk
Time (years)

Supplementary Figure 14. Kaplan-Meier curves of the RNA signature predicted groups in the TCGA, METABRIC, and SweBCG cohorts.

Panels A to C display KM plots for RFS in all samples of the TCGA, METABRIC, and SweBCG cohorts, respectively. Panels D to F display the results of these cohorts' subset including Luminal A/B and Her2 tumors. Log-rank test was used to evaluate significant differences between the survivals of the predicted patient groups.

Acronyms: KM: Kaplan-Meier; RFS: Relapse-free survival; TCGA: The Cancer Genome Atlas.

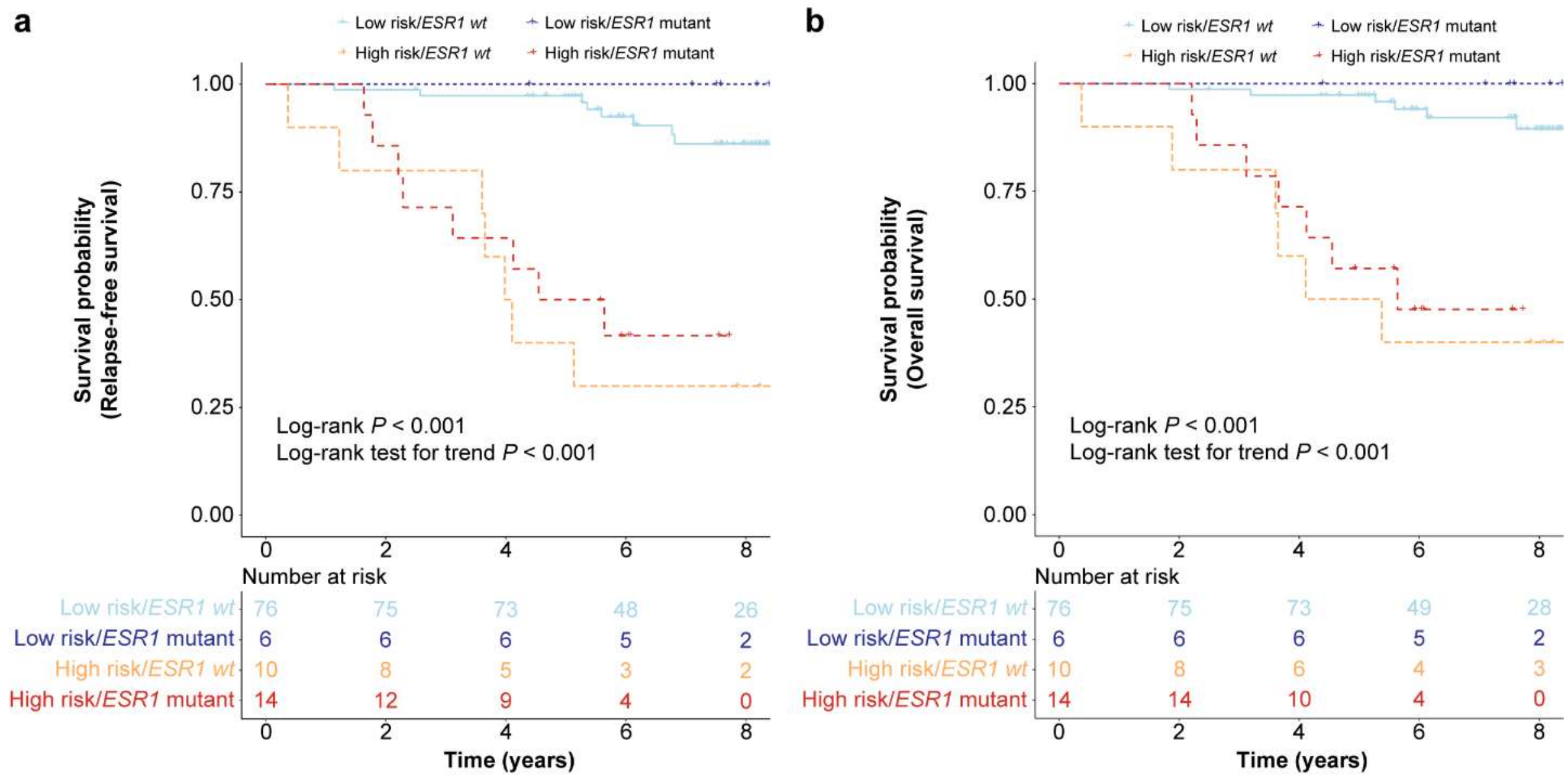
a
Low risk/ESR1 wt
Low risk/ESR1 mutant
High risk/ESR1 wt
High risk/ESR1 mutant
Survival probability (Relapse-free survival)
1.00
0.75
0.50
0.25
0.00
Log-rank P < 0.001
Log-rank test for trend P < 0.001
0 2 4 6 8
Number at risk
Low risk/ESR1 wt 76 75 73 48 26
Low risk/ESR1 mutant 6 6 6 5 2
High risk/ESR1 wt 10 8 5 3 2
High risk/ESR1 mutant 14 12 9 4 0
Time (years)
b
Low risk/ESR1 wt
Low risk/ESR1 mutant
High risk/ESR1 wt
High risk/ESR1 mutant
Survival probability (Overall survival)
Log-rank P < 0.001
Log-rank test for trend P < 0.001
Number at risk
Low risk/ESR1 wt 76 75 73 49 28
Low risk/ESR1 mutant 6 6 6 5 2
High risk/ESR1 wt 10 8 6 4 3
High risk/ESR1 mutant 14 14 10 4 0
Time (years)

Supplementary Figure 15. Kaplan-Meier curves of the proteogenomic predictor in combination with *ESR1* mutational status.

Panels A and B display KM plots for RFS and OS for the proteogenomic predictor in combination with *ESR1* mutational status. Log-rank test and Log-rank test for trend was used to evaluate significant differences between the survivals of the patient groups.

Acronyms: KM: Kaplan-Meier; OS: Overall Survival; RFS: Relapse-free survival.

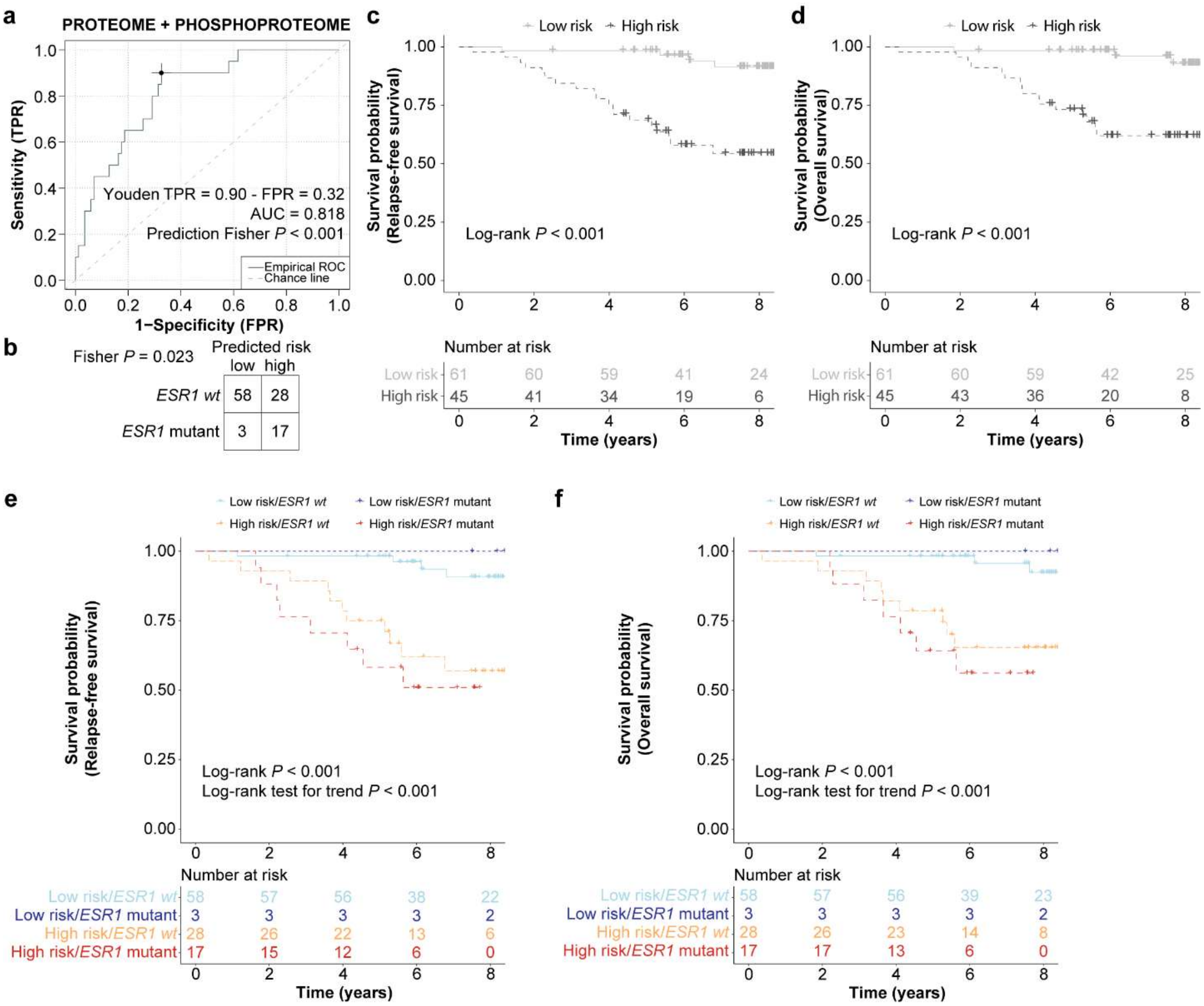
a
PROTEOME + PHOSPHOPROTEOME
Sensitivity (TPR)
1−Specificity (FPR)
Youden TPR = 0.90 - FPR = 0.32
AUC = 0.818
Prediction Fisher P < 0.001
Empirical ROC
Chance line
b
Fisher P = 0.023
Predicted risk
low high
ESR1 wt 58 28
ESR1 mutant 3 17
c
Low risk High risk
Survival probability (Relapse-free survival)
Log-rank P < 0.001
Number at risk
Low risk 61 60 59 41 24
High risk 45 41 34 19 6
Time (years)
d
Low risk High risk
Survival probability (Overall survival)
Log-rank P < 0.001
Number at risk
Low risk 61 60 59 42 25
High risk 45 43 36 20 8
Time (years)
e
Low risk/ESR1 wt
Low risk/ESR1 mutant
High risk/ESR1 wt
High risk/ESR1 mutant
Survival probability (Relapse-free survival)
Log-rank P < 0.001
Log-rank test for trend P < 0.001
Number at risk
Low risk/ESR1 wt 58 57 56 38 22
Low risk/ESR1 mutant 3 3 3 3 2
High risk/ESR1 wt 28 26 22 13 6
High risk/ESR1 mutant 17 15 12 6 0
Time (years)
f
Low risk/ESR1 wt
Low risk/ESR1 mutant
High risk/ESR1 wt
High risk/ESR1 mutant
Survival probability (Overall survival)
Log-rank P < 0.001
Log-rank test for trend P < 0.001
Number at risk
Low risk/ESR1 wt 58 57 56 39 23
Low risk/ESR1 mutant 3 3 3 3 2
High risk/ESR1 wt 28 26 23 14 8
High risk/ESR1 mutant 17 17 13 6 0
Time (years)

Supplementary Figure 16. Protein-centric model for prediction of breast cancer prognosis.

Significant (see *Methods* and Supplementary Figure 5 for details) proteins and phosphosites were selected and merged into a multi-omic signature (panel A). The predictive model significantly detected *ESR1* tumors (panel B). KM plots for RFS (panel C) and OS (panel D) also showed good performance of the predictive model. Panels E and F display KM curves of predictor groups in combination with *ESR1* mutational status. Differences in survival between groups were evaluated using the Log-rank test and Log-rank test for trend.

Acronyms: KM: Kaplan-Meier; OS: Overall Survival; RFS: Relapse-free survival.
