# Supplementary material for: ESR1 mutations are associated with enhanced MTORC1 signaling in a context-dependent manner
Source: Commun Biol. 2026 Oct 7;9:1304. doi: 10.1038/s42003-026-11096-w (PMC13645737; doi:10.1038/s42003-026-11096-w)
Supplement: Supplementary file 3 [file 42003_2026_11096_MOESM3_ESM.pdf]

Description of Additional Supplementary File

File name: Supplementary Data 1

Description: Contains Patient information

File name: Supplementary Data 2

Description: Contains List of features in predictive models

File name: Supplementary Data 3

Description: This file contain the source file for all main figure graphs
